# Sesquiterpenoids from Meliaceae Family and Their Biological Activities

**DOI:** 10.3390/molecules28124874

**Published:** 2023-06-20

**Authors:** Sandra Amalia Riyadi, Al Arofatus Naini, Unang Supratman

**Affiliations:** 1 Department of Chemistry, Faculty of Mathematic and Natural Sciences, Universitas Padjadjaran, Jatinangor, Sumedang 45363, Indonesia; s.amaliariyadi89@gmail.com (S.A.R.); alarofatusnaini@gmail.com (A.A.N.); 2 Central Laboratory, Universitas Padjadjaran, Jatinangor, Sumedang 45363, Indonesia

**Keywords:** sesquiterpenoids, Meliaceae, biological activities, eudesmane-type

## Abstract

Sesquiterpenoids, an important class of natural products possessing three isoprene-derived units, are widely distributed across plants and have a variety of biological activities. All sesquiterpenoids are derived from farnesyl pyrophosphate (FPP), a biosynthesis precursor that can form various carbon skeletons. In order to provide a reference for further research and development of these compounds, this review focused on the increasing number of isolated and volatile sesquiterpenoids found to be produced by plants of the Meliaceae family between 1968 and 2023. The related articles were collected from SciFinder, Google Scholar, and PubMed. According to a literature review, several studies were started for more than 55 years on the plant’s stem barks, twigs, leaves, flowers, seeds, and pericarps, where approximately 413 sesquiterpenoid compounds from several groups such as eudesmane, aromadendrane, cadinane, guaiane, bisabolane, furanoeremophilane, humulene, germacrane, and oppositane-type were isolated and identified with some minor products. Additionally, the hypothetical route of sesquiterpenoids biosynthesis from this family was identified, and eudesmane-type was reported to be 27% of the total compounds. The antimicrobial, antidiabetic, antioxidant, antiplasmodial, antiviral, and cytotoxic activities of the isolated compounds and major volatile sesquiterpenoids constituent on essential oil were also evaluated. The result showed the fundamental of using the sesquiterpenoid compounds from the Meliaceae family in traditional medicine and the discovery of new drugs.

## 1. Introduction

Meliaceae are woody plants found in the tropics and some cooler areas than equatorial zones that are known for their high-quality wood. There are 740 species in 58 genera in the Meliaceae family, which belongs to the order Sapindales, throughout the Malaya-Indo region, Africa-Madagascar, and Australia-Asia [1]. Due to limited plant distribution and plant resources, only 24 genera have been studied in recent years [2]. From the phytochemical investigation of some members of the family Meliaceae, some natural products have been identified. These include sesquiterpenoids [3,4,5,6], diterpenoids [7,8,9,10], triterpenoids [11,12,13,14,15], limonoids [16,17,18,19,20], lignans [21,22], flavaglines [23], and alkaloids [24,25]. Furthermore, numerous biologically active compounds were isolated, including cytotoxic compounds [26], antifeedant, antiinflammation [27], antiviral, antimicrobial [28], and antidiabetic, and were also investigated.

The sesquiterpenoids represent a large fraction of naturally occurring terpenes. Many of the sesquiterpenoids are found in higher plants and possess important uses both in flavor and fragrance [29]. Consequently, many sesquiterpenoid compounds are derived from steam distillation fractions and possess fragrant characteristics. It is possible to identify non-volatile sesquiterpenoids by using NMR analysis and volatile sesquiterpenoids using the Gas Chromatography–Mass Spectroscopy (GC-MS) technique [30]. Furthermore, the first study on sesquiterpenoids in Meliaceae family was conducted in 1968 with the isolation of several alcohol sesquiterpenoids, including T-muurolol (**148**), cubenol (**118**), and epi-cubenol (**119**) and a major hydrocarbon sesquiterpenoid namely copaene (**192**) from the timber of *Cedrela toona* Robx [31]. After 55 years, various classes of sesquiterpenoids with the eudesmane-type bicyclic isolated sesquiterpenoids as the main component and the chemical marker were identified. They have extensive biological functions such as cytotoxicity, antiplasmodial, antimicrobial, antidiabetic, anti-viral, and anti-inflammation. In addition, this review also concerns volatile sesquiterpenoids for their chemotaxonomically, ecological, and drug development implications through the biological activities of major sesquiterpenoid constituents. Volatile sesquiterpenoids themselves can also describe variations in composition based on where the plants are grown [32]. These are mainly sesquiterpene hydrocarbons, mono-oxygenated sesquiterpenoids, sesquiterpenoid epoxides, and sesquiterpenoid ketones. The diversity of volatile sesquiterpenoids reported from numerous parts of plants Meliaceae family and their essential oil bioactivities are presented. Since there was no comprehensive study on the sesquiterpenoids from the Meliaceae family, it is necessary to develop a comprehensive summary that consists of the traditional application, chemical content, and biological aspects of these compounds. Therefore, this study was the first summary that covers a total of 211 isolated and 202 volatile sesquiterpenoids with a grouping of each framework, each type, the ethnobotanical, and their biological activities. The plausible biogenetic pathways of each sesquiterpenoid type, the differences in its skeleton, and its potential from Meliaceae as promising compounds for anticancer discovery were identified. This is expected to be a foundation for further studies in the discovery of new drugs.

## 2. Methodology and Botany

This study searched for different literature relating to sesquiterpenoids in Meliaceae, and a plant database, namely www.theplantlist.org. It also involved related articles from 1968 to 2023, which were collected from SciFinder, PubMed, Google Scholar, and Scopus. Moreover, the sesquiterpenoids were classified based on their phytochemical, ethnobotanical, and biological properties.

The Meliaceae family consists of over 740 species within 58 genera of trees and shrubs. Ecologically, trees and shrubs of the Meliaceae family grow in a wide variety of habitats, including rain forests, semi-deserts, and mangrove swamps. Meliaceae are important components in tropical ecosystems worldwide, especially lowland forests. They are common in lowland rainforests, including *Carapa*, *Guarea*, *Trichilia*, and *Aglaia*; mountain rainforests, including *Dysoxylum* and *Cedrela*; and tropical deciduous forests, namely *Cedrela*, *Swietenia*, and *Trichilia*. Species of some genera also occur in rough scrub or rocky hillsides *Swietenia* [1]. 

In several studies, the Meliaceae family has been found to be naturally distributed in Indo-Malesia and southern China, including *Cedrela*, *Dysoxylum*, *Cipadessa*, *Amoora*, and *Aphanamixis*. Moreover, some genera are also widely distributed not only in Indo-Malesia, but also in Europe and Northern Australia, including *Toona* and *Aglaia*, and throughout Africa, including *Ekebergia*, *Etandrophragma*, *Trichilia*, and *Turraea* [1,26,28]. 

**Figure 1:**
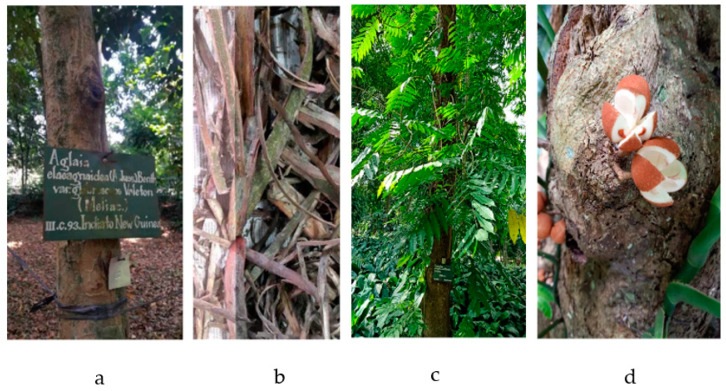
The bark of *Aglaia elaeagnoidea* (**a**), dried stembarks of *Aglaia elaeagnoidea* (**b**), the tree of *Dysoxylum parasiticum* (**c**), and the fruits of *Dysoxylum parasiticum* (**d**). Photographs courtesy of Mr. Harto of the Bogoriense Herbarium.

A tree of this family is commonly over 100 feet tall and 4 to 5 feet wide, with a straight, cylindrical bole exceeding 40 to 60 feet, sometimes buttressed to a height of 10–15 feet (Figure 1). The leaves are alternate, pinnate, pentamerous, and paniculate. The fruit is a five-celled (rarely four-celled), five-valved (rarely four-valved), woody capsule with 10–14 seeds in each cell [33]. 

## 3. Phytochemistry

### 3.1. Overview of the Sesquiterpenoids Isolated from Meliaceae Family

**Figure 2:**
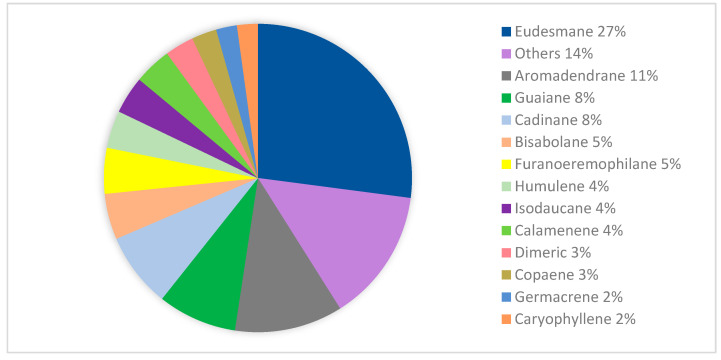
The distribution of sesquiterpenoids-type isolated from the Meliaceae family.

During the past decade, based on the literature collected from 1968 to 2023, a total of 211 isolated and 202 volatile sesquiterpenoids were gained from leaves, barks, stem barks, fruits, pericarps, roots, twigs, and flowers of the Meliaceae family. Structurally, sesquiterpenoid frameworks were classified as acyclic, monocyclic, bicyclic, tricyclic, and other frameworks (dimeric and trimeric). Moreover, monocyclic frameworks consist of bisabolane, humulene, ionone, and megastigmane type, and bicyclic frameworks consist of eudesmane, guaiane, calamenene, caryophyllene, hydro-azulene, murolene, himachalane, oppositane, and sabinene type. Tricyclic sesquiterpenoids are also found, including aromadendrane, copaene, clovane, and furanoeremophilane. Based on Figure 2, the eudesmane-type is the largest isolated product, with a total of 62 compounds (27%), followed by other minor sesquiterpenoids-type (14%), and another major sesquiterpenoids types, including aromadendrane (11%), cadinane (8%), guaiane (8%), bisabolane (5%), furanoeremophilane (5%), humulene (4%), isodaucene (4%), caryophyllene (2%), germacrane (2%), and oppositane (2%). 

Acyclic and cyclic sesquiterpenoids can be formed by farnesyl diphosphate (FPP). With the added bond and increased chain length, the number of possible cyclization modes increases, and a wide range of mono-, bi-, and tricyclic structures are possible. Sesquiterpenoid frameworks can be classified into acyclic, monocyclic, bicyclic, tricyclic, and multicyclic based on their carbon ring number. Acyclic sesquiterpenoids can be formed by modifying *trans*-farnesyl cation or *cis*-farnesyl cation. In order to form monocyclic sesquiterpenoids, FPP is ionized to produce a *trans*-allylic cation, which is then isomerized to a *cis*-allylic cation. Further cyclization of the *cis*-allylic cation forms the bisabolyl cation with the six-membered ring system. Furthermore, this carbocation is dehydrogenated or attacked by water molecules to form the general structure of the bisabolane type [34].

**Figure 3:**
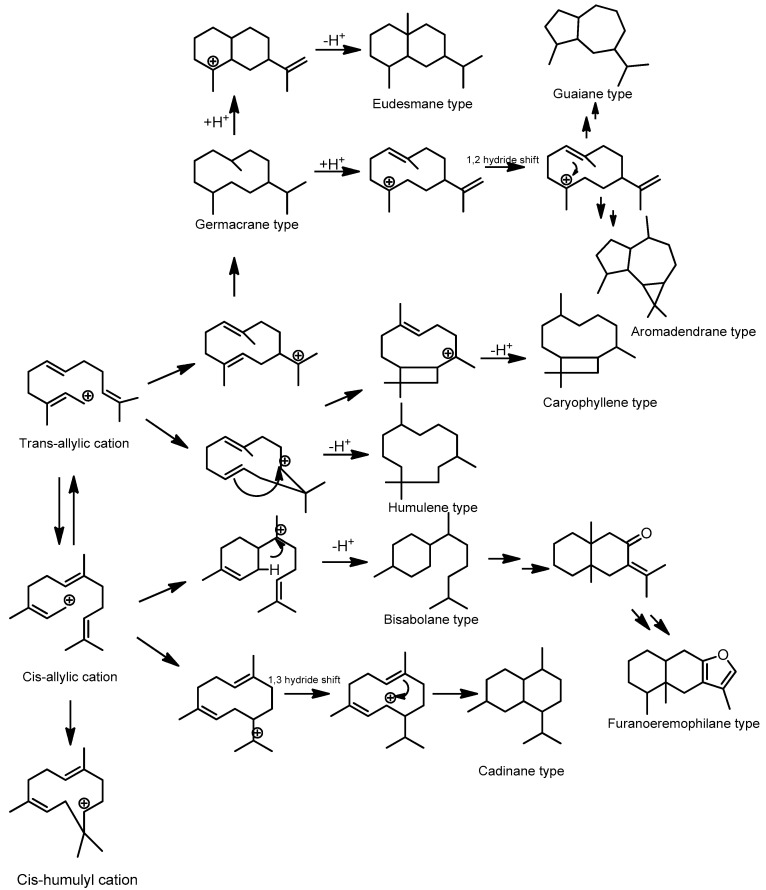
Plausible biosynthetic pathways of sesquiterpenoids from Meliaceae family.

Humulene-type is a monocyclic sesquiterpenoid that transformed from FPP by cyclization of *trans*-allylic cation or *cis*-allylic cation to form humulyl cation with the 11-membered ring system or *cis*-humulyl cation, differ only in the stereochemistry associated with the double bond [35]. Moreover, further cyclization by an attack on the 2,3-double bond and removal of a proton from the attached methyl group can lead to the caryophyllene type. The germacryl cation, without further cyclization, is a precursor of the germacrene-type of sesquiterpenoids. The eudesmane-type sesquiterpenoids can be formed by further cyclization of FPP to the eudesmane cation through germacrane derivative with further cyclization by various enzymatic reactions. Moreover, guaiane-type of sesquiterpenoids were formed by protonation of germacrene derivative followed by 1,2-hydride shift and the loss of a proton. The *cis*-allylic cation derived from FPP undergoes initial ring closure to generate germacrene intermediate, a 1,3-hydride shift repositions the final cadinane type sesquiterpenoid. As tricyclic sesquiterpenoids, aromadendrane-types are derived from germacrane derivatives and undergo several cyclizations and oxidations. Moreover, by the enzymatic reaction, the bisabolane derivative can be transformed into an eremophilane skeleton, which is a precursor for the formation of furanoeremophilane-type sesquiterpenoids by the further hydroxylation mechanism [36]. Moreover, the relationships between biosynthetic pathways and the differences in each framework of the major type sesquiterpenoids in Meliaceae are shown in Figure 3.

### 3.2. Isolated Sesquiterpenoids

This section comprises isolated sesquiterpenoids extracted from the dried part of plants. Solid–liquid extraction by organic solvents is the main technique to remove the soluble compounds into the liquid phase. The extracts are then further chromatographed to discover a single compound and characterized by spectroscopic methods and X-ray diffraction, as well as by comparing the NMR shifts with the related reports. 

#### 3.2.1. Acyclic Sesquiterpenoids 

**Figure 4:**
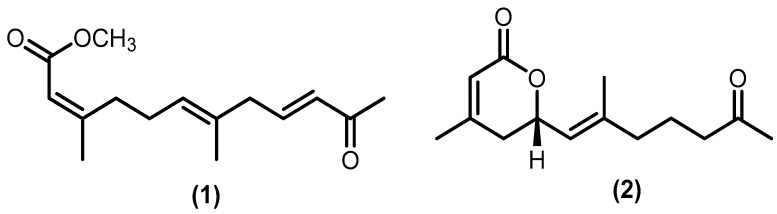
Isolated acyclic sesquiterpenoids.

Only two acyclic sesquiterpenoids were identified: aphanamoxene D (**1**) and nemoralisin D (**2**). The norsesquiterpene derivative aphanamoxene D was isolated from the ethanol extract of *Aphanamixis polystachya* (Wall.) R.Parker, while the norsesquiterpene derivative nemoralisin D was formed after highly oxidizing nemoralisin in the methanol extract of *Aphanamixis grandifolia* Blume [37,38]. The structure of the acyclic type (**1**–**2**) is shown in Figure 4. 

#### 3.2.2. Monocyclic Sesquiterpenoids 

**Figure 5:**
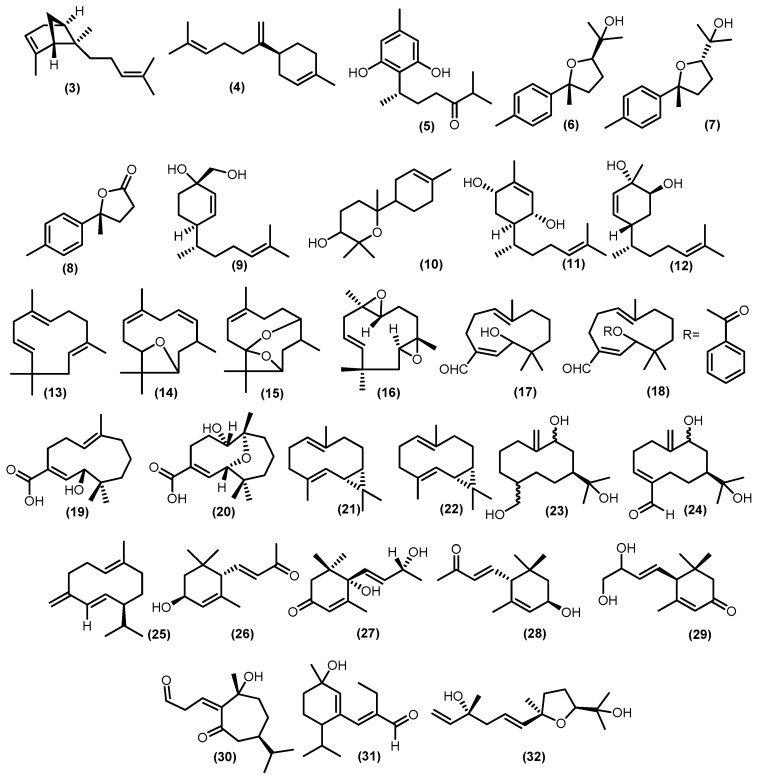
Isolated monocyclic sesquiterpenoids.

There were monocyclic sesquiterpenoids found in previous studies on Meliaceae, 10 bisabolane-type sesquiterpenoids have been discovered (Figure 5). A study conducted by Krishnappa and friends showed that there are two bisabolane-type metabolites, namely *α*-*trans*-bergamotene (**3**) and *β*-bisabolene (**4**), isolated from wood *Lansium anamalayanum* Bedd. and these compounds were investigated by IR and NMR spectra and comparison with related compounds [39]. In addition, more research identified schiffnerone A (**5**), which was obtained from the wood of *Dysoxylum schiffneri* F. Muell. [40]. There were also three bisabolene-type sesquiterpenoids produced eight years later from stembark of *Cipadessa boiviana* Baill., namely 7,10*β*-epoxy-1,3,5-bisabolatrien-11-ol (**6**); 7,10*α*-epoxy-1,3,5-bisabolatrien-11-ol (**7**), which is racemic product; and 11,12,13-trisnorbisabola-1,3,5-trien-10,7-olide (**8**), which is a modified bisabolene-type, known as *trisnor*-bisabolane [41]. The zingiberenol-type of monocyclic sesquiterpenoid, which had a related structure to bisabolene-type sesquiterpenoid (6*R*,7*R*,10*S*)-15-hydroxy-zingiberenol (**9**) isolated from the stems of *Dysoxylum oliganthum* C.Y.Wu and it was determined by CD spectra data for its absolute configuration [42]. A bisabolol oxide A (**10**) was isolated from the stembark of *Aglaia elaeagnoidea* (A.Juss.) Benth. [43], and (1*R*,4*S*,6*R*)-1,4-dihydroxybisabola-2,10-diene (**11**), (3*S*,4*S*,6*R*)-3,4-dihydroxybisabola-1,10-diene (**12**) from the twigs and leaves *Aglaia lawii* (Wight) C.J.Saldanha [44] were also discovered in this genus. 

The occurrence of humulene, also called *α*-humulene (**13**), is one of the most common sesquiterpenes, and it is widely distributed among plant species in the Meliaceae family, including the stembark of *Guarea macrophylla* M. Vahl, *Guarea guidonia* (L.) Sleumer, and stems of *Trichilia lepidota* Mart. [45,46,47]. The oxygenated derivatives of humulene were plentiful in 1998 [48]. Among them are 1(10)-epoxy-4,7-humuladiene (**14**) and 1(10),4-diepoxy-7-humulene (**15**) from *Guarea guidonia* wood bark. Mahdzir et al. reported another oxygenated humulene from the bark of *Walsura pinata* Hassk. 2(3),6(7)-diepoxy-9-humulene (**16**) [49]. Kurubasch aldehyde (**17**), a sesquiterpenoid with a hydroxylated humulene skeleton, was discovered for the first time in *Trichilia* genus. During this time, a derivative called kurubasch aldehyde esters (**18**) was gained from the roots of *Trichilia emetica* (Forssk.) Vahl [5]. The modification of the carbon skeleton of the humulene type usually leads to the formation of analog compounds such as trichins A (**19**), which was identical to those of kurubaschic acid angelate and trichins B (**20**) and was derived from trichins A that isolated from leaves and roots of *Trichilia monadelpha* (Thonn.) J. De Wild. [50]. 

Previous studies have isolated five germacrane-type sesquiterpenoids from plants in the Meliaceae family. Two germacranes from the genus *Guarea*, *trans*-bicyclogermacradiene (**21**) and *cis*-bicyclogermacradiene (**22**), were isolated from the stem bark of *Guarea macrophylla* [51]. Another two newly discovered oxygenated germacrane skeletons from the genus *Trichilia*, germacra-10(14)-en-9,11,15-triol (**23**) and germacra-3,10(14)-dien-9,11-diol-4-carbaldehyde (**24**) have been isolated from the stems of *Trichilia claussenii* C.DC., and their structure was determined by various analytical methods such as NMR, IR, and ESI-MS [47]. The last germacrane-type discovered from the genus *Lansium*, germacrene-D (**25**), was isolated from the fruit peel of *Lansium domesticum* Correa [52]. 

Furthermore, minor types of monocyclic sesquiterpenoids were obtained from various genera in the Meliaceae family, such as three compounds of ionone sesquiterpenoids 3*R*,6*S*-3-hydroxy-*α*-ionone (**26**) from the stem bark of *Dysoxylum parasiticum* (Osbek). Kosterm, vomifoliol (**27**) from the stems of *Aphanamixis grandifolia*, and (3*R*,6*R*,7*E*)-3-hydroxy-*α*-ionone (**28**) from the twigs and leaves of *Aglaia lawii* [44,53,54]. A nor-sesquiterpenoid-type with additional hydroxy groups at positions 9 and 10 in the side chain (C_13_H_20_O_3_), 9,10-dyhydroxy-4,7-megastigmadiene-3-one (**29**) was isolated from the leaves of *Aglaia gracilis* A.C.Sm. [23]. Moreover, a modification of guaiane-type sesquiterpenoid has yielded a *seco*-guaiane 7-epi-10-hydroxychabrol-1(2)-en-4,5-dione A (**30**) from the leaves of *Trichilia maynasiana* C.DC. [55]. Fadhilah et al. also reported a sesquiterpenoid aldehyde 2-ethyl,3-(1′-hydroxy-2′-menthene) propenal (**31**) from the fruit peel of *Lansium domesticum* Correa [56]. Furthermore, schensianol A (**32**) was identified from the leaves of *Dysoxylum oliganthum* C.Y.Wu [42]. 

#### 3.2.3. Bicyclic Sesquiterpenoids 

**Figure 6:**
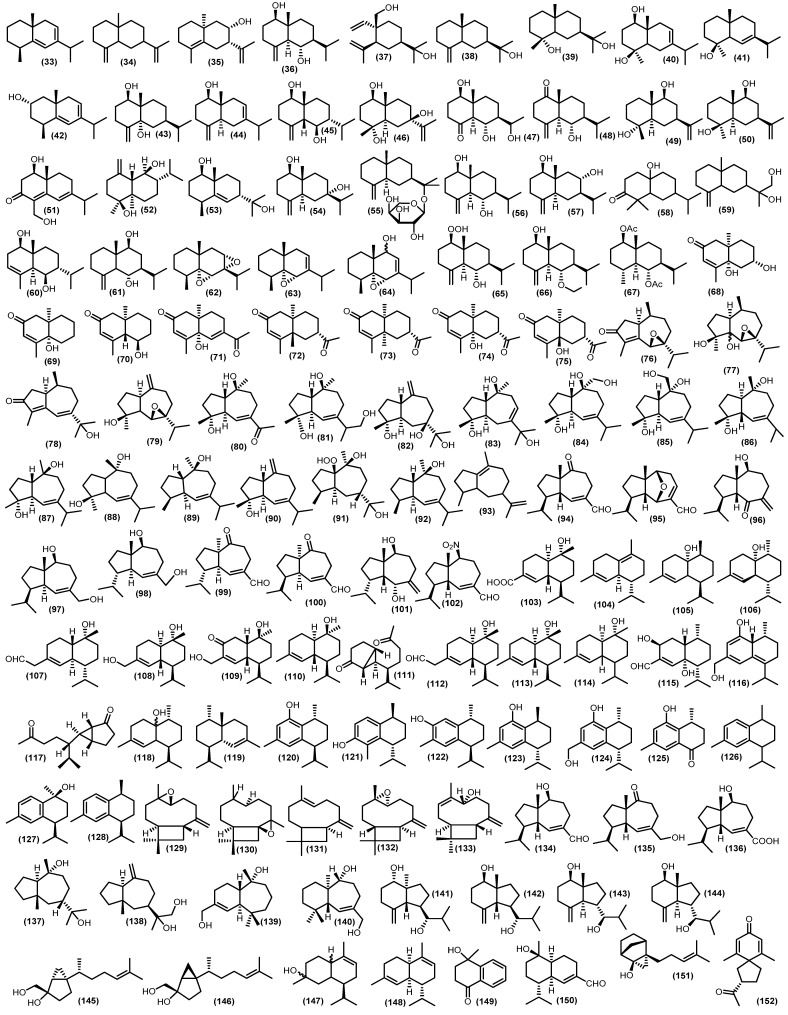
Isolated bicyclic sesquiterpenoids.

Approximately 120 bicyclic sesquiterpenoid compounds have been identified from the Meliaceae family with various spectroscopic methods (Figure 6). The larger type of bicyclic framework is eudesmane-type, with 43 compounds. Non-oxygenated eudesmane sesquiterpenoids are hardly isolated in the Meliaceae family. There are three compounds such as eudesma-5,7-diene (**33**), *β*-selinene (**34**), and (+)-eudesma-4,11-dien (**35**) have been isolated from leaves of *Guarea guidnonia* (L.) Sleumer [57,58]. Oxygenated eudesmanes form the major class of sesquiterpenoids in the Meliaceae family, including alcohol, epoxides, peroxides, ketones, ethers, and *O*-glucose. The different functional groups are important to give rise to distinct bioactivities of sesquiterpenoids. The hydroxyl-substitution eudesmanes have been reported, 4(15)-eudesmen-1*β*,6*α*-diol (**36**) is a known compound found in several species in the Meliaceae family. It has been found in the various parts of plants such as stembark, stems, seeds, twigs, leaves, from *Aglaia minahassae* Koord., *Cipadessa cinerascens* (Pellegr.) Hand.-Mazz., *Cipadessa baccifera* (Roth) Miq., *Guarea guidonia*, *Amoora tsangii* (Merr.) X.M.Chen, *Lansium domesticum Correa*, *Dysoxylum densiflorum* (Blume) Miq., *Dysoxylum parasiticum*, *Turraeanthus africanus* (Wele. Ex C.DC.) Pellegr., and *Aglaia grandis*, respectively [3,6,59,60,61,62,63,64,65,66]. Another three compounds of this type, 14-hydroxyelemol (**37**), *β*-eudesmol (**38**), and cryptomeridiol (**39**), have been obtained from the stems of *Trichilia claussenii*; their structure was elucidated by the basis of spectroscopic analysis [47]. A compound known as cryptomeridiol (**39**) is also found in *Cipadessa bacifera* and *Cedrela fissilis* Vell. [61,67]. Furthermore, oplodiol (**40**) was isolated from the pericarp of *Toona sinensis* (A.Juss.) M. Roem. [68,69]. From the leaves and seeds of *Guarea guidonia* were obtained eudesm-6-en-4*β*-ol (**41**), (2S*)-eudesma-5,7-dien-2-ol (**42**), eudesm-4(15)-ene-1*β*,5*α*-diol (**43**), eudesm-4(15),7-dien-1*β*-ol (**44**), and 5-epi-eudesm-4(15)-ene-1*β*,6*β*-diol (**45**), and eudesm-4(15),7-dien-1*β*-ol (**44**) was also found in the stembark of *Lansium domesticum* [57,62,64]. Moreover, 5-epi-eudesm-4(15)-ene-1*β*,6*β*-diol (**45**) was also found in the stembark of *Aglaia elaeagnoidea* [70]. Moreover, from the twigs of *Aglaia odorata* Lour. var. macrophyllina, 1*β*,4*α*,7*β*-trihydroxy-14*β*-methyl-eudesman-11(12)-ene (**46**) and 1*β*,6*α*-dihydroxy-10*β*-methyl-5*α*H,7*α*-eudesm-4-one (**47**) were investigated [71]. In addition, 6*α*-hydroxy-4(15)-eudesmen-1-one (**48**) has been reported from twigs, leaves, and stembark of *Aglaia lawii*, *Aglaia elaeagnoidea*, and *Chisocheton lasiocarpus* (Miq) Valeton [44,70,72]. Furthermore, from the stems and leaves of *Aphanamixis grandifolia*, voleneol (**49**) has been reported [54]. The eudesm-11(13)-en-4*β*,9*β*-diol (**50**) has been found in the twigs and leaves of *Aglaia lawii* [44]. From the genus *Aglaia* a new eudesmane-type sesquiterpenoid 4,6-diene-1*β*,15-dihydroxyeudesma-3-one (**51**) was also discovered that gained from the stembark of *Aglaia foveolate* Pannell [73]. The eudesmane-type also reported another three compounds, including polydactin B (**52**), 1*β*,11-dihydroxy-5-eudesmene (**53**) from the leaves of *Dysoxylum excelsum* Blume, and 4(15)-eudesmene-1*β*,7*α*-diol (**54**) from the leaves and barks *Dysoxylum densiflorum* [42,74]. Djemgou et al. found an eudesmane-type sesquiterpenoid modified by an arabinose-sugar skeleton that is (+)-eudesmanol-*O*-L-arabinoside (**55**) from the seed of the rare Meliaceae species *Turraeanthus africanus* [75]. Additionally, two eudesmane-type have been reported from the twigs of *Chisocheton cumingianus* subsp. Balansae (C.DC.) Mabb., 1*β*,6*α*-dihydroxyeudesm-4(14)-ena (**56**) and 1*β*,8*α*-dihydroxy-eudesm-4(14)-ena (**57**) [76]. A new eudesmane sesquiterpenoid 10*β*-hydroxy-4*α*,4*β*-dimethyl-5*α*H,7*α*H-eudesm-3-one (**58**) was isolated for the stem bark of *Dysoxylum parasiticum*, and the structure was determined by detailed analysis of spectroscopic data, including MS, IR, 1D, and 2D NMR, as well as through comparison with data of known analogs [77]. The kutdiol (**59**) was obtained from the stems of *Trichilia quadrijuga* Kunth and isolated by the Droplet Counter-Current Chromatography (DCCC) technique [78]. In addition, 1*β*,6*β*-dihydroxy-7-epi-eudesm-3-ene (**60**) was elucidated from the barks of *Melia azedarach*, and 6*α*,9*β*-dihydroxyeudesma-4(15)-ene (**61**) was found in the pericarp of *Lansium domesticum* [79,80]. The eudesman epoxides were found on the leaves of *Guarea guidonia* such as, 5*α*,6*α*,7*α*,8*α*-diepoxy-eudesmane (**62**), 5*α*,6*α*-epoxy-eudesm-7-ene (**63**) and 5*α*,6*α*-epoxy-eudesm-7-en-9-ol (**64**) [57,58]. A novel peroxide-substituted eudesmane 1*β*-hydroperoxy-6*α*-hydroxy-eudesm-4(15)-ene (**65**) has been found in the branches of *Cipadessa cinerascens* [60]. Recently isolated eudesmane ether 6*α*-ethoxyeudesm-4(15)-en-1*β*-ol (**66**) from the seeds of *Guarea guidonia* [62]. Moreover, the eudesmane esther-type sesquiterpenoid voleneol diacetate (**67**) was isolated from the stem barks of *Lepidotrichilia volensii* Leroy [81]. Moreover, from the fruits of *Dysoxylum densiflorum*, three 11, 12, 13 trisnoreudesmanes dysoxydenones M-O (**68**–**70**) were gained, and three 13-noreudesmanes dysoxydenones P-R (**71**–**73**) were identified. Their absolute configurations were determined by a combination of single crystal X-ray diffraction, CD exciton chirality method, and ECD calculations. Another two known analogs, noreudesmanes types dysoxydenone G (**74**) and dysoxydenone H (**75**), were isolated from the same samples as further oxidation of prenyleudesmanes [82]. 

Guaiane-type sesquiterpenoids often occur in oxygenated forms, such as guaiane alcohol, guaiane acid, guaiane ketone, and guaianolide. There were nineteen isolated guaiane-type sesquiterpenoids spread across several species of Meliaceae. From the stembark of *Amoora rohituka* (Roxb.) Wight and Arn., four oxygenated guaiane-type sesquiterpenoids were isolated, such as 6*β*,7*β*-epoxyguai-4-en-3-one (**76**), 6*β*,7*β*-epoxy-4*β*,5-dihydroxyguaiane (**77**), sootepdienone (**78**), and orientalol C (**79**) [83]. Moreover, 4*α*,10*β*-dihydroxy-1*β*H,5*α*H-guai-6(7)-en-11-one (**80**), 1*α*,6*β*,12-trihydroxy-1*β*H,5*α*H,11H-guai-6(7)-ene (**81**), 4*α*,7*β*,11-trihydroxy-1*β*H,5*α*H-guai-10(14)-ene (**82**), 4*α*,10*α*,11-trihydroxy-1*β*H,5*β*H-guai-7(8)-ene (**83**), orientalol A (**84**), and orientalol B (**85**) were elucidated from the twigs of *Aglaia odorata* var. macrophyllina with various spectroscopic analysis method [71]. Another compound from the genus *Amoora* 6-guaiene-4*α*,10*α*-diol (**86**) was isolated from the bark of *Amoora yunnanensis* species [84]. A known compound, guaianediol (**87**), was gained from various parts of the plant, such as twigs, stem barks, stems, and leaves of various species, including *Aglaia odorata*, *Cipadessa baccifera*, *Dysoxylum parasiticum*, *Dysoxylum excelsum*, *Aglaia lawii*, and *Chisocheton lasiocarpus* [42,44,61,71,72,77]. Another known guaiane-type is alismoxide (**88**) from the leaves, pericarp, and twigs of several species such as *Guarea kunthiana* A. Juss, *Toona sinensis*, *Chisocheton cumingianus*, and *Dysoxylum densiflorum* [65,69,76,85]. The compound guai-6-en-10*β*-ol (**89**) was found in the leaves and stembark of *Guarea guidonia* and *Guarea macrophylla*, along with alismol (**90**) from the leaves of *Guarea kunthiana* [57,85,86]. The other guaiane-oxygenated compounds are found in the stembark of *Dysoxylum parasiticum*, and a new 10*β*,11-dihydroxy-1*β*-hydroperoxide-4*α*H,5*α*H,7*β*H-guaiane (**91**) and a known compound (1S*,4S*,5R*,10S*)-guai-6-ene-10*β*-ol (**92**) were determined by completed spectroscopic analysis [3,77,87]. Moreover, the wood of *Lansium anamalayanum* provided the new guaiane-type chigdamarene (**93**) and was identified by IR and NMR spectra analysis for the structure [39].

There were nine compounds with isodaucane-type sesquiterpenoids obtained from several Meliaceae species. The isodaucane compound 2-oxo-isodauc-3-en-15-al (**94**) has been found in barks, fruits, twigs, and leaves of four species, including *Aglaia foveolate* Pannel, *Aglaia perviridis* Hiern, *Aglaia lawii*, and *Walsura robusta* Roxb. [44,88,89,90]. Two new isodaucane-type sesquiterpenoids, amouanglienoid A (**95**) and amouanglienoid B (**96**), were obtained from the twigs and leaves of *Aglaia lawii*; moreover, the structure was confirmed by X-ray crystallographic studies as well as comparison by experimental and calculated ECD spectra data [44]. Huang et al. reported four compounds, namely isodauc-6-ene-10*β*,14-diol (**97**), 4-epi-isodauc-6-ene-10*β*,14-diol (**98**), sinulin A (**99**), and 10-oxo-isodauc-3-en-15-al (**100**) from the twigs and leaves of *Aglaia elaeagnoidea* [91]. Compounds (**97**–**98**) were also found in the leaves of *Dysoxylum excelsum* [42]. Furthermore, 4-epi-6*α*,10*β*-dihydroxy-artabotrol (**101**) isolated from leaves *Dysoxylum excelsum* [42], as well as the nitro-substituent isodaucane-type sesquiterpenoid 10*β*-nitro-isodauc-3-en-15-al (**102**), was determined from the leaves of *Walsura robusta* [90].

A chemical study of an extract from the twigs and leaves of *Dysoxylum densiflorum* afforded one new cadinene-sesquiterpenoid derivative dysodensiol D (**103**) [65]. Four years later, Liu et al. also found dysodensiol D (**103**) in the leaves of *Dysoxylum excelsum* [42]. Lago et al. reported three compounds, namely *δ*-cadinene (**104**), *trans*-cubenol (**105**), and *cis*-cubenol (**106**), from the stembark of *Guarea macrophylla* [51]. Other cadinane derivatives from *Aglaia* genus, such as 15-oxo-T-cadinol (**107**), 15-hydroxy-*α*-cadinol (**108**), 3-oxo-15-hydroxy-T-muurolol (**109**), and (+)-T-cadinol (**110**), were investigated from the stembarks, twigs, and leaves, including *Aglaia foveolata* and *Aglaia elaeagnoidea* [73,88,91]. Known compound 15-hydroxy-*α*-cadinol (**108),** along with chromolaevane dione (**111),** a cadinane ketone-type sesquiterpenoid, has been found in the twigs and leaves of *Dysoxylum densiflorum* [65]. The aldehyde cadinane-type sesquiterpenoid 10*α*-hydroxycadin-4-en-15-al (**112**) was investigated from the stems of *Aphanamixis grandifolia* [54]. Moreover, the oxygenated cadinene-type sesquiterpenoid 10-hydroxy-15-oxo-*α*-cadinol (**113**) was found in the twigs and leaves of *Amoora tsangii* [63]. Another compound of this type, *α*-cadinol (**114**), was obtained from the stembark of *Dysoxylum parasiticum* [77]. Moreover, two new undescribed cadinanes, deriving from *α*-cadinol with minor modification dysotican A (**115**) and dysotican B (**116**), were determined for the absolute configuration by ECD spectra technique from stembark of *Dysoxylum parasiticum* [3]. Furthermore, the norsesquiterpenoid cadinane-type saniculamoid (**117**) was investigated from stem barks of *Aglaia grandis* Korth. [6]. Moreover, the cadinane–alcohol skeleton modification cubenol (**118**) and epi-cubenol (**119**) were investigated from two species, including *Cedrela odorata* L. and *Cedrela toona* Roxb [31,92]. 

A study conducted by Nishizawa et al. showed that (+)-8-hydroxycalamenene (**120**) has been elucidated from the leaves of *Dysoxylum acutangulum* Miq. and *Dysoxylum excelsum*. Furthermore, fifteen years later, Mulholland et al. also reported the same compound from the wood of *Dysoxylum shciffneri* [4,40]. Moreover, the same type of these compounds was also found in the stem barks of *Dysoxylum parasiticum*, *Dysoxylum densiflorum*, and *Dysoxylum shciffneri*, namely, dysoxyphenol (**121**), (+)-7-hydroxycalamenene (**122**), (7*R*,10*S*)-2-hydroxycalamenene (**123**), 2,15-dihydroxycalamenene (**124**), and schiffnerone B (**125**) [3,40,53,93]. A known compound, calamenene (**126**), was also reported from various species, including *Cedrela odorata* and *Cedrela toona* [31,92]. In addition, calamenene-10*β*-ol (**127**) was isolated from the bark of *Entandrophragma cylindricum* (Sprague) Sprague along with cis-calamenene (**128**) reported from stem barks of *Guarea macrophylla* [86,94]. 

Additionally, minor types of bicyclic sesquiterpenoids were found in various genera in the Meliaceae family. There are five caryophyllene-type sesquiterpenoid namely, *β*-caryophyllen oxide (**129**), *β*-caryophyllene-8*R*,9*R*-oxide (**130**), *β*-caryophyllene (**131**), caryophyllene oxide (**132**), and caryophyllenol-II (**133**) were obtained from various parts of plant such as stem barks, barks, stems, and wood of several species, including *Aglaia harmsiana* Perkins, *Aglaia leucophylla* King, *Guarea macrophylla*, *Munronia pinata* Harms, *Turraea brownie* C.DC., *Aglaia foveolata*, *Sandoricum koetjape* Merr., *Guarea guidonia*, *Munronia pinata*, and *Aglaia simplicifolia* (Bedd.) Harms [46,57,87,88,95,96,97,98,99]. The hydro-azulene type sesquiterpenoids, namely aphanamol II (**134**) and aphanamol I (**135**), are widely distributed in various species of Meliaceae plants, including *Aphanamixis grandifolia*, *Cipadessa baccifera*, *Lansium domesticum*, *Dysoxylum excelsum*, *Aglaia lawii*, *Dysoxylum densiflorum*, and *Turraeanthus africanus* [42,44,52,61,65,75,100]. Another compound of this type, a new sesquiterpenoid dysodensiol E (**136**), was obtained from the twigs and leaves of *Dysoxylum densiflorum* [65]. Furthermore, two guaiane-type derivatives, which are *pseudo*-guaiane namely ambrosanoli-10,11-diol (**137**) and ambrosanoli-10(14)-en-11,12-diol (**138**), have been identified as constituents of *Trichilia casarettii* [78,101]. Himachalane derivatives are the main sesquiterpenoid component of the stems of *Cipadessa baccifera*, namely bacciferins A (**139**) and bacciferins B (**140**) [61]. The oppositane-type sesquiterpenoids have been isolated from three species, *Lansium domesticum*, *Guarea guidonia*, and *Dysoxylum excelsum*, namely octahydro-4-hydroxy-3α-methyl-7-methylene-α-(1-methylethyl)-1H-indene-1-methanol (**141**), (7R*)-5-epi-opposit-4(15)-ene-1*β*,7-diol (**142**), (7R*)-opposit-4(15)-ene-1*β*,7-diol (**143**), and (7R*)-opposit-4(15)-ene-1*β*,7-diol (**144**) [42,62,64]. The stems of *Dysoxylum oliganthum* contain two novel sabinene-type sesquiterpenoids, (6*R*,7*S*,11*R*,10*S*)-15-hydroxy-sesquisabinene hydrate (**145**) and (6*R*,7*R*,11*S*,10*S*)-15-hydroxy-sesquisabinene hydrate (**146**), and the absolute configuration was determined by CD measurement [42]. *α*-muurolene (**147**), a murolene-type sesquiterpenoids, was gained from the leaves of *Aglaia silvestris* (M. Roem.) Merr. , and the derivate of that compound T-muurolol (**148**) was produced by two species *Cedrela odorata* and *Cedrela toona* [31,92,102]. In addition, hydroxylated tetralone-type sesquiterpenoids 4-hydroxy-4,7-dimethyl-*α*-tetralone (**149**) are also found in leaves and stem barks of *Dysoxylum parasiticum* and *Cipadessa boiviniana* [41,103]. Furthermore, from the twigs and leaves of *Aglaia lawii*, bicyclic sesquiterpenoid canangaterpene III (**150**) was gained [44]. 2,3-dimethyl-3-(4-methyl-3-pentenyl)-2-norbornanol (**151**) with bicyclic skeleton sesquiterpenoids also found from the fruit of *Dysoxylum spectabile* (G. Forst.) Hook. Fil. [104]. In addition, the spirovetivane-type sesquiterpenoid dysoxydenone S (**152**) was produced from the fruit of *Dysoxylum densiflorum* [82]. 

#### 3.2.4. Tricyclic and Other Sesquiterpenoids

**Figure 7:**
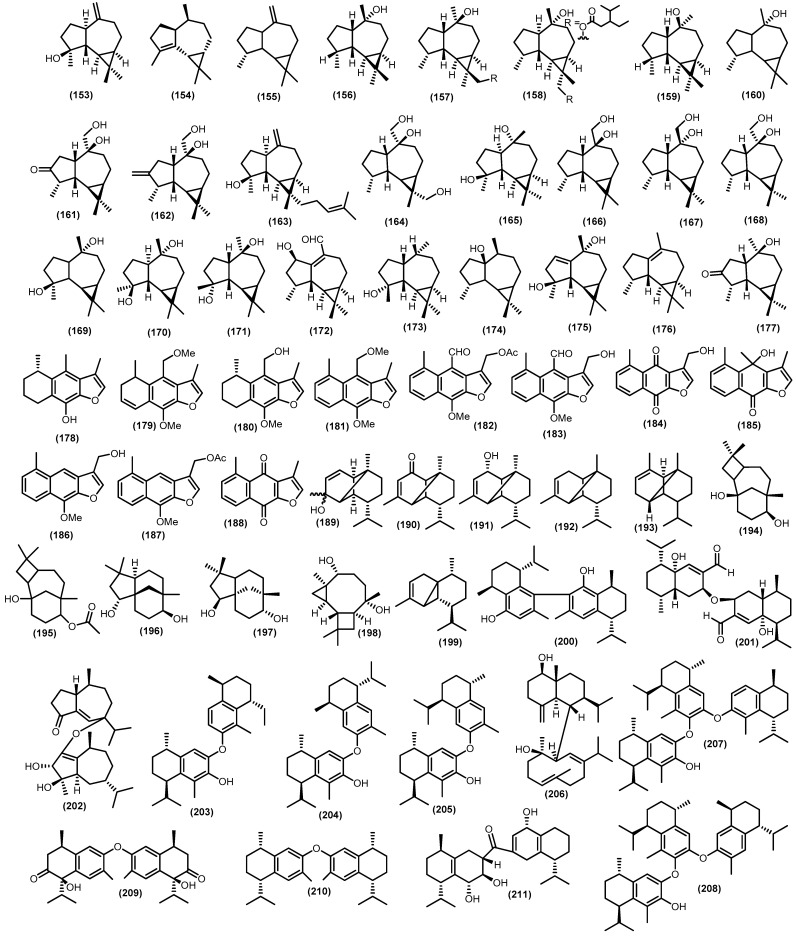
Isolated tricyclic, dimeric, trimeric sesquiterpenoids.

Aromadendrane comes in an abundance of tricyclic sesquiterpenoids with 26 compounds (Figure 7). Structurally, aromadendrane is characterized by a dimethyl cyclopropane ring fused to a hydro-azulene skeleton. Moreover, among aromadendrane compounds which hydro-azulene skeleton is *cis*-fused is termed alloaromadendrane. The frequently aromadendrane spathulenol (**153**) was isolated for the first time from the stems of *Sandoricum koetjape* and found so far in numerous plant species [39,45,47,59,77,87,88,94,98,105,106,107,108]. The wood of *Lansium anamalayanum* provided the *α*-gurjunene (**154**), which was identified by IR and HMR spectra analysis for the structure [39]. A number of other aromadendrane hydrocarbon aromadendrene (**155**) has been gained from the stem barks and barks from two different tree species such as *Guarea macrophylla* and *Sandoricum koetjape* [51,98]. Another type of hydroazulene skeleton *cis*-fused, namely ledol (**156**), was found in numerous plant species such as *Aglaia foveolata*, *Etandrophragma cylindricum*, and *Guarea macrophylla* [45,88,94]. Nugroho et al. reported two new aromadendrane, dysosesquiflorin A (**157**) and the *α* position for 3-isopropylpentanoate substituent dysosesquiflorin B (**158**), which were determined based on analysis of the 1D and 2D NMR data (HSQC, ^1^H-^1^H COSY, and HMBC) along with two known compounds, viridiflorol (**159**) and (−)-globulol (**160**) [109]. Oxygenated aromadendranes are widespread in a new compound dysodensiol F (**161**), along with allo-aromadendrene-10*β*,14-diol (**162**), 4*β*-hydroxy-15-(3-methyl-2-butenyl)-aromadendra-10(12)-ene (**163**), and allo-aromadendrane-10*β*,13,14-triol (**164**), and were identified from the twigs, leaves, and barks of *Dysoxylum densiflorum* [65,74]. Moreover, allo-aromadendrane-10*β*,13,14-triol (**164**) has also gained from wood and leaves *Chisocheton Penduliflorus* Planch. [110]. Furthermore, the same type of these compounds also found in leaves, stem barks, twigs from numerous species, namely 4*β*,10*α*-dihydroxyaromadendrane (**165**), (+)-10*β*,14-dihydroxy-allo-aromadendrane (**166**), allo-aromadendrane-10*α*,14-diol (**167**), allo-aromadendrane-10*β*,14-diol (**168**), (−)-4*α*,7*α*-aromadendranediol (**169**), aromadendrane-4*β*,10*α*-diol (**170**), and alloaromadendrane-4*α*,10*β*-diol (**171**) [3,6,42,44,72,87,91,99,110,111].

A new aromadendrane aldehyde-type sesquiterpenoid was obtained from the twigs of *Turraea pubescens* Hell., namely turranin F (**172**), which has similar structure to(4*R*,5*S*,6*R*,7*R*,11*S*)-12-hydroxy-1(10)-aromadendren-14-al from the genus *Apocynaceae* [112,113]. The spathulenol derivative 1,1,4,7-tetramethyldecahydro-1H-cyclopropa[e]azulen-7-ol (**173**) was also found in the stem barks of *Chisocheton pentandrus* (Blanco) Merr [114]. Other plants species that provide oxygenated aromadendranes, such as palustrol (**174**), lochmolin F (**175**), virindiflorene (**176**), and 3-oxo-10-alloaromadendranol (**177**), were determined from *Guarea macrophylla*, *Guarea guidonia*, and *Trichilia maynasiana* [46,48,55,57,87].

Additionally, 11 tricyclic-sesquiterpenoids furanoeremophilane were investigated from the *Trichilia* genus. The sesquiterpenoid furanoeremophilane in *Trichilia cuneata* Radlk. species is present as a characteristic constituent of secondary metabolites. The eremophilane family is a large, structurally diverse group of sesquiterpenoids characterized by a decalin skeleton in which a methyl migration has taken place to produce a non-isoprenoid substituent pattern. A derivate of this group, the furanoeremophilanes, bears a furan fused to the decalin core, which, in several cases, appears in oxidized form as a butenolide. A study conducted by Doe et al. gained a new furanoeremophilanes-type related to sesquiterpenoids cacalol (**178**), which is isolated from the same samples, namely (+)-14-methoxy-1,2-dehydrocacalol methyl ether (**179**) [115]. Moreover, from the same samples, six known compounds were also reported, such as (±)-14-hydroxycacalol methylether (**180**), 14-methoxydehydrocacalohastine (**181**), maturin acetate (**182**), maturin (**183**), maturone (**184**), and cacalonol (**185**). The first total synthesis of (**179**) and two related types (**180**–**181**) was also achieved via stepwise regioselective dehydrogenation of ring C [115]. Moreover, two novel eremophilane cacalols were also discovered, 13-hydroxy-14-nordehydrocacalohastine (**186**) and 13-acetoxy-14-nordehydrocacalohastine (**187**), along with maturinone (**188**), which were reported from the stembark of an endemic medicinal plant complex in Mexico which is *Trichilia cuneata* [116]. The total synthesis of these two new compounds (**186**–**187**) was also reported via a palladium-mediated three-component coupling reaction [116]. 

Other minor tricyclic sesquiterpenoids were also investigated. The copaene derivative copa-2-en-4-ol (**189**), copa-3-en-2*α*-ol (**190**), and mustakon (**191**) were gained by HPLC separation method from the bark of *Entandrophragma cylindricum* [94]. Moreover, the same type of tricyclic sesquiterpenoids copaene (**192**) and *α*-copaene (**193**) were gained from various species [31,52,86]. Two known compounds of senecrassidiol (**194**) come from various species, such as *Aglaia simplicifolia*, *Aglaia harmsiana*, and *Munronia pinata*; additionally, the derivate senecrassidiol acetate (**195**) was elucidated from *Aglaia elaeagnoidea* [59,95,117,118]. Two species of genus *Aglaia*, *Aglaia elaeagnoidea* and *Aglaia simplifolia*, produced (−)-clovane-2,9-diol (**196**), which are clovane-type sesquiterpenoids, along with clovanediol (**197**) from other species, namely *Dysoxylum densiflorum* [65,91,99]. Moreover, other minor tricyclic compounds from the Meliaceae family were tricyclohumuladiol (**198**) and *α*-cubebene (**199**) from leaves and stem barks of *Dysoxylum oligantum* C.Y.Wu and *Guarea macrophylla* [42,45]. 

Biogenetically derived from coupling two sesquiterpenoids (either identical or different), dimeric sesquiterpenoids are potential biologically active molecules and have received considerable attention in recent years for their distinctive structures and biological properties. They have a composition of at least 30 carbons, generated from sesquiterpenoids of a variety of structural types, and show variations in the connecting patterns of the two identical (for homo-dimeric sesquiterpenoids) or different (for hetero-dimeric sesquiterpenoids) sesquiterpenoid units, which presents a significant challenge in elucidating dimeric sesquiterpenoids structures and synthetic constructions. *The dysoxylum* plant genus possesses attractive dimeric as well as trimeric sesquiterpenoids. Nishizawa et al. reported unsymmetrical dimeric calamenene, namely bicalamenene (**200**), from the dried peel of the Meliaceous plant *Dysoxylum alliaceum* (Blume) Blume [119]. The structure of that compound was elucidated by spectra analysis, and the absolute structure was established by analog synthesis compound; it was reported that the two compounds were confirmed for the ^13^C NMR, IR, and UV, and only the chemical shift of aromatic methyl protons are distinguishable. Additionally, two identical dimeric sesquiterpenoids were gained from the stem barks of *Dysoxylum parasiticum*, namely dysotican C (**201**) and dysotican D (**202**), and the absolute configuration was confirmed by ECD and NMR calculation data. The plausible route biosynthesis of dysotican C comes from the epimerization of (**114**), which is also found in the same samples, while dysotican D comes from the epimerization process of (**87**) [3,77]. Moreover, bidysoxyphenol A (**203**), bidysoxyphenol B (**204**), and bidysoxyphenol C (**205**) were also gained in the same samples [53]. Dysotican E (**206**), a hetero-dimeric sesquiterpenoid, was also found in the same samples of *Dysoxylum parasiticum* [3]. Two new trimeric sesquiterpenoids were produced by stem barks of *Dysoxylum parasiticum*, namely tridysoxyphenol A (**207**) and tridysoxyphenol B (**208**), while those trimeric compounds were formed by radical addition reaction of monomer dysoxyphenol (**121**) to obtain (**207**), as well as the monomer (+)-7-hydroxycalamenene (**122**) with bidysoxyphenol A (**203**), to produce (**208**) [103]. Additionally, two unprecedented homo-dimeric sesquiterpenoids, dysotican G (**209**) and dysotican H (**210**), which linked through O-ether linkage and an asymmetrical true-dimeric cadinane via ketonic bridge dysotican F (**211**), were isolated from the stembark of *Dysoxylum parasiticum*. Their structure was determined by spectroscopic and quantum chemical calculations of ^13^C NMR using the GIAO method and ECD using the TDDFT method [120]. 

### 3.3. Volatile Sesquiterpenoids

This section focused on sesquiterpenoids composition, which is determined by the hydrodistillation technique followed by GC-MS analysis as volatile compounds. The sesquiterpenoids structure was identified by mass-spectral data, retention indices (on comparison of their retention times to *n*-alkanes (C8-C40), and by computer matching with various databases. As lipophilic molecules with moderate vapor pressures, volatile sesquiterpenoids convey information over distances well, and because of their wide structural variety, they also allow messages to be very specific. Additionally, volatile sesquiterpenes usually produce several compounds often related to each other. Volatile sesquiterpenoids in the Meliaceae family have almost exclusively been reported from three genera, including *Trichilia*, *Toona*, and *Cedrela*. Many species of the Meliaceae family have been reported to produce volatile sesquiterpenoids (Table 1).

**Figure 8:**
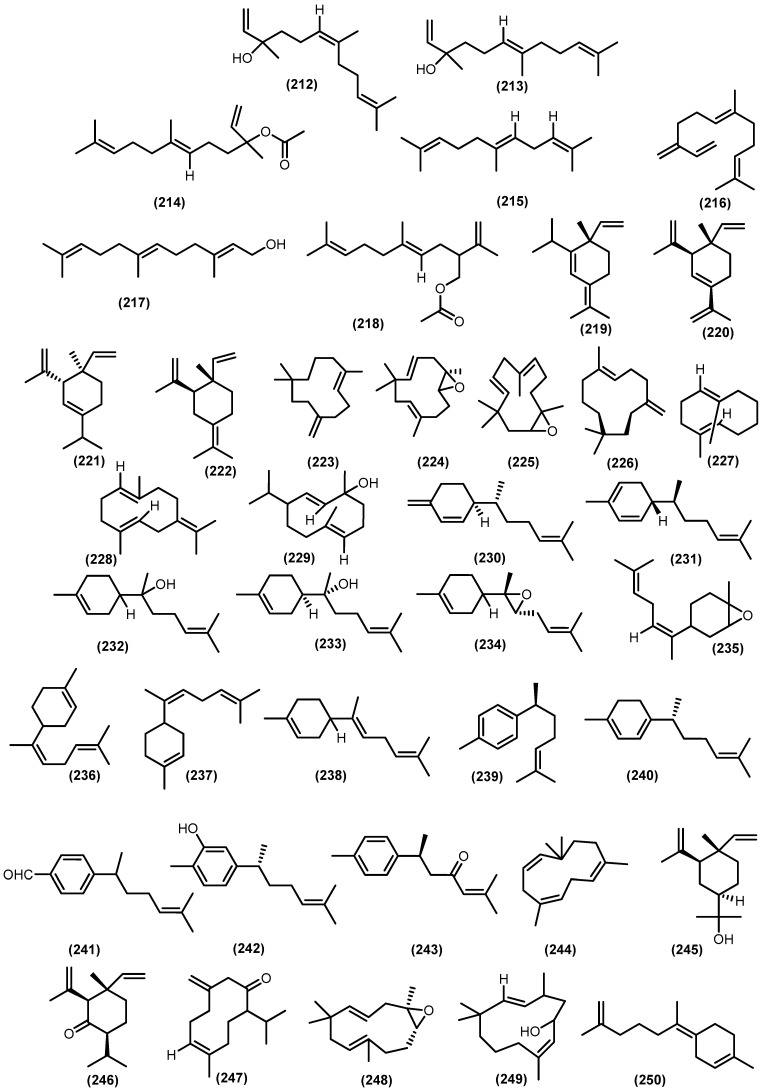
Acyclic and monocyclic volatile sesquiterpenoids from Meliaceae family.

**Figure 9:**
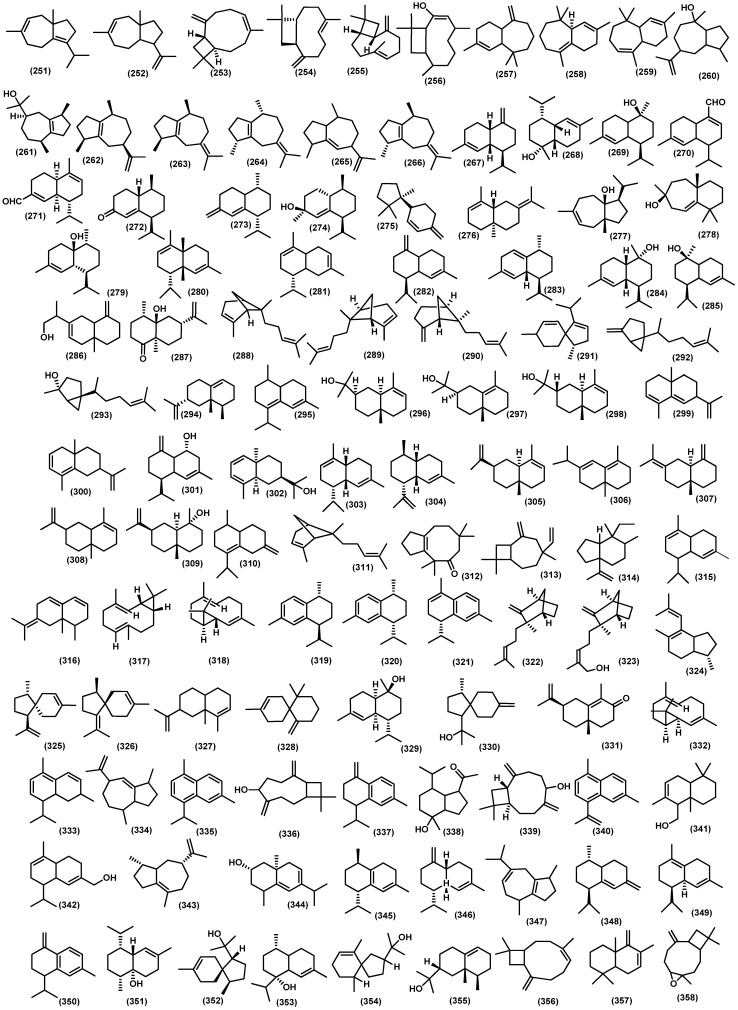
Bicyclic volatile sesquiterpenoids from Meliaceae family.

The bicyclic sesquiterpenoids *γ*-himachalene (**259**) was the major constituent from the roots of *Naregamia alata* Wight and Arn., along with 33 sesquiterpenoids. The minor compounds were identified as one acyclic sesquiterpenoids nerolidyl acetate (**214**) and seven monocyclic skeleton *γ*-elemen (**222**), *α*-humulene (**13**), 9-epi-(*E*)-caryophyllene (**226**)**,** germacrene B (**228**), germacrene D (**25**), *β*-sesquiphellandrene (**230**), and bisabolol (**233**) (Figure 8). Sixteen bicyclic sesquiterpenoids include daucene (**251**), *β*-caryophyllene (**131**), caryophyllene oxide (**132**), cis-*β*-guaiene (**264**), *α*-muurolene (**147**), 14-oxy-*α*-muurolene (**270**), *β*-cuprenene (**275**), selina-3,7-(11)-diene (**276**), carotol (**277**), widdrol (**278**), 1-epi-cubenol (**119**), cubenol (**118**), vetiselinenol (**286**), and corymbolone (**287**) (Figure 9). 

**Figure 10:**
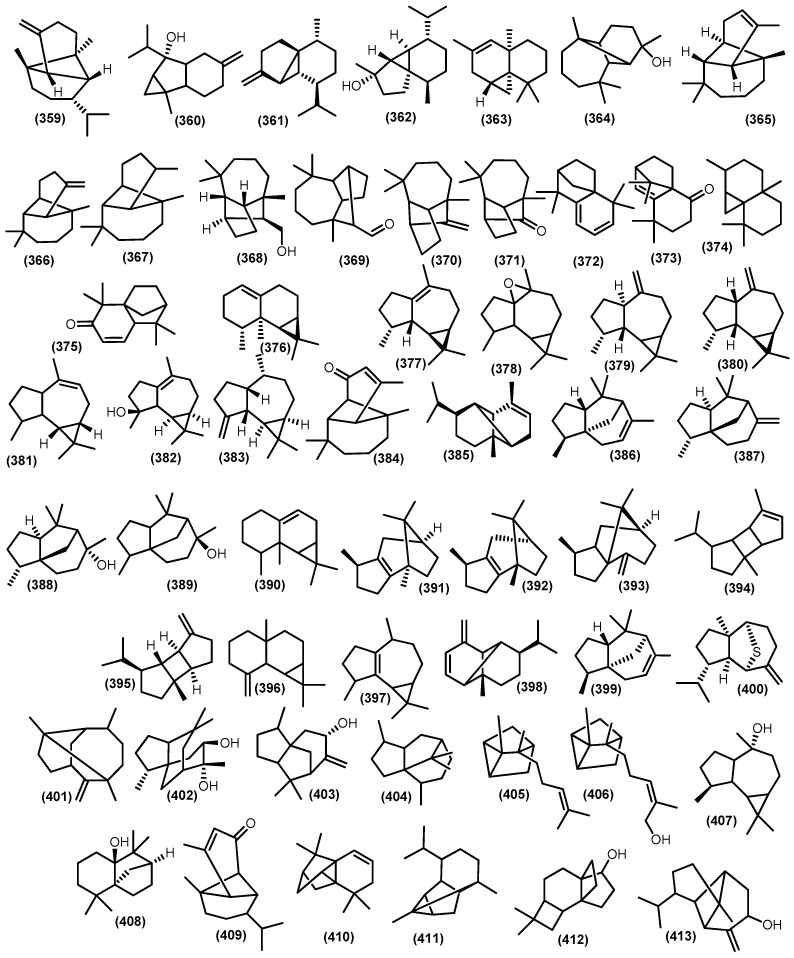
Tricyclic and tetracylic volatile sesquiterpenoids from Meliaceae family.

Moreover, the tricyclic skeleton was also found on the same plants, including *α*-copaene (**193**), *β*-cubebene (**361**), longipinane (E) (**367**), *cis*-thujopsene (**363**), longifolol (**368**), longifolenaldehyde (**369**), and 2,4*α*,8,8-tetramethyl decahydro cyclopropa (d) naphthalene (**374**) [121] (Figure 10). The presence of tricyclic and tetracyclic sesquiterpenoids hydrocarbon from Meliaceae essential oil appears to be a minor component compared to the bicyclic constituent. The substantial differences in sesquiterpenoids composition in the Meliaceae essential oil come from climate fluctuations [122,123]. Moreover, from the whole part of *Naregamia alata*, nineteen volatile sesquiterpenoids, with caryophyllene oxide (**132**) as the major compound, were identified. 

In addition, the minor composition of the essential oil *Naregamia alata* plants was examined, including the sesquiterpenoids ar-curcumene-15-al (**241**) and xanthorrhizol (**242**); the bicyclic skeleton daucene (**251**), isodaucene (**252**), *β*-caryophyllene (**131**), caryophyllene oxide (**132**), *cis*-14-nor-Muurol-5-en-4-one (**272**), carotol (**277**), widdrol (**278**), *δ*-cadinol (**284**), *α*-*trans*-bergamotene (**3**), *α*-acoradiene (**291**), and 7-epi-*α*-eudesmol (**298**); and other sesquiterpenoids skeleton *β*-cubebene (**361**), 8-oxo-neoisolongifolene (**373**), alloaromadendrene (**380**), and longipinanol (**364**) [124]. The acyclic volatile sesquiterpenoids were produced from leaves oil of *Toona sinensis trans*-nerodilol (**213**) [125]. Moreover, nerolidol (**212**) was produced from the leaves of *Toona sinensis* and the flowers of *Melia azedarach* L. [126,127]. Moreover, *β*-farnesene (**216**) has been detected in the essential oil of *Cedrela odorata* [128]. *α*-farnesane (**215**), a sesquiterpenoid, was gained from the leaves *Toona sinensis* and found as a minor constituent from stembark and leaves *Trichilia connaroides* (Wight and Arn.) Bentv. [125,129,130]. 

The oxygenated farnesane sesquiterpenoids farnesol (**217**) was produced by *Cipadessa baccifera* and *Trichilia connaroides* [131,132]. The acetate ester of lavandulol *cis*- sesquilavandulyl acetate (**218**) was gained from flower of *Khaya grandifoliola* C.DC. [133]. Elemene-type sesquiterpenoids *α*-elemene (**219**), *β*-elemene (**220**), *δ*-elemene (**221**), and *γ*-elemene (**222**) were gained from several genus in Meliaceae family, including *Toona sinensis*, *Chukrasia tabularis* A. Juss, *Trichilia connaroides*, *Toona sinensis*, *Cedrela fissilis*, *Cedrela mexicana* M.Roem., *Cedrela odorata*, *Cipadessa baccifera*, and *Swietenia macrophylla* G.King, [67,122,127,130,134]. A humulene-type *α*-humulene (**13**) is widely distributed in the genus Meliaceae, including *Toona*, *Naregamia*, *Azadirachta*, *Cedrela*, *Chukrasia*, *Cipadessa*, and *Swietenia*. Furthermore, a minor constituent *γ*-humulene (**223**) was produced by leaves of *Cedrela odorata* and humulene oxide (**225**) [135]. In contrast, the major components, *β*-caryophyllene (**131**) and bicyclogermacrene (**317**), were found in the leaves *Cedrela fissilis*; moreover, *β*-bisabolene (**4**) and globulol (**160**) were produced by the stem barks of *Cedrela fissilis* [136]. The other minor constituents, namely *β*-elemene (**220**), germacrene A (**227**), *α*-humulene (**13**), *β*-bisabolene (**4**), *β*-caryophyllene (**131**), caryophyllene oxide (**132**), *α*-muurolene (**147**), T-muurolol (**148**), *δ*-cadinene (**104**), T-cadinol (**110**), *α*-eudesmol (**293**), *β*-eudesmol (**38**), *β*-selinene (**34**), bicyclogermacrene (**317**), *β*-santalene (**322**), *α*-copaene (**193**), aromadendrane (**155**), spathulenol (**153**), ledol (**156**), and viridiflorol (**159**), have been identified from the leaves and stembarks of *Cedrela fissilis* [137]. From the leaves of *Toona sinensis*, germacrene-D-4-ol (**229**), *β*-caryophyllene (**131**), *α*-selinene (**305**), lepidozene (**332**), and spathulenol (**153**) have been isolated [129]. The sesquiterpene hydrocarbon bisabolane *α*-bisabolol (**232**), *cis*-*Z*-*α*-bisabolene epoxide (**231**), *trans*-*Z*-*α*-bisabolene epoxide (**235**), *cis*-*α*-bisabolene (**236**), *Z*-*α*-bisabolene (**237**), and *α*-bisabolene (**238**) were identified from *Toona sinensis* [127,138], *Turraea obtusifolia* Hochst., *Turraea floribunda* Hochst. [139], and *Swietenia macrophylla* [140]. Zingiberene (**231**) was also obtained from *Cedrela mexicana* M.Roem and *Cipadessa baccifera* [131,141].

**Table 1:** Volatile sesquiterpenoids from Meliaceae family and their bioactivities.

Species	Part of Plant	Volatile Sesquiterpenoids	Biological Activity of Essential Oil	Major Compounds	Ref.
*N. alata* Wight and Arn.	Root	**214**, **222**, **13**, **226**, **228**, **25**, **230**, **233**, **251**, **131**, **132**, **259**, **264**, **150**, **270**, **275**, **276**, **277**, **278**, **119**, **118**, **114**, **286**, **287**, **193**, **361**, **363**, **367**, **368**, **369**, **374**	Antimicrobial activity against Sa, Bs, Pv, Ec, and Kp with inhibition zone Sa (11 mm); Bs (12 mm); Pv (10 mm); Ec (13 mm); Kp (11 mm), respectively	**259**, **131**, **256**	[121]
*N. alata* Wight and Arn.	Whole plant	**224**, **241**, **242**, **251**, **252**, **131**, **132**, **272**, **277**, **278**, **284**, **285**, **3**, **291**, **293**, **296**, **298**, **358**, **361**, **364**, **373**, **380**, **381**, **384**	Not reported	**132**, **251**, **131**, **3**	[124]
*T. sinensis* (A.Juss.) M. Roem.	Leaves	**215**, **220**, **221**, **13**, **4**, **240**, **131**, **132**, **262**, **263**, **280**, **281**, **104**, **3**, **34**, **329**, **193**, **199**, **361**, **155**, **153**, **389**, **395**	Antimicrobial activity against Sa, Sp, Ec, Pa, Sf, and St with MIC values Sa (1.57 µg/mL); Sp (1.57 µg/mL); Ec (3.13 µg/mL); Pa (3.13 µg/mL); Sf (12.5 µg/mL); St (6.25 µg/mL)	**131**	[125]
*T. sinensis* (A.Juss.) M. Roem.	Roots	**213**, **13**, **232**, **237**, **254**, **131**, **132**, **260**, **261**, **294**, **305**, **153**, **386**, **388**, **159**	Cytotoxic effect against 786-O and Caki-1 cell lines for 786-O (%viability cell 41.86 at 250 ppm) and Caki-1 (%viability cell 44.73% at 250 ppm)	**153**	[138]
*T. sinensis* (A.Juss.) M. Roem.	Leaves	**220**, **131**, **118**, **199**, **370**, **155**, **385**, **390**, **391**	Not reported	**131**	[142]
*T. sinensis* (A.Juss.) M. Roem.	Leaves	**219**, **220**, **221**, **222**, **236**, **4**, **253**, **131**, **132**, **147**, **282**, **104**, **283**, **307**, **310**, **315**, **316**, **193**, **199**, **361**, **372**, **379**, **392**, **395**, **410**	Antibacterial activity against MSSA (MIC 0.125 mg/mL) and MRSA (MIC 1 mg/mL) Cytotoxic activity against SGC7902 (IC_50_ 70.38 μg/mL); HepG2 (IC_50_ 82.2 μg/mL); HT29 (IC_50_ 99.94 μg/mL)	**131**	[127]
*T. sinensis* (A.Juss.) M. Roem.	Leaves	**215**, **220**, **221**, **227**, **228**, **25**, **229**, **131**, **305**, **307**, **332**, **153**, **160**	Antidiabetic properties effect on medium glucose consumption in the 3T3-L1 cells. The essential oil could increase the use of medium glucose to approximately 50% at 50 µg/mL.	**227**	[129]
*T. ciliata* M. Roem.	Leaves	**220**, **13**, **227**, **25**, **4**, **255**, **131**, **259**, **261**, **265**, **266**, **268**, **119**, **118**, **104**, **114**, **317**, **330**, **361**, **362**, **370**, **155**, **153**, **156**, **160**, **154**, **383**, **385**, **395**, **159**, **401**	Not reported	**314**, **131**, **25**	[**136**]
*T. ciliata* M. Roem.	Leaves	**219**, **220**, **221**, **245**, **253**, **263**, **280**, **281**, **331**, **199**, **361**, **380**, **379**, **385**,	Antidepressant activity using FST and TST immobility as a result of FST (decrease immobility duration > 50 s); TST (decrease immobility duration > 50 s)	**220**, **361**, **222**	[143]
*A. odorata* Lour.	Stems	**13**, **25**, **243**, **131**, **257**	Antimicrobial activity against Bo, Po, and Rs. Bo (MIC values 0.0625–0.5 mg/mL), Po (MIC values 0.0625–0.5 mg/mL), Rs (MIC values 0.0625–0.5 mg/mL); Bo (MFC values 0.25–1 mg/mL), Po (MFC values 0.25–1 mg/mL), Rs (MFC values 0.25–1 mg/mL).	**25**	[144]
*A. indica* (Hook.fil) Harms	Flowers	**221**, **13**, **147**, **148**, **119**, **118**, **104**, **283**, **110**, **317**, **126**, **321**, **193**, **199**, **155**, **380**, **174**, **156**, **160**, **154**, **176**, **159**	Antimicrobial activity against Bs, Ca, and Mg with inhibition zone Bs (10.5 ± 0.5 mm); Ca (14 ± 0.5 mm); Mg (11.0 ± 1.0 mm)	**104**, **193**	[145]
*A. indica* (Hook.fil) Harms	Flowers	**228**, **245**, **257**, **396**	Not reported	**241**	[146]
*C. fissilis* Vell.	Leaves	**220**, **13**, **227**, **25**, **4**, **131**, **132**, **147**, **148**, **104**, **114**, **110**, **296**, **38**, **34**, **317**, **319**, **193**, **199**, **155**, **153**, **156**, **160**, **159**	Antibacterial activity against Sa, Ec, Pa with inhibition zone Sa (9.3 ± 0.6 mm); Ec (6.7 ± 0.6 mm); Pa (inactive)	**314**, **131**	[137]
*C. mexicana* M.Roem	Leaves and Stembarks	**221**, **13**, **227**, **228**, **25**, **230**, **231**, **243**, **245**, **90**, **131**, **132**, **282**, **104**, **283**, **291**, **305**, **321**, **324**, **99**, **155**, **380**, **153**, **160**, **397**, **398**	Not reported	**131**	[141]
*C. odorata* L.	Leaves	**220**, **13**, **223**, **225**, **4**, **253**, **131**, **132**, **271**, **104**, **289**, **290**, **304**, **318**, **325**, **326**, **193**, **199**, **399**, **400**, **405**	Not reported	**405**	[135]
*C. odorata* L.	Essential oil	**221**, **13**, **228**, **4**, **238**, **131**, **147**, **267**, **104**, **283**, **305**, **34**, **317**, **319**, **128**, **321**, **193**, **199**, **376**, **377**, **380**	Not reported	-	[128]
*C. tabularis* A.Juss	Leaves	**218**, **220**, **13**, **245**, **248**, **247**, **253**, **131**, **132**, **261**, **267**, **118**, **104**, **3**, **296**, **38**, **34**, **128**, **327**, **193**, **199**, **338**, **380**, **156**, **154**, **383**, **395**, **159**	Not reported	**132**	[134]
*C. baccifera* (Roth) Miq	Leaves	**220**, **13**, **230**, **231**, **132**, **258**, **261**, **104**, **303**, **311**, **312**, **313**, **314**, **320**, **360**, **367**, **375**, **155**, **380**, **153**, **382**, **397**	Not reported	**131**	[131]
*T. connaroides* (Wight and Arn.) Bentv.	Roots	**131**, **279**, **104**, **114**, **38**, **301**, **302**, **128**, **328**, **193**, **386**	Not reported	**328**	[132]
*T. connaroides* (Wight and Arn.) Bentv.	Barks, Leaves, Roots	**219**, **220**, **25**, **4**, **131**, **147**, **267**, **282**, **104**, **283**, **288**, **305**, **308**, **126**, **328**, **193**, **199**, **361**, **365**, **155**, **154**, **383**, **385**, **387**, **392**, **394**, **397**, **405**, **411**	Antiplasmodial activity as a result of weak antiplasmodial with IC_50_ range 2–22 µg/mL.	**193**, **131**, **328**	[130]
*K. grandifolia* C.DC.	Flowers	**220**, **13**, **224**, **228**, **253**, **132**, **256**, **272**, **273**, **274**, **119**, **285**, **294**, **128**, **328**, **193**, **361**, **365**, **371**, **160**, **154**, **393**, **176**, **159**, **411**	Cytotoxic activity against HepG-2 (IC_50_ 21.6 μg/mL); MCF-7 (IC_50_ 26.1 μg/mL); HCT-116 (37.6 μg/mL)	**132**, **250**, **13**	[133]
*K. senegalensis* (Desv.) A.Juss.	Flowers	**221**, **13**, **224**, **131**, **132**, **261**, **119**, **114**, **296**, **335**, **361**, **371**, **153**, **160**, **387**, **394**, **159**, **403**	Cytotoxic activity against HepG-2 (IC_50_ 61.1 μg/mL); MCF-7 (IC_50_ 79.7 μg/mL); HCT-116 (61.0 μg/mL)	**132**, **131**	
*M. azedarach* L.	Leaves	**253**, **131**, **132**, **317**	Not reported	131	[123]
*M. azedarach* L.	Flowers	**213**, **253**, **131**, **291**, **317**	Antimicrobial activity against Sa, Pv, Pa, Ec, Se, and Kp with MIC values Sa (150 µL/mL); Pv (150 µL/mL); Pa (150 µL/mL); Ec (175 µL/mL); Se (175 µL/mL); Kp (175 µL/mL).	**210**, **209**	[126]
*S. macrophylla* G.King	Flowers	**25**, **132**, **148**, **104**, **126**, **333**, **334**, **193**, **199**, **361**, **155**, **153**, **395**	Not reported	**131**	[122]
*S. macrophylla* G.King	Leaves	**227**, **25**, **238**, **4**, **13**, **259**, **283**, **308**, **317**, **193**, **361**, **380**, **383**, **395**	Not reported	**25**	[140]
*S. macrophylla* G.King	Leaves	**220**, **13**, **224**, **226**, **227**, **25**, **131**, **265**, **147**, **272**, **273**, **119**, **280**, **281**, **104**, **283**, **285**, **294**, **317**, **319**, **128**, **193**, **199**, **361**, **380**, **153**, **154**, **383**, **385**, **395**	Not reported	**25**	[147]
*G. convergen* T.D.Penn.	Branches	**356**, **357**, **358**, **409**	Not reported	**405**, **193**	[148]
*G. kunthiana* A.Juss	Leaves	**153**, **182**, **220**, **193**, **230**, **231**, **254**	Antimicrobial activity against Ec, Pa, Se, Pm, Kp, Sa, Ef, Se, Bs, and Ca with MIC values Ec (Not active); Pa (7000 mg/mL); Se (7000 mg/mL); Pm (7000 mg/mL); Kp (inactive); Sa (13.6 mg/mL); Ef (437.5 mg/mL); Se (3500 mg/mL); Bs (875 mg/mL); Ca (1750 mg/mL) Antioxidant activity using DPPH scavenging capacity with IC_50_ 17.54 ± 0.18 µg/mL	**231**, **256**	[149]
*G. macrophylla* G.King	Leaves	**156**, **131**, **148**, **119**, **110**	Not reported	**89**	[32]
*G. macrophylla* G.King	Leaves	**347**, **348**, **349**, **350**, **351**, **352**, **353**, **354**, **355**	Anti-inflammatory activity using macrophages BALB/c mice with CC 17.7 > 100 µg/mL.	**264**	[150]
*G. macrophylla* G.King	Fruits	**193**, **361**, **153**, **160**, **176**	Not reported	**193**, **176**, **283**	[151]
*G. cedreta* (A.Chev.) Pellegr.	Barks	**193**, **370**, **380**, **160**, **385**, **176**, **131**, **147**, **267**, **118**, **104**, **114**, **296**	Not reported	**131**	[152]
*G. macrophylla* G.King	Leaves	**359**, **388**, **13**, **227**, **147**, **267**, **118**, **281**, **104**, **297**, **346**, **385**	Not reported	**297**, **385**, **104**, **267**, **131**	[153]

Sa (*S. aureus*); Bs (*B. subtilis*); Pv (*P. vulgaris*); Ec (*E. coli*); Kp (*K. pneumoniae*); Sp (*S. pneumoniae*); Pa (*P. aeruginosa*); Sf (*S. flexneri*); St (*S. typhi*); MSSA (*methicillin-sensitive S. aureus*); MRSA (*methicillin-resistant S. aureus*); Bo (*B. oryzae*); Po (*P. oryzae*); Rs (*R. solani*); Ca (*C. albicans*); Mg (*M. gypseum*); Se (*S. enterica*); Pm (*P. mirabilis*); Ef (*E. faecalis*).

The oils from the leaves and stems of *Toona ciliata* contained 36 and 31 components, of which 96% and 92% were identified, respectively. The major compounds in both samples were *β*-caryophyllene (**131**), germacrene-D (**25**), and bicyclogermacrene (**317**). Moreobver, the minor compounds were identified as seychellene (**401**), *β*-bourbonene (**395**), *β*-gurjunene (**383**), *α*-gurjunene (**154**), longifolene (**370**), *β*-cubebene (**361**), cubebol (**362**), *β*-acorenol (**330**), and *Z*-caryophyllene (**255**) along with a minor known compound [136]. From the essential oil of *Cedrela odorata* L., a huge diversity of hydrocarbon sesquiterpenoid including *α*-curcumene (**239**), *β*-caryophyllene (**131**), *γ*-muurolene (**267**), *δ*-cadinene (**104**), *β*-selinene (**34**), *cis*-calmenene (**128**), *trans*-calamenene (**319**), calarene (**376**), ledene (**377**), and alloaromadendrane (**380**) have been reported [128]. The volatile constituent of leaves *Ekebergia capensis* Sparrm. comprises a huge number of sesquiterpenoids, namely 10,10-dimethyl-2,6-dimethylenebicyclo [7.2.0]undecan-5*β*-ol (**336**), 4-isopropyl-6-methyl-1-methylene-1,2,3,4-tetrahydronaphthalene (**337**), 7-acetyl-2-hydroxy-2-methyl-5-isopropylbicyclo [4.3.0]-nonane (**338**), caryophylla-4(12),8(13)-dien-5*α*-ol (**336**), naphthalene-1,6-dimethyl-4-(1-methylethyl) (**340**), and tetracyclo [6.3.2.0(2,5).0(1,8)]tridecan-9-ol, 4,4-dimethyl (**412**) [139]. In addition, from the leaves of *Trichilia degreana* Sond. are 1-naphthalenemethanol, 1,4,4*α*,5,6,7,8,8*α*-octahydro-2,5,5,8a-tetramethyl (**341**), and humulane-1,6-dien-3-ol (**249**); volatile sesquiterpenoids from the leaves of *Turraea floribunda* include (4*S*,8*S*,8*R*)-8-isopropyl-5-methyl-3,4,4*α*,7,8,8*α*-hexahydronaphthalen-2)-methanol (**342**), octahydro-1,4,9,9-tetramethyl (**404**), and azulene, 1,2,3,5,6,7,8,8*α*-octahydro-1,4-dimethyl-7-(1-methylethenyl) (**343**); and from the leaves of *Turraea obtusifolia*, (1*R*,2*R*,4*S*,6*S*,7*S*,8*S*)-8-isopropyl-1-methyl-3-methylene-tricyclo [4.4.0.02,7]decan-4-ol (**413**), 3,5,11-eudesmatriene (**299**), and eudesma-2,4,11-triene (**300**) were identified [139].

Furthermore, from the leaves of *Chukrasia tabularis*, two monocyclic sesquiterpenoids, shyobunone (**246**) and preisocalamendiol (**247**), were produced [134]. Ar-turmerone (**243**), the aromatic sesquiterpenoid, was gained from the stem of *Aglaia odorata* and *Cedrela mexicana* [108,141]. Caryophyllene oxide (**132**), along with another minor compounds of Leaves *Toona sinensis*, include *γ*-cadinene (**282**), *δ*-cadinene (**104**), cadina-1,4-diene (**283**), *γ*-selinene (**307**), bicyclo [4.4.0]dec-1-ene, 2-isopropyl-5-methyl-9-methylene (**310**), 1,2,4*α*,5,6,8*α*-hexahydro-4,7-dimethyl-1-(1-methylethyl)-naphthalene (**315**), *β*-vatirenene (**316**), *α*-copaene (**193**), *β*-cubebene (**361**), 9,10-dehydro-isolongifolene (**372**), *β*-patchoulene (**392**), and 8,9-dehydro-cycloisolongifolene (**410**), as well as the major compounds *β*-caryophyllene (**131**) [127]. The elemol (**255**) was found in abundance as a constituent of four species, such as *Cedrela Mexicana*, *Cedrela odorata*, *Chukrasia tabularis*, and *Toona ciliate* M. Roem. [134,136,141,143]. In addition, 1,1,4,8-tetramethyl-*cis*-4,7,10-cycloundecatriene (**244**) from the flowers of the neem tree has been investigated [146]. The caryophyllene derivative iso-caryophyllene (**253**), *E*-caryophyllene (**254**), and caryophyllenyl alcohol (**256**) were found to be minor compounds from *Cedrela odorata*, *Toona sinensis*, and *Kaya grandifoliola*, respectively [133,135,138]. Himachalane-type sesquiterpenoids *α*-himachalane (**257**) were produced by three species, including *Aglaia odorata*, *Azadirachta indica* A. Juss, and *Cedrela odorata*, while *β*-himachalene (**258**) was produced by the leaves of *Cipadessa baccifera* [131,136,144,146].

In addition, *α*-guaiene (**262**) was investigated from leaves of *Toona sinensis* and *Cedrela odorata* [125,136], and *β*-guaiene (**263**) was determined from *Toona sinensis* and *Toona ciliata* [125,143]. *Cis*-4(14),5-Muuroladiene (**273**), a muurolane-hydrocarbon type sesquiterpenoid, was recognized in several species, including *Cedrela odorata*, *Cedrela fissilis*, *Swietenia macrophylla*, and *Kaya grandifoliola* [133,136,154]. Moreover, *cis*-muurol-5-en-4-*β*-ol (**274**) was also found in the flower of *Kaya grandifoliola* [133]. The *α*-cubenol (**279**) was investigated as a minor component of roots of *Trichilia connaroides* [132]. From the leaves of *Toona sinensis*, two hydrocarbon-cadinane types, *α*-cadinene (**280**) and *β*-cadinene (**281**), were investigated [125]. Moreover, *α*-*cis*-bergamotene (**289**) and *β*-*trans*-bergamotene (**3**) were investigated from leaves of *Cedrela odorata* L. [135]. *Γ*-eudesmol (**297**) is also found in stem barks of *Trichilia monadelpha* [132]. The selinene-type 7-epi-*α*-selinene (**308**) and selin-11-en-4*α*-ol (**309**) come from the leaves of *Cedrela odorata* [136]. From the roots of *Trichilia connaroides*, two-sesquiterpenoids, hydrocarbon khusinol (**301**), occidentalol (**302**), and *β*-chamigrene (**328**), were also reported [132].

From the leaves of *Cedrela odorata* amorpha-4,11-diene (**304**), isobicyclogermacrene (**318**), *β*-acoradiene (**325**), *β*-alaskene (**326**), *β*-funebrene (**399**), mintsulphide (**400**), *β*-santalene (**322**), and *α*-santalene (**405**) were investigated [135]. The amorphane-type sesquiterpenoid *α*-amorphene (**303**), along with bicyclo [3.1.1]hept-2-ene, 2,6-dimethyl-6-(4-Methyl-3-pentenyl) (**311**), bicyclo [6.3.0]undec-1(8)-en-3-on,2,2,5,5-tetramethyl (**312**), bicyclo [5.2.0] nonane, 2-methylene-4,8,8-trimethyl-4-vinyl (**313**), 6*β*-bicyclo [4.3.0]nonane, 5*β*-iodomethyl-1 *β*-isopropenyl-4 *α*,5 *α*dimethyl (**314**), 1S,cis-calamenene (**320**), *β*-copaen-4*α*-ol (357), 2,2,7,7-tetramethyl-tricyclo [6.2.1.0 1,6]undec-4-en-3-one (**375**), isoledene (**397**), *β*-copaene (**359**), and isospathulenol (**382**), was also found in leaves of *Cipadessa baccifera* [131]. From the leaves and pericarps of *Trichilia connaroides*, *δ*-selinene (**306**) was also found as the minor product [130]. A known compound calamenene (**126**) was investigated in three species, including *Azadirachta indica*, *Trichilia connaroides*, and *Swietenia macrophylla* [122,130,145]. Santalol derivative *α*-santalol (**406**) and *β*-santalol (**323**) were produced by *Trichilia dregeana* and *Turraea floribunda* [139]. The volatile constituent of flowers *Swietenia macrophylla* cadala-1(10),3,8-triene (**333**) and 7-isopropenyl-1,4-dimethyl-1,2,3,3α,4,5,6,7-octahydroazulene (**334**) were investigated [122]. The torreyol (**329**) was found in the leaves of *Toona sinensis* [125]. From the flower of the genus *Khaya*, selin-11-en-4*α*-ol (**309**) and cadalene (**335**) have been produced [133]. The sesquiterpenoids of the essential oil of *Toona ciliata α*-cyperone (**331**) have been investigated [143].

From the leaves of *Chukrasia tabularis*, the minor compound eremophyllene (**327**) was also found [134]. *α*-longipinene (**365**) and *β*-longipinene (**366**) were found in the stem bark of *Trichilia monadelpha*, while both of them were also found in several species, including *Trichilia connaroides*, *Khaya grandifoliola*, and *Cedrela fissilis* [130,133,136]. The other longipinene derivative, longicamphenylone (**371**), was investigated from *Kaya grandifoliola* and *Khaya senegalensis* as the minor compound [133]. Shilaluke et al. found ledene oxide-(II) (**378**) from *Turraea obtusifolia* [139]. The *α*-aromadendrene (**379**) was also found in the essential oil of *Toona ciliata* [143]. Moreover, the roots of *Toona sinensis* produced *α*-cedrene (**386**) and ylangene (**385**) [138,142]. From the leaves of *Swietenia macrophylla*, as well as from the roots of *Trichilia connaroides*, the minor compound *β*-cedrene (**388**) was investigated[130]. Minor volatile sesquiterpenoids palustrol (**174**) and viridiflorene (**176**) were found in flowers of *Azadirachta indica* [145]. Souda et al. reported two tricyclic sesquiterpenoids *γ*-patchoulene (**391**), *α*-bourbonene (**394**) and cedr-8-(15)-en-9-alpha-ol (**403**), and one tetracyclic sesquiterpenoid, cyclosativene (**411**), from the flowers of *Khaya senegalensis* (Desv.) A. Juss [133]. Moreover, aristolene (**390**) and patchoulene (**391**) were investigated from *Toona sinensis* [142]. Cedrol (**388**) and *α*-cedrol (**389**) were gained from the roots and leaves *Toona sinensis* [125,138]. From the leaves and pericarps of *Trichilia connaroides, α*-bourbonene (**394**) was identified [130]. The Tricyclic sesquiterpenoids cedrane diol (**401**) was isolated from the leaves of *Cedrela odorata* [136]. Furthermore, from the roots of *Cedrela mexicana*, ylanga-2,4(15)-diene (**398**) was also reported [141].

The essential oil of the leaves of *Guarea macrophylla* produced a number of bicyclic sesquiterpenoids, including 6,9-guaiadiene (**347**), *trans*-muurola-4(14),5-diene (**348**), *δ*-amorphene (**349**), *β*-calacorene (**350**), 1,10-di-epi-cubenol (**351**), *α*-acorenol (**352**), *cis*-cadin-4-en-7-ol (**353**), hinesol (**354**), isolongifolan-7-*α*-ol (**408**), and valerianol (**355**) [150]. Moreover, the bicyclic sesquiterpenoids *γ*-amorphene (**346**) was investigated from leaves of *Guarea macrophylla* [153]. Furthermore, a minor compound, cadina-1(6),4-diene (**345**), was only reported as a volatile compound from the fruits of *Guarea macrophylla* [155]. Magalhães et al. isolated bicyclic sesquiterpenoids, including *cis*-caryophyllene (**356**), from the leaves of *Guarea scabra* A.Juss; drima-7,9(11)-diene (**357**) from branches of *Guarea convergens* T.D.Penn; and caryophyllene epoxide (**358**) from branches of *Guarea humatensis* [148]. Furthermore, (*E*)-iso-*γ*-bisabolene (**250**), as well as tricyclic sesquiterpenoid mustakone (**409**), was found in the branches of *Guarea sylvatica* C.DC. [148]. In addition, *epi*-globulol (**407**) was identified from two species, *Guarea macrophylla* and *Guarea cedrata* (A.Chev.) Pellegr. [32,152].

## 4. Ethnobotany and Medicinal Uses

The Meliaceae family is widely distributed mainly in Indo-Malesia, Southeast Asia, Northern Europe, Africa-America, and Australia and comprises approximately 58 genera and 740 species that are mostly used to treat various diseases traditionally. Moreover, *Aglaia* genus is used extensively in the form of decoction and powders in traditional health settings. These include the healing of wounds, fevers, influenza, cough, and other skin disease [21,44,71]. A previous study also discovered that several *Amoora* species had been used as folk medicines in Southeast Asia for the treatment of many diseases such as diarrhea, inflammation, spleen and liver, and cardiac diseases [63,83]. In certain Chinese regions, some species from the *Aphanamixis* genus have been used as a primitive medicine for colds, rheumatoid arthritis, and numbness from cold temperatures [156]. Furthermore, plants from the *Chisocheton* genus are used traditionally for the treatment of several ailments, including stomach and kidney complaints, backache, fever, rheumatism, and malaria [72]. The genus *Cipadessa*, which includes nine species, is known to be the folk medicine to treat dysentery, malaria, pruritus, rheum, rheumatism, and burns and scalds by Dai, a Chinese ethnic minority [157]. Several species in the *Cedrela* genus have widespread cultural uses for diabetes, digestive system disorder, parasitic worms, liver diseases, and hypertension [31]. Moreover, the genus *Dysoxylum* is also known as traditional medicine used to treat diarrhea, leprosy, aches, pain, and lung hemorrhages [158]. The bark of *Entandrophragma cylindricum* species is also reported to be commonly used to treat bronchitis, lung complaints, colds, and edema and is also used as an anodyne [94]. The wood bark of the *Guarea* species is employed in folk medicine as an abortive and febrifugal agent, and the leaves and fruits are reported to be quite toxic to cattle [48,159]. Moreover, in the Philippines, the dried fruit peel was burned and used as a mosquito repellent, and the fruit skin was also used as an arrow poison [52]. *Toona* species are mainly commonly used for the treatment of ulcers and asthma [160]. The known Brazilian genus *Trichilia* was a huge potential for the production of new drugs and herbal medicines. Several species of *Trichilia* have been used in folk medicine in the treatment of diseases such as liver disorders, purgative, antiepileptic, antipyretic, antimalarial, physical and mental tonic, and aphrodisiac and sexual stimulants [161,162]. The *Walsura* genus is commonly used as folk medicine in Thailand with potent antioxidant activity [163].

## 5. Biological Activity

As previously shown, a total of 211 isolated and 202 volatile sesquiterpenoids were determined by complete spectra data as well as by MS database from the Meliaceae family. At present, several bioactivity studies have been reported to evaluate cytotoxic activity, antimicrobial activity, and antioxidant activity; antidiabetic, antiplasmodial, and antiviral activity of isolated compounds (Table 2); and major volatile sesquiterpenoids constituents on essential oil (Table 1).

### 5.1. Cytotoxic Activity

Meliaceae sesquiterpenoids were investigated for their biological activity in cytotoxic analyses, which were carried out on over 61 isolated compounds from 20 species. A further investigation against the human myeloid leukemia HL-60, hepatocellular carcinoma SMMC-7721, human lung cancer A-549, human breast cancer MCF-7, and SW480 cells lines through MTT method on acyclic sesquiterpenoid (**2**) was inactive against the five cell lines with IC_50_ values at >50 µM [38]. The bisabolene-type compound (**7**) was evaluated for cytotoxic activities against the K562 human chronic myelogenous leukemia cell line through the MTT method. The result showed that no significant cytotoxicity was observed with IC_50_ values at >50 µM, in contrast to quercetin (IC_50_ values at 2.5 ± 0.5 µM) [164]. In other investigations, the cytotoxic effects of three humulene-type sesquiterpenoids (**17**), (**19**–**20**) were tested against three human cancer cell lines in vitro. The result showed that only (**17**) exhibited a slow proliferating cytotoxic effect against MCF-7 (IC_50_ values at 78 ± 15 µM), respectively, compared to the IC_50_ values of thapsigargin IC_50_ 2.9 nM, but showed a significant effect against S180 murine sarcoma cell lines (IC_50_ values at 7 ± 3 µM) using thapsigargin as a positive control for comparison [5]. Moreover, two other compounds were inactive with IC_50_ values > 50 µM against L5178Y mouse lymphoma cells using the MTT method [50]. Ionone derivative (**26**) showed inactive activity with IC_50_ values > 50 µM towards HL-60 cell lines with camptothecin as a positive control (IC_50_ values 0.01 ± 0.0001 µM) [53]. Moreover, the cytotoxic investigation of sesquiterpene aldehyde (**31**) was conducted only by Fadhilah et al. against three cell lines, T-47D, WiDR, and Hep-G2, through the MTT method with modification. The result showed that compound (**31**) exhibits the strongest cytotoxic activity against T-47D cell lines with IC_50_ values 39.1 ± 1.5 µg/mL compared to doxorubicin (IC_50_ values 0.21 ± 0.02 µg/mL), while inactive through others cell lines (IC_50_ values > 50 µg/mL) [56]. Furthermore, the aldehyde substituted is the potential to increase the cytotoxic activity of monocyclic sesquiterpenoids groups.

The cytotoxic effect of eudesmane-type (**58**) was evaluated in vitro against the MCF-7 cell line through the MTT method using cisplatin as the positive control with IC_50_ values > 50 µM. The result showed that the compound exhibited higher cytotoxic activity than cisplatin, with IC_50_ values of 27.3 µM [77]. Sinaga et al. also reported two eudesmane-type compounds (**36**) and (**44**) cytotoxic activity towards the MCF-7 cell line [64]. The result showed that compound (**44**) exhibited moderate cytotoxicity against MCF-7 cells with IC_50_ values of 17.9 µg/mL, while the others showed weak cytotoxicity with IC_50_ values of 121.65 µg/mL. The presence of hydroxyl and olefinic groups possibly played some important structural features for cytotoxic activity against MCF-7 cells in eudesmane-type sesquiterpenoids [64]. Furthermore, two compounds, (**46**–**47**), showed no activity (IC_50_ values > 50 µM) towards human gastric carcinoma SGC-7901, K562, and human hepatocellular carcinoma BEL-7402 cells with paclitaxel as a positive control (IC_50_ values 1.9; 7.4; 2.6 µM), respectively, while compound (**53**) was inactive against HL-60 [42,71]. Similarly, the anti-tumor activities of eudesmane-types (**45**) and (**48**) were evaluated against HeLa cervical cancer cells and DU145 prostate cancer cells using PrestoBlue^®^ reagent assay. The result showed that all compounds did not exhibit in vitro cytotoxicity against the cell lines with IC_50_ values > 50 µg/mL [70]. Moreover, the cytotoxic activity of compound (**51**) was evaluated through the HT-29 human colon cancer cell line by the sulforhodamine B (SRB) protein staining method, with paclitaxel as the positive control. The result showed that compound (**51**) was inactive with ED_50_ > 10 µM). However, paclitaxel showed strong inhibition with ED_50_ 0.0006 µM [73].

Moreover, the cytotoxic investigation of hydroxylated guaiane-types (**80**–**85**) and (**87**) were conducted only by Liu et al. against SGC-7901, K-562, and BEL-7042 cell lines through MTT assay [71]. The result showed that compounds (**80**–**81**) and (**87**) exhibit modest cytotoxic activity against the SGC-7901 cell line with IC_50_ values 38.8, 40.0, and 38.0 µM, respectively, while others were larger than 50 µM and paclitaxel at range 1.9–7.4 µM for all cell lines tested. The cytotoxic activity of four guaiane-type (**83**), (**87**), (**91**), and (**92**) were also reported against MCF-7 cell lines. The result showed that (**83**), (**87**), and (**91**) were inactive with IC_50_ larger than 100 µM compared to cisplatin with an IC_50_ value of 53 µM. Moreover, compound (**92**) showed potential activity against MCF-7 as well as HeLa cell lines with IC_50_ values ranging from 29.6 to 39.6 µM, respectively [3,77]. Compound (**88**) merely showed inactive against HeLa, Vero kidney epithelial cell, and U937 human myeloid leukemia cell lines, with IC_50_ values more than 100 µM compared to actinomycin D with IC_50_ ranging from 1.9 to 8.8 µM, respectively [165].

Compound (**94**) showed no activity against HT-29 cell lines with ED_50_ values more than 50 μM through the sulforhodamine B (SRB) protein staining method, with paclitaxel as the positive control with ED_50_ values of 0.001 μM [89]. A total of three isoleucines, (**97**–**98**) and (**101),** were tested the cytotoxic activity against HL-60 and A549 cell lines through MTT assay, and the result showed that all compounds had no significant cytotoxicity lower than 50 µM [42].

The cytotoxic activity of the cadinane-type (**115**–**116**) and calamenene-type (**124**–**125**) against MCF-7 and HeLa cell lines was also reported using the Resazurin (PrestoBlue) method. Compound (**115**–**116**) exhibited moderate cytotoxic activities against MCF-7 with an IC_50_ value of 38.79 ± 0.22 µM, as well as against HeLa with an IC_50_ value of 39.31 ± 0.14 for compound (**115**) and significant cytotoxic activities against MCF-7 with IC_50_ value 45.14 ± 0.12 µM and against HeLa with IC_50_ value 41.82 ± 0.38 µM for compound (**116**), respectively. The aldehyde group attached to C4 on cadinane derivative possibly remained increasing cytotoxic activity for cadinane type [3]. Two calamenene-type compounds (**124**–**125**) showed lower activity with IC_50_ values ranging from 80.6 up to 100 µM; moreover, all compounds’ IC_50_ values were compared to cisplatin with IC_50_ values of 53.0 and 16.0, respectively [3]. Another potential cytotoxic compound (**114**) was tested against the MCF-7 cell line with inhibition activity values of 33.46 µM using cisplatin as a positive control with an IC_50_ value of 53.0 µM [77]. Two calamenene-type (**121**–**122**) were evaluated in vitro cytotoxic activity against the HL-60 cell line through MTT assay using camptothecin with IC_50_ value 0.01 ± 0.0001 µM. The result showed that compound (**121**) exhibited medium cytotoxicity with an IC_50_ value of 18.25 ± 1.52 µM, while another compound showed lower activity with values up to 100 µM [53,103]. In a cytotoxicity test using Resazurin (PrestoBlue) cell viability assay, compound (**117**) had an IC_50_ of 3375.6–6086.3 µM against HeLa and B16-F10 cell lines. The IC_50_ value of cisplatin was 19.0–43.0 µM [6]. Furthermore, compound (**103**) was evaluated for cytotoxic activity against two cell lines, such as HL-60 and A549 cell lines, through MTT assay. The result showed no activity with IC_50_ value > 100 µM [42].

The cytotoxic activities of caryophyllene-type (**129**) and (**133**) were evaluated in vitro against MCF-7 and B16-F10 using the MTT viability assay. The result showed that compound (**129**) with the revised method showed potential activity against MCF-7. Moreover, compound (**133**) demonstrated no significant cytotoxicity up to 100 µM against the B16-F10 cell line [95,99]. These results indicated that the cytotoxic activity of caryophyllene-type sesquiterpenoid is affected by the presence of double bonds, epoxide, and configuration of methyl groups. A minor bicyclic sesquiterpenoid murolene-type (**148**) showed no activity against thirty-seven human tumor cell lines, including 1218L, T24, 498NL, SF268, HCT116, HT29, 251L, 536L, 1121L, 289L, 526L, 529L, 629L, H460, 401NL, MCF7, DA231, 276L, 394NL, 462NL, 514L, 520L, 1619L, 899L, OVCAR3, 1657L, PANC1, 22RV1, DU145, LNCAP, PC3M, 1752L, 1781L, 393NL, 486L, 944L, and 1138L. The compound was tested using the revised MTT method, with IC_50_ values > 10 μg/mL [168]. Moreover, the cytotoxic investigation of tetralone-type (**149**) was conducted by Sofian et al. [103]. The result demonstrated lower cytotoxicity with IC_50_ value > 50 µM. Camptothecin, which was used as the positive control, gave the cytotoxic against HL-60 at the IC_50_ value 0.01 ± 0.0001 µM. Two oppositane-type (**141**) and (**144**) were reported cytotoxic activity against three cell lines, including HL-60, A549, and MCF-7, through MTT assay. The result showed that compound (**144**) was not active against HL-60 and A549 through MTT cell viability assay [42]. Moreover, compound (**141**) possessed lower cytotoxic activity against MCF-7 with an IC_50_ value of 201.57 μg/mL compared to doxorubicin with an IC_50_ value of 0.17 μg/mL [64].

A further investigation against the HL-60, MCF-7, and A549 cell lines through the MTT method on three aromadendrane-type with a hydroxylated substituent in the C10 (**157**–**158**), and (**160**) showed that (**157**) was the most potent significant cytotoxic compound against three cell lines at IC_50_ value 3.1, 32.5, and 30.4 µM, respectively. Compound (**158**) also demonstrated selective cytotoxic IC_50_ values against all cell lines with IC_50_ values at 14.3, 39.7, and 31.3 µM, respectively. Furthermore, compound (**160**) showed selective potential cytotoxic activity against the A549 cell line with an IC_50_ value of 32.5 µM and weak cytotoxic activity up to 50 µM for the other cell lines. The selectivity shown by (**157**) and (**158**), which oxidized at C12, was absent in (**160**). Therefore, the moieties of 3-isopropylpentanoic in aromadendrane are essential for the HL-60, MCF-7, and A549 selectivity [109]. A previous study also evaluated (**159**) an aromadendrane-type against medulloblastoma cell line Daoy, MCF-7, and A549 by an MTT assay, where the compound exhibited promising cytotoxicity against all cell lines with IC_50_ value 0.1, 10, and 30 µM. In this study, the mechanism of compound (**159**) action was also evaluated on the cell apoptosis by Annexin V-488 staining assay. The result showed that (**159**) induced apoptotic, upon exposure to concentrations ranging from 30 mM to 300 mM, and early and late apoptotic cell death was induced in a concentration-dependent manner in Daoy (55.8–72.1%), MCF-7 (36.2–72.7%), and A459 (35–98.9%) cell lines, respectively [169]. Moreover, compound (**172**) was tested against HL-60 cancer cells by MTT assay. Based on the results, compound (**172**) showed no significant cytotoxicity with an IC_50_ value of up to 20 µM compared to cisplatin with an IC_50_ value of 1.14 µM [112]. Another cytotoxic assay based on the MTT method is compound (**153**) against MCF-7 cell line with cisplatin as a positive control (IC_50_ 53.0 µM). The result showed that compound (**153**) has a promising cytotoxic compound with an IC_50_ value of 12.17 µM [77]. A total of two compounds were tested against several cancer cell lines using Resazurine (PrestoBlue) cytotoxicity assay. Based on the result, compound (**166**–**167**) showed no significant activity against the MCF-7 cell line using cisplatin as a positive control with IC_50_ value 53 µM. Even though compound (**166**) exhibited lower cytotoxic than (**167**), it indicated that *β* alcohol and *α* hydroxymethyl orientation on C10 were responsible for the effect of activity compared to the opposite [72]. Moreover, the cytotoxic activity of compound (**165**) was evaluated against B16-F10 compared to cisplatin with IC_50_ value 12.9 µM [99]. The result showed that compound (**165**) possessed significant cytotoxic with a lower IC_50_ value of 44.8 µg/mL, while Naini et al. also reported cytotoxic activity of the same compound against MCF-7 and HeLa cell lines with IC_50_ range value of 10.37–10.83 µM, respectively [3]. Furthermore, the cytotoxic effect of compounds (**164**) and (**168**) were investigated by the sulforhodamine B (SRB) protein staining method, with ellipticine as the positive control was 0.35 µg/mL against three cell lines including epidermoid carcinoma KB, small-cell lung cancer NCI-H187 and BC. The results showed that compounds (**164**) and (**168**) were considered inactive because they possess an IC_50_ up to 50 µM [110]. Additionally, the cytotoxic activity of compound µ was evaluated against HeLa, liver cell cancer SK-Hep1, and B16 using an alamar blue assay. The result showed compound (**175**) did not show cytotoxic activity against those cell lines [172]. The results of a further investigation of compound (**174**) through the tryphan blue staining assay showed that no cytotoxic acitvity was observed against the lymphoma cell line with LD_50_ ≥ 3.60 mM compared to bleomycin as positive control (LD_50_ 0.02 mM) [171].

The minor tricyclic sesquiterpenoid (**194**) also demonstrated selective cytotoxicity against HeLa using Resazurin assay with cisplatin as a positive control (IC_50_ value 2.18 µM), while the derivate compound (**195**) cytotoxicity was also evaluated against human liver cancer HepG using SRB assay and the result was inactive compared to camptothecin as the positive control [118,173].

The cytotoxic investigation of dimeric sesquiterpenoids (**203**–**205**) was conducted only by Sofian et al. against HL-60 through MTT assay. The result showed that the compound (**203**) had the most potent cytotoxic activity with an IC_50_ value of 39.04 µM, while two others (**204**–**205**) were inactive (IC_50_ values > 50 µM) compared with camptothecin as a positive control [53]. The IC_50_ value of compound (**203**) was lower than its monomeric compound (**121**); this implied that the activity of sesquiterpenoids phenol derivatives decreased with increasing molecular weight. Thus, compound (**121**) promised an important role in exhibiting the HL-60 cancer cell line. Moreover, another three dimeric sesquiterpenoids cytotoxicity, compounds (**201**–**202**) and (**206**), were evaluated against MCF-7 and HeLa cell lines. The result showed that compound (**206**) was the most selective cytotoxic against MCF-7 with an IC_50_ value of 40.56 µM, followed by compound (**201**) with an IC_50_ value of 41.54 µM, and compound (**202**) showed no significant activity compared to cisplatin as a positive control. In contrast, compound (**202**) showed more potent inhibition of HeLa proliferation with an IC_50_ value of 13.00 µM, followed by compound (**201**) with an IC_50_ value of 22.15 µM when compound (**206**) showed lower activity (IC_50_ value of 39.32 µM) [3]. In addition, two trimeric sesquiterpenoid (**207**–**208**) cytotoxicity were evaluated against HL-60 cell lines using MTT assay. The result showed both of them considered inactive compared to camptothecin with an IC_50_ value of 0.01 µM [103]. Moreover, the cytotoxic effect of compound (**209**) showed the most potent cytotoxic activity against MCF-7 and HeLa cell lines with IC_50_ values 12.07 ± 0.17 µM and 9.29 ± 0.33 µM, while compound (**211**) showed moderate activity with IC_50_ values of 31.59 ± 0.34 µM and 27.93 ± 0.25 µM. Moreover, the cytotoxic activity of (**210**) is a selective inhibitor against the HeLa cell growth with an IC_50_ value of 39.72 ± 0.18 µM. All compounds are compared to cisplatin with IC_50_ values 53.00 ± 0.02 and 16.00 ± 0.01 µM [120].

Several essential oils possessed cytotoxic activities, including essential oil from *Toona sinensis* roots with major constituent (**153**) against 786-O ccRCC cell lines and Caki-1 metastatic cell lines via MTT assay. The result showed that at 250 ppm, 786-O cells were retracted after 24 h of treatment, with % viability cells of 41.86% compared to ethanol as vehicle control. Moreover, at 250 ppm, Caki-1 cells were retracted after 48 h of treatment, with % viability cells of 44.73% compared to ethanol as vehicle control [138]. The GC-MS analysis showed that leaves of *Toona sinensis* contain a high amount of (**131**) and showed potential cytotoxic activities against SGC7901, HepG2, and HT29 with IC_50_ values of 70.38, 82.2, and 99.94 µg/mL, respectively [127]. Furthermore, the cytotoxic investigation of essential oil of flowers *Khaya grandifolia* and *Khaya senegalensis* was tested against HepG2, MCF-7, and HCT-116 using MTT assay with doxorubicin as a positive control. The result showed that the essential oil of *Khaya grandifolia* flowers is more potent than *Khaya Senegalensis*, with IC_50_ values of 37.2, 21.8, and 52.8 µg/mL, respectively [133]. The sesquiterpenoid content of *Khaya grandifolia*, namely (**132**), may contribute to their cytotoxic activity. In addition, the leaves oil *Guarea macrophylla* showed toxicity against peritoneal macrophages of BALB/c mice with 50% cytotoxic concentration (CC_50_) ranging from 17.7 to 100 µg/mL. The major component of leaves oil was (**261**) with 18% of the major compound [150].

### 5.2. Anti-Inflammatory

A total of 15 sesquiterpenoid compounds possessed anti-inflammatory activity, produced from eight species in the Meliaceae family. Aphanamoxene D (**1**) is a nor-sesquiterpenoid of the monocyclic group with relatively significant anti-inflammation activity. Compound (**1**) was determined for the inhibitory effect on NO production induced by LPS in a macrophage cell line RAW264.7, and cell viability was tested by the MTT method [37]. Furthermore, the bisabolane-type compounds (**11**–**12**) and isodaucane-type (**95**) showed significantly inhibited LPS-induced inflammation in BV-2 microglial cells using Greiss assay at 5 and 10 µM (*p* < 0.01). At 20 µM, all compounds could reduce the level of NO up to 53.75%, 22.58%, and 10.58%, respectively, compared to resveratrol as a positive control with a reduced value of 55.61% [44]. Moreover, compound (**13**) has an important inhibitory effect in vivo technique for anti-inflammatory activities using rats in the carrageenan-induced paw edema method. As a result, compound (**13**) reduced the production of prostaglandin E_2_ (PGE_2_), as well as inducible nitric oxide synthase (iNOS) and cyclooxygenase (COX-2) expression induced by the intraplantar injection of carrageenan in rats. All the anti-inflammatory assays were compared to dexamethasone-treated animals as a positive control [174]. An investigation of compound (**51**) was evaluated for anti-inflammatory activity using an enzyme-based ELISA NF-*k*B assay through p65 (RelA) inhibitory activity. The result showed that compound (**51**) exhibited IC_50_ values of >20 µM and was considered inactive [73]. Three years later, Pan et al. evaluated compound (**94**) with the same method. As a result, compound (**94**) was extremely active (ED_50_ 0.005), where the value was 10 times more potent than rocaglamide as a positive control (ED_50_ 0.08 µM) [89]. Moreover, the anti-inflammatory effect of compound (**175**) was evaluated by Western blot analysis against the accumulation of pro-inflammatory iNOS and COX-2 proteins in RAW264.7 macrophage cells. The result showed that the compound (**175**) could not reduce the accumulation of iNOS protein induced by LPS in comparison with control cells stimulated by LPS only [172]. Furthermore, compound (**90**) inhibited the production of proinflammatory cytokines, as well as the expression of iNOS and COX-2 in microglia using Western blot analysis [166]. In addition, compound (**172**) was inactive for the evaluation of the inhibitory effect on NO production induced by LPS in a macrophage cell line RAW264.7 with IC_50_ value > 20 µM [112]. Moreover, compound (**27**) determined the immunosuppressant effect on *Jurkat* cells in vitro. The result showed that compound (**27**) significantly inhibited calcineurin (CN) at an inhibition rate of 58.06% [175]. In addition, Hua et al. reported the effects of eight compounds’, (**68**–**75**) and (**152),** anti-inflammatory activities through in vitro analysis on NO production induced by LPS in macrophage cell line RAW264.7 and on the release of IL-1*β* using interleukin-1*β* Assay kit. As a result, all compounds exhibited no cytotoxicity, NO inhibition, and IL-1*β* inhibitory activity [82].

### 5.3. Antioxidant

There are three species in the Meliaceae family that produce eight antioxidant compounds. Moreover, the bisabolane-type (**7**) was evaluated for inhibition of free radicals by using CLPAA and cellular antioxidant activity (CAA) assay. The result showed that compound (**7**) had a significantly weaker inhibitory activity with IC_50_ > 100 µM for both assays [164]. Moreover, the antioxidant activity of three furanoeremophilane-type sesquiterpenoids (**186**–**188**) was investigated with NADH-dependent mitochondrial and NADPH-dependent microsomal lipid peroxidation. The result showed that compound (**186**) had a more potent antioxidant activity with IC_50_ values of 16.4 and 41.6 µM, followed by compound (**187**) with IC_50_ values of 59.7 and 54.3 µM, and then compound (**188**) with IC_50_ values of 71.7 and 74.4, respectively [116]. Furthermore, the antioxidant activity of two compounds, (**179**) and (**183**), were evaluated against mitochondrial lipid peroxidation induced by Fe (III)-ADP/NADH. The result showed that compound (**179**) was merely active with IC_50_ values of 76.8 and may be useful as a lead compound in the field of medicinal chemistry, while compound (**183**) showed no activity [115]. In addition, the leaves oil of *Guarea kunthiana* showed moderate antioxidant activity using DPPH (2,2-diphenyl-1-picrylhydrazyl) free radical assay with IC_50_ value 17.54 ± 0.18 µg/mL compared to BHT as control with IC_50_ value 9.27 ± 0.08 µg/mL [149].

### 5.4. Antidiabetic

Eleven sesquiterpenoids (**11**–**12**), (**28**), (**48**), (**50**), (**87**), (**94**–**95**), (**150**), and (**135**) from only one species of *Aglaia lawii* had evaluated for antidiabetic assay with PTP1B inhibitory activity bioassay using oleanolic acid as the positive control. The result showed that compound (**12**) exhibited inhibitory activity against PTP1B protein with an IC_50_ value of 16.05 ± 1.09 µM, while the other compounds were inactive with IC_50_ values ≥ 50 µM [44]. In addition, the antidiabetic potential of leaves *Toona sinensis* was shown to prevent the progression of diabetes and hepatosteatosis, the rise of triglycerol levels, and the decrease in adiponectin in type 2 diabetic mice. The major constituent (**224**) sesquiterpenoids may be effective in preventing type 2 diabetes [129].

### 5.5. Antimicrobial

Antimicrobial activities, including antibacterial or antifungal activities, play a pivotal role in controlling emerging diseases [176]. Compound (**2**) was evaluated for antimicrobial activity against four microorganisms, including *Staphylococcus aureus*, *Pseudomonas aeruginosa*, MRSA92, and MRSA98. The minimum inhibitory concentrations (MICs) of the compound were determined by the two-fold dilution method. The result showed that compound (**2**) was inactive against all the tested strains with MIC values > 50 µg/mL [38]. Furthermore, a germacrane-type sesquiterpenoid compound (**25**) was tested against five microbial, including *Pseudomonas aeruginosa* UPCC 1244, *Bacillus subtilis* UPCC 1149, *Escherichia coli* UPCC 1195, *Staphylococcus aureus* UPCC 1143, *Candida albicans* UPCC 2168, *Trichophyton mentagrophytes* UPCC 4193, and *Aspergillus niger* UPCC 3701 using agar well method. The result showed that compound (**25**) was moderately active against the fungi *C. albicans* and *A. niger*, as well as lowly active against the fungus *T. mentagrophytes*. Moreover, compound (**25**) was shown to be inactive against bacteria *S. aureus*, *E. coli*, and *B. subtilis*, while moderately active against *P. aeruginosa* [52]. Two isodaucane-type sesquiterpenoids compounds (**94**) and (**102**) were evaluated for antimicrobial activities, including *S. aureus*, MRSA 92^#^ (MRSA, methicillin-resistant *S. aureus*), MRSA 98^#^, and MRSA 111^#^ using the agar plate punch assay. As a result, compounds (**94**) and (**102**) showed no activities [90]. Two calamenene-type compounds, (**121**) and (**123**), showed significant antibacterial activity against *Bacillus subtilis* with a MIC value of 28 µM, which compared to amoxicillin as a positive control with a MIC value of 34 µM. Moreover, both compounds showed weak antibacterial activity with a MIC value range of 57–114 µM against *Bacillus subtilis*, *Escherichia coli*, *Pseudomonas aeruginosa*, *Salmonella typhi*, *Shigella dysenteriae*, *Staphylococcus aureus*, and *Vibrio cholerae*. In addition, both compounds were also tested for their antifungal properties against two wood-rotting fungi (brown rot, *F. palustris*, and white rot, *T. versicolor*) using a zone inhibition assay at two concentration (0.46 and 4.58 mM) and with periodical observation up to 14 days. Compound (**121**) showed growth inhibition at day 5 for both fungi, and compound (**123**) showed growth inhibition at day 5 only for white-rot fungi but not for brown-rot fungi [93]. An investigation of antimicrobial activity was showed by compound (**160**) against two bacteria, including *B. subtilis* and *X. vesicatoria*, using calorimetric assay by chromogenic reagent MTT. The result showed that compound (**160**) possessed inhibitory activities at IC_50_ range values of 158.0–737.2 µg/mL, while compound (**160**) showed an obvious dose-dependent inhibition of mycelial growth for which IC_50_ range values of 21–50 µg/mL against four tested fungi, including *Alternaria solani, Fusarium graminearum, Rhizoctonia solani*, and *V. pirina* [170]. Three aromadendrane-type compounds, (**164**) and (**167**–**168**), were evaluated for antimicrobial activities against *Mycobacterium tuberculosis* H_37_Ra using the microplate alamar blue method. As a result, compounds (**168**), (**167**), and (**164**) showed antimicrobial activities with MIC values of 50, 100, and 50 µg/mL, respectively [110].

The essential oil of *Naregamia alata* roots showed potential antibacterial against Gram-positive bacteria *Staphylococcus aureus* MTCC No. 740 and *Bacillus subtilis* MTCC No. 441, and Gram-negative bacteria *Proteus vulgaris* MTCC No. 426, *Escherichia coli* MTCC No. 443, and *Klebsiella pneumoniae* MTCC No. 109. Moreover, the major sesquiterpenoid composition was reported, namely (**256**), (**131**), and (**227**). The result showed that *Naregamia alata* roots exhibited diameter of zone inhibition in 11, 12, 10, 13, and 11 mm, respectively [121]. The essential oil of *Toona sinensis* leaves, also known as Chinese *Toona*, were investigated for their antimicrobial activity against *Staphylococcus aureus* ATCC 25923, *Streptococcus pneumoniae* ATCC 46919, *Escherichia coli* ATCC 25922, *Pseudomonas aeruginosa* ATCC 27853, *Shigella flexneri* ATCC 1202, and *Salmonella typhi* ATCC 50013. The result showed that the essential oil of *Toona sinensis* leaves exhibited active antibacterial activities with MIC values of 1.57, 1.57, 3.13, 3.13, 12.50, and 6.25 µg/mL, respectively [125]. Moreover, from the leaves of *Toona sinensis*, antibacterial activity against two strains of *Staphylococcus aureus* ATCC 25923 and ATCC 43300, methicillin-resistant *Staphylococcus aureus* (MRSA), and methicillin-sensitive *Staphylococcus aureus* (MSSA), were also reported, with MIC values of 0.5, 4, 1, and 0.125 µg/mL, respectively. The major compound of *Toona sinensis* leaves essential oil was investigated as (**99**) [127]. The stem of *Aglaia odorata* produced volatile major sesquiterpenoids (**25**), which represent 20.3% of the oil component. Antimicrobial activities of the stem oil were investigated against *Bacillus cereus* ATCC 11778, *Staphylococcus aureus* ATCC 25923, *Acinetobacter baumannii* ATCC 19606, and *Escherichia coli* ATCC 25922, as well as three rice fungal pathogens, *Bipolaris oryzae*, *Pyricularia oryzae*, and *Rhizoctonia solani*, using broth microdilution method. The result showed that *Toona sinensis* stem oil exhibited significant antifungal activity against three rice pathogens with MIC and MFC values ranging from 0.0625 to 0.5 and 0.25 to 1 mg/mL, respectively. Moreover, the oil showed an inactive result towards the bacteria. Furthermore, the antimicrobial activity of the oil from the *Aglaia odorata* stem can be attributed to the presence of (**25**), (**13**), (**254**), and (**131**), which is constituted a high amount in this oil [144]. Furthermore, the *Azadirachta indica* flowers oil showed moderated antibacterial activities against *Bacillus subtilis* (ATCC 6633), *Candida albicans* (ATCC 10231), and *Microsporum gypseum* (clinically isolated) with the diameter clear zones of 10.5, 14, and 11.0 mm, respectively. The major constituent of this oil was found to be (**104**) and (**193**), which represent 9.43 and 7.03% of the oil component [145]. The leaves oil of *Cedrela fissilis* with (**314**) as a major constituent also showed antibacterial activities against *Staphylococcus aureus* (ATCC25923), *Escherichia coli* (ATCC-25922), and *Pseudomonas aeruginosa* (ATCC9027) compared to cloramphenicol as a positive control. The result showed that leaves oil of *Cedrela fissilis* inhibited *Staphylococcus aureus* and *Escherichia coli* with inhibition zone 9.3 and 6.7 mm, respectively. Moreover, the leaves oil and stembarks oil showed no activity toward *Pseudomonas aeruginosa*. In the same analysis, the stembarks of *Cedrela fissilis* also reported no activities against *Staphylococcus aureus* and *Escherichia coli* [137]. The contrasting antibacterial result from the two samples may contribute to the different chemical constituents, while the stembarks of *Cedrela fissilis* contain (**4**) as a major component [137]. In vitro, antimicrobial activities were conducted against microbial strains, including *Staphylococcus aureus*, *Proteus vulgaris*, *Pseudomonas aeruginosa*, *Escherichia coli*, *Salmonella enterica*, *Klebsiella pneumoniae*, *Pichia guilliermondii*, and *Candida albicans* from the flower oil of *Melia azedarach* using agar well diffusion method. The result showed moderate activity with a MIC value range of 150–200 µL/mL compared to gentamicin as a positive control with a MIC value of 50 µL/mL with (**210**) as a major compound [126]. In addition, antimicrobial activities of leaves oil *Guarea kunthiana* using the broth microdilution method was reported against *Escherichia coli* ATCC 25922, *Salmonella enterica* subsp., *Enterica* ATCC 14028, *Pseudomonas aeruginosa* ATCC 27853, *Proteus mirabilis* ATCC 25933, *Klebsiella pneumoniae* ATCC 13883, *Staphylococcus aureus* ATCC 25923, *Enterococcus faecalis* ATCC 19433, *Staphylococcus epidermidis* ATCC 12228, *Bacillus subtilis* CCD-04, and *Candida albicans* ATCC 10231. The result showed that the leaf oil was effective against all the microorganisms tested with a MIC value range of 13.6–7000 mg/mL, except for the bacteria *Escherichia coli* and *Klebsiella pneumoniae* [149].

### 5.6. Antiviral

It is necessary to develop new antiviral compounds to treat viruses that have no antiviral therapy [177]. Substantially, only four compounds, (**76**–**77**), (**113**), and (**134**), were evaluated for antiviral activities. In addition, compounds (**76**–**77**) were investigated for their HIV-inhibitory in vitro by XTT-based assay, while none of them showed any activities at a concentration of 50 µg/mL [83]. The antiviral activities of compounds (**113**) and (**134**) were investigated against the HIV-1_IIIB_ virus using the green fluorescent protein (GFP)-based HOG.R5 reporter cell line. The result showed inhibition activities of both compounds [167].

### 5.7. Antiplasmodial

Promising antiplasmodial compounds from natural products have been developed [178]. In general, a humulene-type compound (**18**) was evaluated for antiplasmodial activity against *Plasmodium falciparum* 3D7 strain merozoites using a chloroquine-sensitive assay, which compared to chloroquine as a positive control (IC_50_ 3 nm). As a result, compound (**18**) showed selective activity as antiplasmodial with an IC_50_ value of 76 ± 10 µM [5]. In vitro antiplasmodial of the pericarps extract of *Trichilia conaroides* was tested against the K1 strain of *Plasmodium falciparum* compared to chloroquine as a positive control. The result showed that dichloromethane pericarps extract was effective antiplasmodial with IC_50_ values 6.92 µg/mL with (**131**) as a major compound [130].

### 5.8. Antidepressant-Like Activity

The discovery of antidepressant-like activity is necessary with fewer side and better efficacy [143]. The antidepressant activity was evaluated from the leaves oil *Toona ciliate* Roem. var yunnanensis with (**217**), (**358**), and (**219**) as major compounds using forced swimming test FST and tail suspending test TST method. The result from FST and TST demonstrated that the immobility time could be significantly reduced by leaves oil with a concentration range of 10–80 mg/kg without accompanying changes in ambulation when assessed in the open field test OFT. In addition, the contents of dopamine, 5-hydroxytryptamine, and brain-derived neurotrophic factor in the hippocampus of chronic mild stress rats could be increased by treatment leaves oil at doses of 20–80 mg/kg. The result showed that the leaf oil of *Toona ciliate* could be considered a new candidate for curing depressive disorder [143].

## 6. Conclusions

The sesquiterpenoids constituent and biological activities of the Meliaceae family were investigated for up to 55 years. Presently, approximately 413 of the compounds have been obtained from hydrodistillation and isolation processes, including 211 isolated and 202 volatile compounds. The sesquiterpenoids were isolated from 24 genera, consisting of major type sesquiterpenoids including eudesmane, aromadendrane, cadinane, guaiane, bisabolane, furanoeremophilane, humulene, caryophyllene, germacrane, and oppositane. It also consists of minor type sesquiterpenoids, such as acyclic-skeleton, ionone, megastigmane, seco-guaiane, isodaucane, calamenene, murolene, muurolol, tetralone, cadalene, hydro-azulene, sabinene, copaene, clovane, dimeric, and trimeric. Eudesmane-type was identified as the most compound (27%), and it is also used as a chemical marker. The literature review also reported fascinating sesquiterpenoids frameworks from three genera, including *Aglaia*, *Dysoxylum*, and *Trichilia*. Meliaceae family plants exhibit interesting biological activities, including cytotoxic, anti-plasmodial, antimicrobial, antidiabetic, anti-viral, and anti-inflammatory effects. However, cytotoxic against various human cancer cells was recognized as the most common activity, resulting from the presence of virindiflorol an aromadendrane-type sesquiterpenoid. In addition, furanoeremophilane derivatives have been synthesized biosynthetically. According to our study, there is a need to investigate sesquiterpenoid compounds in higher plants in greater depth, particularly in the Meliaceae family, since they have a unique structure and diverse biological activities, which is important for identifying compounds that may be used in drug development.

## Figures and Tables

**Table 2:** Isolated sesquiterpenoids from Meliaceae family and the bioactivities.

Acyclic Sesquiterpenoids				
Compounds	Species	Parts of Plant	Biological Activity	Ref.
**1**	*A. polystachya* (Wall.) R.Parker	Seeds	Anti-inflammatory activity against RAW 264.7 macrophage IC_50_ 14.2 ± 0.9 μM	[37]
**2**	*A. grandifolia* Blume	Leaves and stems	Cytotoxic activity against HL-60 (IC_50_ > 50 μM), SMMC-7721 (IC_50_ > 50 μM), A-549 (IC_50_ > 50 μM), MCF-7 (IC_50_ > 50 μM), SW480 (IC_50_ > 50 μM) Antibacterial activity against Sa, Pa: inactive	[38]
Monocyclic Sesquiterpenoids				
Compounds	Species	Parts of Plant	Biological Activity	Ref.
**7**	*C. boiviana* Baill.	Stembarks	Cytotoxic activity against K562 (IC_50_ > 50 μM) Antioxidant assay CLPAA (IC_50_ > 100 μM), CAA (IC_50_ > 100 μM)	[41,164]
**11**	*A. lawii* (Wight) C.J.Saldanha	Twigs and leaves	Anti-inflammatory activity BV-2 microglial cell (22.58% at 20 μM) Antidiabetic assay PTP1B (IC_50_ ≥ 50 μM)	[44]
**12**	*A. lawii* (Wight) C.J.Saldanha	Twigs and leaves	Anti-inflammatory activity BV-2 microglial cell (10.58% at 20 μM) Antidiabetic assay PTP1B (IC_50_ 16.05 ± 1.09 μM)	[44]
**13**	*G. guidonia* (L.) Sleumer *G. macrophylla* G.King *T. lepidota* Mart	Stembarks Stembarks Stems	Anti-inflammatory assay reducing the edema formation induced by carrageenan (300 μg/paw), an effect observed at 30, 60, 120, and 240 min	[46] [45] [47]
**17**	*T. emetica* (Forssk.) Vahl	Roots	Cytotoxic activity against S180 (IC_50_ 7 ± 3 mM); MCF-7 (IC_50_ 78 ±15 mM)	[5]
			Antiplasmodial activity against *Plasmodium falciparum* (IC_50_ 76 ± 10 mM)	
**19**	*T. monadelpha* (Thonn.) J. De Wild	Leaves and root barks	Cytotoxic activity against L5178Y (IC_50_ > 50 μM)	[50]
**20**	*T. monadelpha* (Thonn.) J. De Wild	Leaves and root barks	Cytotoxic activity against L5178Y (IC_50_ > 50 μM)	[50]
**25**	*L. domesticum* Correa	Fruit Peel	Antibacterial activity against Sa (inactive); Ec (inactive); Bs (inactive)	[52]
**26**	*D. parasiticumi* (Osbek). Kosterm	Leaves	Cytotoxic activity HL-60 (IC_50_ > 50 μM)	[53]
**27**	*A. grandifolia* Blume	Stems	CN activity with inhibition rate of 58.06%, strong immunosuppressive effect on Con A-induced anti-inflammatory activity murine splenocytes and PMA/IO-induced jurkat cells significantly reduced NFAT1 protein expression and downstream gene. Immunosuppressant activity reduced IL-2 expression in the CN/NFAT signaling pathway	[38,54]
**28**	*A. lawii* (Wight) C.J.Saldanha	Twigs and leaves	Antidiabetic activity PTP1B IC_50_ ≥ 50 μM (inactive)	[44]
**31**	*L. domesticum* Correa	Fruit peel	Cytotoxic activity aginst T-47D (IC_50_ 39.18 ± 1.54 µg/mL), WiDr (IC_50_ > 50 μg/mL), Hep-G2 (IC_50_ > 50 μg/mL)	[56]
Bicyclic Sesquiterpenoids				
Compounds	Species	Part of Plant	Biological Activity	Ref.
**36**	*A. minahassae* Koord.	Stembarks	Cytotoxic activity against MCF-7 (IC_50_ 121.65 µg/mL)	[59]
	*C. cinerascens* (Pellegr.) Hand.-Mazz.	Branches		[60]
	*C. baccifera* (Roth) Miq	Stems		[61]
	*G. guidonia* (L.) Sleumer	Seeds		[62]
	*A. tsangii* (Merr.) X.M.Chen	Twigs and leaves		[63]
	*L. domesticum* Correa	Stembarks		[64]
	*D. densiflorum* (Blume) Miq	Twigs and leaves		[65]
	*D. parasiticum* Correa	Stembarks		[3]
	*T. africanus* (Wele. Ex C.DC.) Pellegr.	Stembarks		[66]
	*A. grandis* (Korth. Ex Miq) Pierre	Stembarks		[6]
**44**	*G. guidonia* (L.) Sleumer	Seeds	Cytotoxic activity against MCF-7 (IC_50_ 17.97 µg/mL)	[62]
	*L. domesticum* Correa	Stembarks		[64]
**45**	*A. elaeagnoidea* (A.Juss.) Benth.	Stembarks	Cytotoxic activity against HeLa (IC_50_ 3544 µg/mL); DU145 (IC_50_ 971.69 µg/mL)	[70]
	*G. guidonia* (L.) Sleumer	Seeds		[62]
**46**	*A. odorata* Lour.	Twigs	Cytotoxic activity against SGC-7901 (IC_50_ > 50 µM); K-562 (IC_50_ > 50 µM); BEL-7402 (IC_50_ > 50 µM)	[71]
**47**	*A. odorata* Lour.	Twigs	Cytotoxic activity against SGC-7901 (IC_50_ > 50 µM); K-562 (IC_50_ > 50 µM); BEL-7402 (IC_50_ > 50 µM)	[71]
**48**	*A. lawii* (Wight) C.J.Saldanha	Twigs and leaves	PTP1B inhibitory (IC_50_ > 50 µM)	[44]
	*A. elaeagnoidea* (A.Juss.) Benth.	Stembarks	Cytotoxic activity against HeLa (IC_50_ 9010.62 µg/mL); DU145 (IC_50_ 16,883.7 µg/mL)	[70]
**50**	*A. lawii* (Wight) C.J.Saldanha	Twigs and leaves	Antidiabetic activity PTP1B inhibitory (IC_50_ > 50 µM)	[44]
**51**	*A. foveolata* Pannel	Stembarks	Cytotoxic activity against HT-29 (ED_50_ >10 µM)	[73]
			Anti-inflammatory activity NF-kB p65 (RelA) IC_50_ > 20 µM	
**52**	*D. excelsum* Blume	Leaves	Cytotoxic activity against KB (IC_50_ 49.4 mg/mL); MCF-7 (IC_50_ 37.8 mg/mL)	[42]
**53**	*D. excelsum* Blume	Leaves	Cytotoxic activity against HL-60 (inactive)	[42]
**58**	*D. parasiticum* (Osbek). Kosterm	Stembarks	Cytotoxic activity against MCF-7 (IC_50_ 27.39 μM)	[77]
**68**	*D. densiflorum* (Blume) Miq	Fruits	Antiinflammatory activity against RAW 264.7 macrophage inactive; IL-1*β* inactive	[82]
**69**	*D. densiflorum* (Blume) Miq	Fruits	Antiinflammatory activity against RAW 264.7 macrophage inactive; IL-1*β* inactive	[82]
**70**	*D. densiflorum* (Blume) Miq	Fruits	Antiinflammatory activity against RAW 264.7 macrophage inactive; IL-1*β* inactive	[82]
**71**	*D. densiflorum* (Blume) Miq	Fruits	Antiinflammatory activity against RAW 264.7 macrophage inactive; IL-1*β* inactive	[82]
**72**	*D. densiflorum* (Blume) Miq	Fruits	Antiinflammatory activity against RAW 264.7 macrophage inactive; IL-1*β* inactive	[82]
**73**	*D. densiflorum* (Blume) Miq	Fruits	Antiinflammatory activity against RAW 264.7 macrophage inactive; IL-1*β* inactive	[82]
**74**	*D. densiflorum* (Blume) Miq	Fruits	Antiinflammatory activity against RAW 264.7 macrophage inactive; IL-1*β* inactive	[82]
**75**	*D. densiflorum* (Blume) Miq	Fruits	Antiinflammatory activity against RAW 264.7 macrophage inactive; IL-1*β* inactive	[82]
**76**	*A. rohituka* (Roxb.) Wight and Arn.	Stembarks	Antiviral activity HIV-inhibitory (>50 mg/mL)	[82]
**77**	*A. rohituka* (Roxb.) Wight and Arn.	Stembarks	Antiviral activity HIV-inhibitory (>50 mg/mL)	[82]
**80**	*A. odorata* Lour.	Twigs	Cytotoxic activity against SGC-7901 (SGC-7901 IC_50_ 38.8 µM); K-562 (IC_50_ > 50 µM); BEL-7402 (IC_50_ > 50 µM)	[71]
**81**	*A. odorata* Lour.	Twigs	Cytotoxic activity against SGC-7901 (SGC-7901 IC_50_ 40.0 µM); K-562 (IC_50_ > 50 µM); BEL-7402 (IC_50_ > 50 µM)	[71]
**82**	*A. odorata* Lour.	Twigs	Cytotoxic activity against SGC-7901 (IC_50_ > 50 µM); K-562 (IC_50_ > 50 µM); BEL-7402 (IC_50_ > 50 µM)	[71]
**83**	*A. odorata* Lour.	Twigs	Cytotoxic activity against SGC-7901 (IC_50_ > 50 µM); K-562 (IC_50_ > 50 µM); BEL-7402 (IC_50_ > 50 µM)	[71]
	*D. parasiticum* (Osbek). Kosterm	Stembarks	Cytotoxic activity against MCF-7 (IC_50_ > 100 ± 0.37 µM); HeLa (IC_50_ >100 ± 0.36 µM)	[3]
**84**	*A. odorata* Lour.	Twigs	Cytotoxic activity against SGC-7901 (IC_50_ > 50 µM); K-562 (IC_50_ > 50 µM); BEL-7402 (IC_50_ > 50 µM)	[71]
**85**	*A. odorata* Lour.	Twigs	Cytotoxic activity against SGC-7901 (IC_50_ > 50 µM); K-562 (IC_50_ > 50 µM); BEL-7402 (IC_50_ > 50 µM)	[71]
**87**	*D. parasiticum* (Osbek). Kosterm	Stembarks	Cytotoxic activity against MCF-7 (IC_50_ 208.06 μM)	[77]
	*A. odorata* Lour.	Twigs	Cytotoxic activity against SGC-7901 (IC_50_ 38.0 µM); K-562 (IC_50_ > 50 µM); BEL-7402 (IC_50_ > 50 µM)	[71]
	*A. lawii* (Wight) C.J.Saldanha	Twigs and leaves	Antidiabetic activity PTP1B (IC_50_ ≥ 50 μM)	[44]
	*D. excelsum* Blume	Leaves		[42]
	*C. baccifera* (Roth) Miq	Stems		[61]
	*C. lasiocarpus* (Miq) Valeton	Stembark		[72]
**88**	*G. kunthiana* A.Juss	Leaves	Cytotoxic activity against HeLa (IC_50_ > 100 μM); Vero (IC_50_ > 100 μM); U937 (IC_50_ > 100 μM)	[85,165]
	*T. sinensis* (A.Juss.) M. Roem.	Pericarps		[68]
	*T. sinensis* (A.Juss.) M. Roem.	Pericarps		[69]
	*C. cumingianus*	Twigs		[76]
	*D. densiflorum* (Blume) Miq	Twigs and Leaves		[65]
**90**	*G. kunthiana* A.Juss	Leaves	Western blot and RT-PCR analyses of anti-inflammatory activity showed that alismol markedly inhibited iNOS and COX-2 expression at both mRNA and protein levels as well as NO and PGE2 production. At 100 μM, alismol almost completely blocked LPS-induced iNOS, COX-2, PGE2, and NO induction.	[85,166]
**91**	*D. parasiticum* (Osbek). Kosterm	Stembarks	Cytotoxic activity against MCF-7 (IC_50_ 183.69 µg/mL)	[77]
**92**	*D. parasiticum* (Osbek). Kosterm	Stembarks	Cytotoxic activity against MCF-7 (IC_50_ 39.61 ± 0.29 µM); HeLa (IC_50_ 26.06 ± 0.21 µM)	[3]
	*G. macrophylla* M. Vahl	Stembarks		[90]
**94**	*A. foveolata* Pannel	Barks	Cytotoxic activity against HT-29 (ED_50_ > 20 μM)	[89]
	*A. perviridis* Hiern	Fruits, leaves, twigs, and roots	Antidiabetic activity PTP1B inhibitory (IC_50_ > 50 µM)	[92]
	*A. lawii* (Wight) C.J.Saldanha	Twigs and leaves	Antibacterial activity against Sa (inactive); MRSA 92 (inactive); MRSA 98 (inactive); MRSA 111 (inactive)	[91]
	*W. robusta* Roxb.	Leaves	Anti-inflammatory activity Nf-Kb (ED_50_ 0.005 μM)	[93]
**95**	*A. lawii* (Wight) C.J.Saldanha	Twigs and leaves	Antidiabetic activity PTP1B inhibitory (IC_50_ > 50 µM) Anti-inflammatory activity BV-2 microglial cell (53.75% at 20 μM)	[44]
**96**	*A. lawii* (Wight) C.J.Saldanha	Twigs and leaves	Antidiabetic activity PTP1B inhibitory (IC_50_ > 50 µM)	[44]
**97**	*A. elaeagnoidea* (A.Juss.) Benth.	Twigs and Leaves	Cytotoxic activity against HL-60 (inactive); A549 (inactive)	[92]
	*D. excelsum* Blume	Leaves		[42]
**98**	*A. elaeagnoidea* (A.Juss.) Benth.	Twigs and Leaves	Cytotoxic activity against HL-60 (inactive); A549 (inactive)	[92]
	*D. excelsum* Blume	Leaves		[42]
**101**	*D. excelsum* Blume	Leaves	Cytotoxic activity against HL-60 (inactive); A549 (inactive)	[42]
**102**	*W. robusta* Roxb.	Leaves	Antibacterial activity Sa (inactive); MRSA 92 (inactive); MRSA 98 (inactive); MRSA 111 (inactive)	[91]
**103**	*D. densiflorum* (Blume) Miq	Twigs and leaves	Cytotoxic activity against HL-60 (inactive); A549 (inactive)	[86]
	*D. excelsum* Blume	Leaves		[42]
**113**	*A. tsangii* (Merr.) X.M.Chen	Twigs and leaves	Antiviral activity anti-HIV (HOG.R5 IC_50_ 10 μg/mL)	[95]
**114**	*D. parasiticum* (Osbek). Kosterm	Stembarks	Cytotoxic activity against MCF-7 (IC_50_ 33.46 μM)	[77]
**115**	*D. parasiticum* (Osbek). Kosterm	Stembarks	Cytotoxic activity against MCF-7 (IC_50_ 38.79 ± 0.22 µM); HeLa (IC_50_ 39.31 ± 0.14 µM)	[3]
**116**	*D. parasiticum* (Osbek). Kosterm	Stembarks	Cytotoxic activity against MCF-7 (IC_50_ 45.14 ± 0.12 µM); HeLa (IC_50_ 41.82 ± 0.38 µM)	[3]
**117**	*A. grandis* (Korth. Ex Miq) Pierre	Stembarks	Cytotoxic activity against HeLa (IC_50_ 6086.3 µM); B16-F10 (IC_50_ 3375.63 µM)	[6]
**121**	*D. parasiticum* (Osbek). Kosterm	Leaves	Cytotoxic activity against HL-60 (IC_50_ 18.25 ± 1.52 µM)	[53]
	*D. densiflorum*	Seeds	Antimicrobial activity against Bs (MIC 28 µM); Sa (MIC 57 µM); Ec (MIC 57 µM); Pa (MIC 114 µM); St (MIC 114 µM); Sd (MIC 114 µM); Vc (MIC 57 µM)	[94]
**122**	*D. parasiticum* (Osbek). Kosterm	Leaves	Cytotoxic activity against HL-60 (IC_50_ 119.85 ± 10.03 µM)	[103]
**123**	*D. densiflorum* (Blume) Miq	Seeds	Antimicrobial activity against Bs (MIC 28 µM); Sa (MIC 57 µM); Ec (MIC 57 µM); Pa (MIC 114 µM); St (MIC 114 µM); Sd (MIC 114 µM); Vc (MIC 57 µM)	[94]
**124**	*D. parasiticum* (Osbek). Kosterm	Stembarks	Cytotoxic activity against MCF-7 (IC_50_ 80.66 ± 0.23 µM); HeLa (IC_50_ 82.18 ± 0.35 µM)	[3]
**125**	*D. parasiticum* (Osbek). Kosterm	Stembarks	Cytotoxic activity against MCF-7 (IC_50_ > 100 ± 0.33 µM); HeLa (IC_50_ > 100 ± 0.44 µM)	[3]
	*D. shciffneri* F. Muell.	Wood		[8]
**129**	*A. harmsiana* Perkins	Stembarks	Cytotoxic activity against MCF-7 (IC_50_ 0.62 μM)	[95]
	*A. leucophylla* King	Stembarks		[100]
	*G. macrophyla* M. Vahl	Stembarks		[90]
	*M. pinata* Harms	Whole plant		[101]
**133**	*A. simplicifolia* (Bedd.) Harms	Stembarks	Cytotoxic activity against B16-F10 (IC_50_ 483.2 μg/mL)	[99]
**134**	*A. grandifolia* Blume	Fruit peels	Anti-HIV (HOG.R5 IC_50_ 10 μg/mL)	[100,167]
	*C. baccifera* (Roth) Miq	Stems		[61]
	*D. excelsum*	Leaves		[42]
**135**	*A. lawii* (Wight) C.J.Saldanha	Twigs and leaves	Antidiabetic activity PTP1B inhibitory (IC_50_ >50 µM)	[44]
	*A. grandifolia* Blume	Fruit peel		[104]
	*D. densiflorum* (Blume) Miq	Twigs and leaves		[88]
	*D. excelsum* Blume	Leaves		[42]
	*T. africanus* (Wele. Ex C.DC.) Pellegr.	Stembarks		[66]
**141**	*L. domesticum* Correa	Stembarks	Cytotoxic activity against MCF-7 (IC_50_ 201.57 µg/mL)	[64]
**144**	*D. excelsum* Blume	Leaves	Cytotoxic activity against HL-60 (inactive); A549 (inactive)	[42]
**148**	*C. odorata* L.	Stems	Cytotoxic activiy against 1218L, T24, 498NL, SF268, HCT116, HT29, 251L, 536L, 1121L, 289L, 526L, 529L, 629L, H460, 401NL, MCF7, DA231, 276L, 394NL, 462NL, 514L, 520L, 1619L, 899L, OVCAR3, 1657L, PANC1, 22RV1, DU145, LNCAP, PC3M, 1752L, 1781L, 393NL, 486L, 944L, and 1138L; Not active (IC_50_ > 10 μg/mL)	[92,168]
	*C. toona R*oxb	Timber		[31]
**149**	*D. parasiticum* (Osbek). Kosterm	Leaves	Cytotoxic activity against HL-60 (IC_50_ >150 µM)	[103]
	*C. baccifera* (Roth) Miq	Stembarks		[41]
**150**	*A. lawii* (Wight) C.J.Saldanha	Twigs and leaves	Antidiabetic activity PTP1B inhibitory (IC_50_ >50 µM)	[44]
**152**	*D. densiflorum* (Blume) Miq	Fruits	Antiinflammation activity RAW 264.7 macrophage inactive; IL-1*β* inactive	[82]
Tricyclic Sesquiterpenoids				
Compounds	Species	Part of Plant	Biological Activity	Ref.
**153**	*D. parasiticum* (Osbek). Kosterm	Stembarks	Cytotoxic activity against MCF-7 (IC_50_ 12.17 μM)	[77]
	*A. minahassae* Koord.	Stembarks		[59]
	*A. foveolata* Pannel	Barks		[88]
	*A. forbesii* King	Seeds		[105]
	*A. forbesii* King	Leaves		[108]
	*G. macrophylla* M. Vahl	Wood		[87]
	*G. macrophylla* M. Vahl	Leaves		[45]
	*S. koetjape* Merr.	Stems		[98]
	*T. hirta* L.	Fruits		[107]
	*T. lepidota* Mart	Stems		[47]
**157**	*D. densiflorum* (Blume) Miq	Barks	Cytotoxic activity against HL-60 (IC_50_ 3.1 µM); MCF-7 (IC_50_ 32.5 µM); A549 (IC_50_ 30.4 µM)	[109]
**158**	*D. densiflorum* (Blume) Miq	Barks	Cytotoxic activity against HL-60 (IC_50_ 14.3 µM); MCF-7 (IC_50_ 39.7 µM); A549 (IC_50_ 31.3 µM)	[109]
**159**	*A. silvestris* (M. Roem.) Merr.	Leaves	Cytotoxic activity against Daoy (IC_50_ 0.1 µM); MCF-7 (IC_50_ 10 µM); A549 (IC_50_ 30 µM	[102,169]
	*D. densiflorum* (Blume) Miq	Barks	Cytotoxic activity against Daoy (55.8–72.1%), MCF-7 (36.2–72.7%), and A459 (35–98.9%)	[109,169]
**160**	*D. densiflorum* (Blume) Miq	Barks	Cytotoxic activity against HL-60 (IC_50_ >50 µM); MCF-7 (IC_50_ >50 µM); A549 (IC_50_ 32,5 µM)	[109]
			Antimicrobial activity against As (IC_50_ 47.1 µg/mL); Fo (IC_50_ 114.3 µg/mL); Fg (IC_50_ 53.4 µg/mL); Rs (IC_50_ 56.9 µg/mL); Vp (IC_50_ 21.8 µg/mL); Xv (IC_50_ 158.0 µg/mL); Bs (IC_50_ 737.2 µg/mL)	[109,170]
**164**	*C. penduliflorus* Planch.	Wood and leaves	Antibacterial activity against *Mycobacterium tuberculosis* H37Ra (MIC 50 µg/mL)	[110]
	*D. densiflorum* (Blume) Miq	Twigs and leaves	Cytotoxic activity against NCI-H187 (IC_50_ > 50 µM); BC (IC_50_ > 50 µM); KB (IC_50_ > 50 µM)	[88]
**165**	*A. simplicifolia* (Bedd.) Harms	Stembarks	Cytotoxic activity against B16-F10 (IC_50_ 44.8 µg/mL)	[99]
	*A. grandis* (Korth. Ex Miq) Pierre	Leaves	Cytotoxic activity against HeLa (IC_50_ 10.83 ± 0.17 µM); MCF-7 (IC_50_ 10.37 ± 0.11 µM)	[115]
	*D. parasiticum* (Osbek). Kosterm	Stembarks		[3]
	*A. grandis* (Korth. Ex Miq) Pierre	Stembarks		[6]
**166**	*A. lawii* (Wight) C.J.Saldanha	Twigs and leaves	Cytotoxic activity against MCF-7 (IC_50_ 231.99 ± 5.40 µM)	[44]
	*C. penduliflorus* Planch.	Wood and leaves	PTP1B (IC_50_ ≥ 50 μM)	[111]
	*C. lasiocarpus* (Miq) Valeton	Stembark		[72]
**167**	*C. lasiocarpus* (Miq) Valeton	Stembark	Cytotoxic activity against MCF-7 (IC_50_ 258.70 ± 2.49 µM)	[72]
	*C. penduliflorus* Planch.	Wood and leaves	Antibacterial activity against *Mycobacterium tuberculosis* H37Ra (MIC 50 µg/mL)	[110]
**168**	*C. penduliflorus* Planch.	Wood and leaves	Mycobacterium tuberculosis H37Ra (MIC 50 µg/mL)	[110]
			Cytotoxic activity against NCI-H187 (IC_50_ > 50 µM); BC (IC_50_ > 50 µM); KB (IC_50_ > 50 µM)	
**172**	*T. pubescens* Hell.	Twigs	Cytotoxic activity against HL-60 (IC_50_ > 20 µM)	[112]
			Antiinflammatory activity inhibiton of NO production in RAW 264.7 (IC_50_ > 20 µM)	
**174**	*G. macrophylla* M. Vahl	Leaves	Cytotoxic activity Lymphoma (LD_50_ ≥ 3.60 mM)	[45,171]
			Toxicity against *Artemia Salina* (LD_50_ ≥ 3.60 mM)	
**175**	*T. maynasiana* C.DC.	Leaves	Cytotoxic activity against HeLa (inactive); SK-Hep1 (inactive); B-16 (inactive)	[55,172]
			Did not reduce the accumulation of iNOS protein induced by LPS in concentrations 1, 10, 100 µM	
**179**	*T. cuneata* Radlk.	Stembarks	Antioxidant activity Fe(III)–ADP/NADH (IC_50_ 76.8 mM)	[116]
**183**	*T. cuneata* Radlk.	Stembarks	Antioxidant activity Fe(III)–ADP/NADH (inactive)	[116]
**186**	*T. cuneata* Radlk.	Stembarks	NADH-dependent mitochondrial (IC_50_ of 16.4 µM); NADPH-dependent microsomal lipid peroxidations (IC_50_ of 41.6 µM)	[117]
**187**	*T. cuneata* Radlk.	Stembarks	NADH-dependent mitochondrial (IC_50_ of 59.7 µM); NADPH-dependent microsomal lipid peroxidations (IC_50_ of 54.3 µM)	[117]
**188**	*T. cuneata* Radlk.	Stembarks	NADH-dependent mitochondrial (IC_50_ of 71.7 µM); NADPH-dependent microsomal lipid peroxidations (IC_50_ of 74.7 µM)	[117]
**194**	*A. simplicifolia* (Bedd.) Harms	Stembarks	Cytotoxic activity against HeLa (IC_50_ 2.18 µM)	[118]
	*A. harmsiana* Perkins	Stembarks		[99]
**195**	*A. elaeagnoidea* (A.Juss.) Benth.	Bark	Cytotoxic activity against HepG2 (IC_50_ > 30 µM)	[118]
Others sesquiterpenoids				
Compounds	Species	Part of Plant	Biological Activity	Ref.
**201**	*D. parasiticum* (Osbek). Kosterm	Stembarks	Cytotoxic activity against MCF-7 (IC_50_ 41.54 ± 0.34 µM); HeLa (IC_50_ 22.15 ± 0.22 µM)	[3]
**202**	*D. parasiticum* (Osbek). Kosterm	Stembarks	Cytotoxic activity against MCF-7 (IC_50_ > 100 ± 0.27 µM); HeLa (IC_50_ 13.00 ± 0.13 µM)	[3]
**203**	*D. parasiticum* (Osbek). Kosterm	Leaves	Cytotoxic activity against HL-60 (IC_50_ 39.04 ± 3.12 µM)	[53]
**204**	*D. parasiticum* (Osbek). Kosterm	Leaves	Cytotoxic activity against HL-60 (IC_50_ >50 µM)	[53]
**205**	*D. parasiticum* (Osbek). Kosterm	Leaves	Cytotoxic activity against HL-60 (IC_50_ >50 µM)	[53]
**206**	*D. parasiticum* (Osbek). Kosterm	Stembarks	Cytotoxic activity against MCF-7 (IC_50_ 40.56 ± 0.24 µM); HeLa (IC_50_ 39.32 ± 0.25 µM)	[3]
**207**	*D. parasiticum* (Osbek). Kosterm	Leaves	Cytotoxic activity against HL-60 (IC_50_ >150 µM)	[103]
**208**	*D. parasiticum* (Osbek). Kosterm	Leaves	Cytotoxic activity against HL-60 (IC_50_ >150 µM)	[103]
**209**	*D. parasiticum*(Osbek). Kosterm	Stembarks	Cytotoxic activity against MCF-7 (IC_50_ 12.07 ± 0.17 µM); HeLa (IC_50_ 9.29 ± 0.33 µM)	[120]
**210**	*D. parasiticum* (Osbek). Kosterm	Stembarks	Cytotoxic activity against MCF-7 (IC_50_ >100 ± 0.27 µM); HeLa (IC_50_ 39.72 ± 0.18 µM)	[120]
**211**	*D. parasiticum* (Osbek). Kosterm	Stembarks	Cytotoxic activity against MCF-7 (IC_50_ 31.59 ± 0.34 µM); HeLa (IC_50_ 27.93 ± 0.25 µM)	[120]

Sa (*S. aureus*); Bs (*B. subtilis*); Ec (*E. coli*); Pa (*P. aeruginosa*); MRSA (*methicillin-resistant S. aureus*); Rs (*R. solani*); Ca (*C. albicans*); As (*A. solani*); Fg (*F. graminearum*); Vp (*V. pirina*).

## Data Availability

The study did not report any data.

## References

[1] Muellner-Riehl A.N., Rojas-Andrés B.M. (2022). Biogeography of Neotropical Meliaceae: Geological Connections, Fossil and Molecular Evidence Revisited. Rev. Bras. Bot..

[2] Luo J., Sun Y., Li Q., Kong L. (2022). Research Progress of Meliaceous Limonoids from 2011 to 2021. Nat. Prod. Rep..

[3] Naini A.A., Mayanti T., Harneti D., Darwati, Nurlelasari, Maharani R., Farabi K., Herlina T., Supratman U., Fajriah S. (2023). Sesquiterpenoids and Sesquiterpenoid Dimers from the Stem Bark of *Dysoxylum parasiticum* (Osbeck) Kosterm. Phytochemistry.

[4] Nishizawa M., Inoue A., Sastrapradja S., Hayashi Y. (1983). (+)-8-Hydroxycalamenene: A Fish-Poison Principle of *Dysoxylum acutangulum* and *D. alliaceum*. Phytochemistry.

[5] Traore M., Zhai L., Chen M., Olsen C.E., Odile N., Pierre G., Bosco O., Robert G., Christensen S.B. (2007). Cytotoxic Kurubasch Aldehyde from *Trichilia emetica*. Nat. Prod. Res..

[6] Harneti D., Ayu Permatasari A., Anisshabira A., Arofatus Naini A., Mayanti T., Maharani R., Safari A., Tatang Hidayat A., Farabi K., Supratman U. (2022). Sesquiterpenoids from the Stem Bark of *Aglaia grandis*. Nat. Prod. Sci..

[7] Ara I., Siddiqui B.S., Faizi S., Siddiqui S. (1989). Structurally Novel Diterpenoid Constituents from the Stem Bark of *Azadirachta indica* (Meliaceae). J. Chem. Soc. Perkin Trans. 1.

[8] Mulholland D.A., Monkhe T.V., Pegel K.H., Taylor D.A.H. (1999). Limonoids and Diterpenoids from *Dysoxylum spectabile* (Meliaceae). Biochem. Syst. Ecol..

[9] Gu J., Qian S.Y., Zhao Y.L., Cheng G.G., Hu D.B., Zhang B.H., Li Y., Liu Y.P., Luo X.D. (2014). Prenyleudesmanes, Rare Natural Diterpenoids from *Dysoxylum densiflorum*. Tetrahedron.

[10] Fang F.H., Huang W.J., Zhou S.Y., Han Z.Z., Li M.Y., Liu L.F., Wu X.Z., Yao X.J., Li Y., Yuan C.S. (2017). Aphapolins A and B: Two Nemoralisin Diterpenoids Isolated from *Aphanamixis polystachya* (Wall.) R. Parker. Eur. J. Org. Chem..

[11] Supratman U., Naibaho W., Salam S., Maharani R., Hidayat A.T., Harneti D., Nurlelasari, Shiono Y. (2019). Cytotoxic Triterpenoids from the Bark of *Chisocheton patens* Blume (Meliaceae). Phytochem. Lett..

[12] Kurimoto S.I., Takaishi Y., Ahmed F.A., Kashiwada Y. (2014). Triterpenoids from the Fruits of *Azadirachta indica* (Meliaceae). Fitoterapia.

[13] Harneti D., Tjokronegoro R., Safari A., Supratman U., Loong X.M., Mukhtar M.R., Mohamad K., Awang K., Hayashi H. (2012). Cytotoxic Triterpenoids from the Bark of *Aglaia smithii* (Meliaceae). Phytochem. Lett..

[14] Happi G.M., Talontsi F.M., Laatsch H., Zühlke S., Ngadjui B.T., Spiteller M., Kouam S.F. (2018). Seco-Tiaminic Acids B and C: Identification of Two Novel 3,4-Seco-Tirucallane Triterpenoids Isolated from the Root of *Entandrophragma congoënse* (Meliaceae). Fitoterapia.

[15] Zeng L., Gu Z.M., Chang C.J., Wood K.V., McLaughlin J.L. (1995). Meliavolkenin, a New Bioactive Triterpenoid from *Melia volkensii* (Meliaceae). Bioorganic Med. Chem..

[16] Tsamo A.T., Melong R., Mkounga P., Nkengfack A.E. (2019). Rubescins I and J, Further Limonoid Derivatives from the Stem Bark of *Trichilia rubescens* (Meliaceae). Nat. Prod. Res..

[17] Kowa T.K., Tchokouaha L.R.Y., Cieckiewicz E., Philips T.J., Dotse E., Wabo H.K., Tchinda A.T., Tane P., Frédérich M. (2020). Antileishmanial and Cytotoxic Activities of a New Limonoid and a New Phenyl Alkene from the Stem Bark of *Trichilia gilgiana* (Meliaceae). Nat. Prod. Res..

[18] Miranda R.N.C., Dolabela M.F., Da Silva M.N., Póvoa M.M., Maia J.G.S. (2012). Antiplasmodial Activity of the Andiroba (*Carapa guianensis* Aubl., Meliaceae) Oil and Its Limonoid-Rich Fraction. J. Ethnopharmacol..

[19] Supriatno, Nurlelasari, Herlina T., Harneti D., Maharani R., Hidayat A.T., Mayanti T., Supratman U., Azmi M.N., Shiono Y. (2018). A New Limonoid from Stem Bark of *Chisocheton pentandrus* (Meliaceae). Nat. Prod. Res..

[20] Liu J., Yang S.P., Su Z.S., Lin B.D., Wu Y., Yue J.M. (2011). Limonoids from the Stems of *Toona ciliata* Var. Henryi (Meliaceae). Phytochemistry.

[21] Peng L., Fu W.X., Zeng C.X., Zhou L., Bao M.F., Cai X.H. (2016). Two New Lignans from Twigs of *Aglaia odorata*. J. Asian Nat. Prod. Res..

[22] Wang L., Li F., Yang C.Y., Khan A.A., Liu X., Wang M.K. (2014). Neolignans, Lignans and Glycoside from the Fruits of *Melia toosendan*. Fitoterapia.

[23] Greger H., Pacher T., Brem B., Bacher M., Hofer O. (2001). Insecticidal Flavaglines and Other Compounds from *Fijian aglaia* Species. Phytochemistry.

[24] Harmon A.D., Weiss U., Silverton J.V. (1979). The Structure of Rohitukine, the Main Alkaloid of *Amoora rohituka* (Syn. *Aphanamixis polystachya*) (Meliaceae). Tetrahedron Lett..

[25] Kumar V., Gupta M., Gandhi S.G., Bharate S.S., Kumar A., Vishwakarma R.A., Bharate S.B. (2017). Anti-Inflammatory Chromone Alkaloids and Glycoside from *Dysoxylum binectariferum*. Tetrahedron Lett..

[26] Naini A.A., Mayanti T., Supratman U. (2022). Triterpenoids from Dysoxylum Genus and Their Biological Activities. Arch. Pharm. Res..

[27] Hilmayanti E., Nurlelasari, Supratman U., Kabayama K., Shimoyama A., Fukase K. (2022). Limonoids with Anti-Inflammatory Activity: A Review. Phytochemistry.

[28] Harneti D., Supratman U. (2021). Phytochemistry and Biological Activities of Aglaia Species. Phytochemistry.

[29] Modzelewska A., Sur S., Kumar S.K., Khan S.R. (2005). Sesquiterpenes: Natural Products That Decrease Cancer Growth. Curr. Med. Chem. Anti-Cancer Agents.

[30] Abidullah S., Rauf A., Khan S.W., Ayaz A., Liaquat F., Saqib S. (2022). A Comprehensive Review on Distribution, Paharmacological Uses and Biological Activities of *Argyrolobium roseum* (Cambess.). Jaub. Spach. Acta Ecol. Sin..

[31] Nagasampagi B.A., Yankov L., Dev S. (1968). Sesquiterpenoids from the Wood of *Cedrela toona* Roxb; Partial Synthesis of t-Muurolol, t-Cadinol and Cubenol; Structures of δ-Cadinene and δ-Cadinol. Tetrahedron Lett..

[32] Lago J.H.G., Soares M.G., Batista-Pereira L.G., Silva M.F.G.F., Corrêa A.G., Fernandes J.B., Vieira P.C., Roque N.F. (2006). Volatile Oil from *Guarea macrophylla* Ssp. Tuberculata: Seasonal Variation and Electroantennographic Detection by *Hypsipyla grandella*. Phytochemistry.

[33] Panshin A.J. (1933). Comparative Anatomy of the Woods of the Meliaceae, Sub-Family Swietenioideae. Am. J. Bot..

[34] Shu H.Z., Peng C., Bu L., Guo L., Liu F., Xiong L. (2021). Bisabolane-Type Sesquiterpenoids: Structural Diversity and Biological Activity. Phytochemistry.

[35] Kumeta Y., Ito M. (2016). Characterization of α-Humulene Synthases Responsible for the Production of Sesquiterpenes Induced by Methyl Jasmonate in Aquilaria Cell Culture. J. Nat. Med..

[36] Gaikwad N.W., Madyastha K.M. (2002). Biosynthesis of β-Substituted Furan Skeleton in the Lower Furanoterpenoids: A Model Study. Biochem. Biophys. Res. Commun..

[37] Xue S., Zhang P., Tang P., Wang C., Kong L., Luo J. (2020). Acyclic Diterpene and Norsesquiterpene from the Seed of *Aphanamixis polystachya*. Fitoterapia.

[38] Zhang R., He H.P., Di Y.T., Li S.L., Zuo G.Y., Zhang Y., Hao X.J. (2013). Chemical Constituents from *Aphanamixis grandifolia*. Fitoterapia.

[39] Krishnappa S., Dev S. (1973). Sesquiterpenes from Lansium Anamalayanum. Phytochemistry.

[40] Mulholland D.A., Iourine S., Taylor D.A.H. (1998). Sesquiterpenoids from Dysoxylum Schiffneri. Phytochemistry.

[41] Mulholland D.A., McFarland K., Randrianarivelojosia M. (2006). Sesquiterpenoid Derivatives from *Cipadessa boiviniana* (Meliaceae). Biochem. Syst. Ecol..

[42] Liu H.B., Zhang C.R., Dong S.H., Yang S.P., Sun Q., Geng M.Y., Yue J.M. (2012). Sesquiterpenes from *Dysoxylum oliganthum* and *Dysoxylum excelsum*. J. Asian Nat. Prod. Res..

[43] Febriandari R. (2013). Tukiran Suatu Senyawa Seskuiterpen Dari Ekstrak Kloroform Kulit Batang Tumbuhan *Aglaia elaeagnoidea* (A. Juss) Benth. UNESA J. Chem..

[44] Xia M.J., Zhang M., Li S.W., Cai Z.F., Zhao T.S., Liu A.H., Luo J., Zhang H.Y., Li J., Guo Y.W. (2022). Anti-Inflammatory and PTP1B Inhibitory Sesquiterpenoids from the Twigs and Leaves of *Aglaia lawii*. Fitoterapia.

[45] Lago J.H.G., Brochini C.B., Roque N.F. (2000). Terpenes from Leaves of *Guarea macrophylla* (Meliaceae). Phytochemistry.

[46] Núñez C.V., Roque N.F. (1999). Sesquiterpenes from the Stem Bark of *Guarea guidonia* (L.) Sleumer (Meliaceae). J. Essent. Oil Res..

[47] Pupo M.T., Adorno M.A.T., Vieira P.C., Fernandes J.B., Da Silva M.F.D.G.F., Piranic J.R. (2002). Terpenoids and Steroids from Trichilia Species. J. Braz. Chem. Soc..

[48] Garcez F.R., Núñez C.V., Garcez W.S., Almeida R.M., Roque N.F. (1998). Sesquiterpenes, Limonoid and Coumarin from the Wood Bark of *Guarea guidonia*. Planta Med..

[49] Mahdzir M.A., Shilpi J.A., Mahmud N., Ramasamy S., Awang K. (2017). Chemical Constituents from *Walsura pinnata* (Meliaceae). Nat. Prod. Commun..

[50] Nangmo K.P., Tsamo T.A., Zhen L., Mkounga P., Akone S.H., Tsabang N., Müller W.E.G., Marat K., Proksch P., Nkengfack A.E. (2018). Chemical Constituents from Leaves and Root Bark of *Trichilia monadelpha* (Meliaceae). Phytochem. Lett..

[51] Lago J.H.G., Reis A.A., Roque N.F. (2002). Chemical Composition from Volatile Oil of the Stem Bark of *Guarea macrophylla* Vahl. Ssp. *Tuberculata vellozo* (Meliaceae). Flavour Fragr. J..

[52] Ragasa C.Y., Labrador P., Rideout J.A. (2006). Antimicrobial Terpenoids from *Lansium domesticum*. Philipp. Agric. Sci..

[53] Sofian F.F., Subarnas A., Hakozaki M., Uesugi S., Koseki T., Shiono Y. (2022). Bidysoxyphenols A–C, Dimeric Sesquiterpene Phenols from the Leaves of *Dysoxylum parasiticum* (Osbeck) Kosterm. Fitoterapia.

[54] Zeng Q., Ye J., Cheng X., Qin J., Jin H., Zhang W.D. (2013). Chemical Constituents from *Aphanamixis grandifolia*. Chem. Nat. Compd..

[55] D’Ambola M., Malafronte N., Gualtieri M., Hernández V., Vassallo A., Severino L. (2016). A Novel Tirucallane-Type Triterpene and Sesquiterpene from Trichilia Maynasiana. Nat. Prod. Commun..

[56] Fadhilah K., Wahyuono S., Astuti P. (2021). A Sesquiterpene Aldehyde Isolated from Ethyl Acetate Extract of Lansium Domesticum Fruit Peel. Indones. J. Pharm..

[57] Lago J.H.G., Brochini C.B., Roque N.F. (2002). Terpenoids from *Guarea guidonia*. Phytochemistry.

[58] Brochini C.B., Roque N.F., Lago J.H.G. (2009). Minor Sesquiterpenes from the Volatile Oil from Leaves of *Guarea guidonia* Sleumer (Meliaceae). Nat. Prod. Res..

[59] Kurniasih N., Milawati H., Fajar M., Hidayat A.T., Abdulah R., Harneti D., Supratman U., Azmi M.N. (2018). Sesquiterpenoid Compounds from The Stembark of *Aglaia minahassae* (Meliaceae). Molekul.

[60] Fu L.R., Ma Q.Y., Huang S.Z., Dai H.F., Guo Z.K., Yu Z.F., Zhao Y.X. (2014). Terpenoids and Their Anti-Feedant Activity from *Cipadessa cinerascens*. J. Asian Nat. Prod. Res..

[61] Lin L.G., Tang C.P., Ke C.Q., Zhang Y., Ye Y. (2008). Terpenoids from the Stems of *Cipadessa baccifera*. J. Nat. Prod..

[62] Soares L.R., De Queiroz E Silva A.C., Freire T.V., Garcez F.R., Garcez W.S. (2012). Sesquiterpenos de Sementes de *Guarea guidonia* (Meliaceae). Quim. Nova.

[63] Chen H.D., Yang S.P., Liao S.G., Zhang B., Wu Y., Yue J.M. (2008). Limonoids and Sesquiterpenoids from *Amoora tsangii*. J. Nat. Prod..

[64] Sinaga S.E., Mayanti T., Naini A.A., Harneti D., Nurlelasari N., Maharani R., Farabi K., Supratman U., Fajriah S., Azmi M.N. (2022). Sesquiterpenoids from the Stem Bark of *Lansium domesticum* Corr. Cv. Kokossan and Their Cytotoxic Activity against MCF-7 Breast Cancer Cell Lines. Indones. J. Chem..

[65] Xie B.J., Yang S.P., Yue J.M. (2008). Terpenoids from *Dysoxylum densiflorum*. Phytochemistry.

[66] Chenda L.B.N., Kouam S.F., Lamshöft M., Kusari S., Talontsi F.M., Ngadjui B.T., Spiteller M. (2014). Isolation and Characterization of Six Labdane Diterpenes and One Pregnane Steroid of *Turraeanthus africanus*. Phytochemistry.

[67] Leite A.C., Ambrozin A.R.P., Fernandes J.B., Vieira P.C., Da Silva M.F.D.G.F., De Albuquerque S. (2008). Trypanocidal Activity of Limonoids and Triterpenes from *Cedrela fissilis*. Planta Med..

[68] Wang R., Liu D., Liu X., Liu F., Xuan L., Tang Y., Li W. (2022). Cytotoxicity and Polyol Pathway Inhibitory Activities of Chemical Constituents Isolated from the Pericarp of *Toona sinensis*. Nat. Prod. Res..

[69] Chen Y., Wang F., Ji C., Liu D., Liu X., Wang R., Li W. (2022). Chemical Constituents of the Pericarp of *Toona sinensis* and Their Chemotaxonomic Significance. Biochem. Syst. Ecol..

[70] Oktaviani D., Sukmawati W., Farabi K., Harneti D., Mahari R., Mayanti T., Safari A., Supratman U. (2022). Terpenoids from The Stem Bark of *Aglaia elaeagnoidea* and Their Cytotoxic Activity against HeLa and DU145 Cancer Cell Lines. Molekul.

[71] Liu S., Liu S.B., Zuo W.J., Guo Z.K., Mei W.L., Dai H.F. (2014). New Sesquiterpenoids from *Aglaia odorata* Var Microphyllina and Their Cytotoxic Activity. Fitoterapia.

[72] Parulian S.S., Nurlelasari, Naini A.A., Hilmayanti E., Mayanti T., Harneti D., Maharani R., Farabi K., Supratman U., Anwar R. (2022). Sesquiterpenoids from Stem Bark of *Chisocheton lasiocarpus* and Their Cytotoxic Activity against MCF-7 Breast Cancer Cell. Molekul.

[73] Pan L., Kardono L.B.S., Riswan S., Chai H., Carcache De Blanco E.J., Pannell C.M., Soejarto D.D., McCloud T.G., Newman D.J., Kinghorn A.D. (2010). Isolation and Characterization of Minor Analogues of Silvestrol and Other Constituents from a Large-Scale Re-Collection of *Aglaia foveolata*. J. Nat. Prod..

[74] Gu J., Qian S.Y., Cheng G.G., Li Y., Liu Y.P., Luo X.D. (2013). Chemical Components of *Dysoxylum densiflorum*. Nat. Products Bioprospect..

[75] Djemgou P.C., Gatsing D., Hegazy M.E.F., El-Hamd Mohamed A.H., Ngandeu F., Tane P., Ngadjui B.T., Fotso S., Laatsch H. (2010). Turrealabdane, Turreanone and an Antisalmonellal Agent from *Turraeanthus africanus*. Planta Med..

[76] Yang M.H., Wang J.S., Kong L.Y. (2012). Chemical Constituents of *Chisocheton cumingianus*. Chin. J. New Drugs.

[77] Naini A.A., Mayanti T., Nurlelasari, Harneti D., Maharani R., Safari A., Hidayat A.T., Farabi K., Lesmana R., Supratman U. (2022). Cytotoxic Sesquiterpenoids from *Dysoxylum parasiticum* (Osbeck) Kosterm. Stem Bark. Phytochem. Lett..

[78] Rodrigues V.F., Carmo H.M., Oliveira R.R., Filho R.B., Mathias L., Vieira I.J.C. (2009). Isolation of Terpenoids from *Trichilia quadrijuga* (Meliaceae) by Droplet Counter-Current Chromatography. Chromatographia.

[79] Zhang S.N., Huang L., Ma R.J., Yang M.F., Wei B.F., Song H.Z., Wang H.S., Tan Q.G. (2021). Chemical Constituents from the Barks of Melia Azedarach and Their PTP1B Inhibitory Activity. Nat. Prod. Res..

[80] Thị N., Ly T., Dũng N., Thị C., Trang K., Thu L., Diệu N., Hoa L. (2018). Sesqui-và Triterpenoid Từ vỏ Trái Bòn Bon (Lansium Domesticum). Tập.

[81] Hoffmann J.J., Cole J.R., Arora S.K., Bates R.B., Kriek G.R. (1978). Voleneol Diacetate: A New Sesquiterpenoid from *Lepidotrichilia volensii* Leroy (Meliaceae). J. Org. Chem..

[82] Hua W., Wang X., Li Q., Li Y., Cui L., Li Y., Kong L., Luo J. (2023). Rare Noreudesmane Sesquiterpenoids from Fruits of *Dysoxylum densiflorum*. Fitoterapia.

[83] Chowdhury R., Hasan C.M., Rashid M.A. (2003). Guaiane Sesquiterpenes from *Amoora rohituka*. Phytochemistry.

[84] Luo X., Wu S., Wu D. (2001). The Chemical Constituent of *Amoora yunnanensis*. J. Integr. Plant Biol..

[85] Garcez F.R., Garcez W.S., Da Silva A.F.G., De Cássia Bazzo R., Resende U.M. (2004). Terpenoid Constituents from Leaves of *Guarea kunthiana*. J. Braz. Chem. Soc..

[86] Lago J.H.G., Roque N.F. (2002). Cycloartane Triterpenoids from *Guarea macrophylla*. Phytochemistry.

[87] Lago J.H.G., Roque N.F. (2009). Estudo Fitoquímico Da Madeira de *Guarea macrophylla* (Meliaceae). Quim. Nova.

[88] Roux D., Martin M.T., Adeline M.T., Sevenet T., Hadi A.H.A., Païs M. (1998). Foveolins A and B, Dammarane Triterpenes from *Aglaia foveolata*. Phytochemistry.

[89] Pan L., Acuña U.M., Li J., Jena N., Ninh T.N., Pannell C.M., Chai H., Fuchs J.R., De Carcache Blanco E.J., Soejarto D.D. (2013). Bioactive Flavaglines and Other Constituents Isolated from *Aglaia perviridis*. J. Nat. Prod..

[90] Hou L., Tang G.H., Zhang Y., Hao X.J., Zhao Q., He H.P. (2013). A New Carotane Sesquiterpene from *Walsura robusta*. Chin. J. Nat. Med..

[91] Huang P.Q., Deng J.W., Li Y., Liao Z.B., Zhao E., Tian Y.C., Tu Y.D., Li D.L., Jin J.W., Zhou C.X. (2022). Terpenoids from the Twigs and Leaves of *Aglaia elaeagnoidea* and Their Chemotaxonomic Significance. Biochem. Syst. Ecol..

[92] De Paula J.R., Castro-Gamboa I., Neto J.O., Da Silva M.F.D.G.F., Fo E.R., Fernandes J.B., Vieira P.C., Pinheiro A.L. (1998). Chemistry of *Cedrela odorata* Graft and Speculations on the Induced Resistance against *Hypsipyla grandella*. An. Acad. Bras. Cienc..

[93] Dharmayani N.K.T., Yoshimura T., Hermawati E., Juliawaty L.D., Syah Y.M. (2020). Antibacterial and Antifungal Two Phenolic Sesquiterpenes from *Dysoxylum densiflorum*. Z. Für Nat. C.

[94] Daniewski W.M., Anczewski W., Gumułka M., Danikiewicz W., Jacobsson U., Norin T. (1996). Sesquiterpenoid Constituents of *Entandrophragma cylindricum*. Phytochemistry.

[95] Milawati H., Harneti D., Maharani R., Hidayat A.T., Azmi M.N., Shiono Y., Supratman U., Sciences N., Padjadjaran U., Science E. (2019). Caryophyllene-Type Sesquiterpenoids from the Stembark of *Aglalia harmsiana* and Their Cytotoxic Activity Against MCF-7 Breast Cancer Cells. Molekul.

[96] Benosman A., Richomme P., Sevenet T., Hamid A., Hadi A., Bruneton J. (1994). Secotirucallane Triterpenes from the Stem Bark of *Aglaia leucophylla*. Phytochemistry.

[97] Napagoda M., Gerstmeier J., Koeberle A., Wesely S., Popella S., Lorenz S., Scheubert K., Böcker S., Svatoš A., Werz O. (2014). *Munronia pinnata* (Wall.) Theob.: Unveiling Phytochemistry and Dual Inhibition of 5-Lipoxygenase and Microsomal Prostaglandin E_2_ Synthase (MPGES)-1. J. Ethnopharmacol..

[98] Kaneda N., Pezzuto J.M., Kinghorn D., Farnsworth N.R., Santisuk T., Tuchinda P., Udchachon J., Reutrakul V. (1992). Plant Anticancer Agents, l. Cytotoxic Triterpenes from *Sandoricum koetjape* Stems. J. Nat. Prod..

[99] Izdihar G., Naini A.A., Harneti D., Maharani R., Nurlelasari N., Mayanti T., Safari A., Farabi K., Supratman U., Azmi M.N. (2021). Sesquiterpenoids from the Stem Bark of *Aglaia simplicifolia* and Their Cytotoxic Activity against B16-F10 Melanoma Skin Cancer Cell. Indones. J. Chem..

[100] Nishizawa M., Inoue A., Hayashi Y., Sastrapradja S., Kosela S., Iwashita T. (1984). Structure of Aphanamol I and II. J. Org. Chem..

[101] Vieira I.J.C., Figueiredo E.R., Freitas V.R., Mathias L., Braz-Filho R., Araújo R.M. (2010). A New Sesquiterpene from *Trichilia casarettii* (Meliaceae). Am. J. Anal. Chem..

[102] Pointinger S., Promdang S., Vajrodaya S., Pannell C.M., Hofer O., Mereiter K., Greger H. (2008). Silvaglins and Related 2,3-Secodammarane Derivatives—Unusual Types of Triterpenes from *Aglaia silvestris*. Phytochemistry.

[103] Sofian F.F., Subarnas A., Hakozaki M., Uesugi S., Koseki T., Shiono Y. (2022). Tridysoxyphenols A and B, Two New Trimeric Sesquiterpene Phenols from *Dysoxylum parasiticum* Leaves. Phytochem. Lett..

[104] Russell G.B., Hunt M.B., Bowers W.S., Blunt J.W. (1994). A Sesquiterpenoid Ant Repellent from *Dysoxylum spectabile*. Phytochemistry.

[105] Joycharat N., Plodpai P., Panthong K., Yingyongnarongkul B.E., Voravuthikunchai S. (2010). Terpenoid Constituents and Antifungal Activity of *Aglaia forbesii* Seed against Phytopathogens. Can. J. Chem..

[106] Yuan H.W., Zhao J.P., Liu Y.B., Qiu Y.X., Xie Q.L., LI M.J., Khan I.A., Wang W. (2018). Advance in Studies on Chemical Constituents, Pharmacology and Quality Control of *Aquilaria sinensis*. Digit. Chin. Med..

[107] Vieira I.J.C., De Aquino Azevedo O., De Souza J.J., Braz-Filho R., Dos Santos Gonçalves M., De Araújo M.F. (2013). Hirtinone, a Novel Cycloartane-Type Triterpene and Other Compounds from *Trichilia hirta* L. (Meliaceae). Molecule.

[108] Joycharat N., Greger H., Hofer O., Saifah E. (2008). Flavaglines and Triterpenoids from the Leaves of *Aglaia forbesii*. Phytochemistry.

[109] Nugroho A.E., Sugiura R., Momota T., Hirasawa Y., Wong C.P., Kaneda T., Hadi A.H.A., Morita H. (2015). Dysosesquiflorins A and B, Sesquiterpenoids from *Dysoxylum densiflorum*. J. Nat. Med..

[110] Phongmaykin J., Kumamoto T., Ishikawa T., Suttisri R., Saifah E. (2008). A New Sesquiterpene and Other Terpenoid Constituents of *Chisocheton penduliflorus*. Arch. Pharm. Res..

[111] Inada A., Shono K., Murata H., Inatomi Y., Darnaedi D. (2000). Three Putrescine Bisamides from the Leaves of *Aglaia grandis*. Phytochemistry.

[112] Yuan C.M., Tang G.H., Wang X.Y., Zhang Y., Cao M.M., Li X.H., Li Y., Li S.L., Di Y.T., He H.P. (2013). New Steroids and Sesquiterpene from *Turraea pubescens*. Fitoterapia.

[113] Stærk D., Skole B., Jørgensen F.S., Budnik B.A., Ekpe P., Jaroszewski J.W. (2004). Isolation of a Library of Aromadendranes from *Landolphia dulcis* and Its Characterization Using the Volsurf Approach. J. Nat. Prod..

[114] Fareza M.S., Nurlelasari, Supratman U., Katja D.G., Husna M.H., Awang K. (2019). 1,1,4,7-Tetramethyldecahydro-1H-Cyclopropa[E]Azulen-7-Ol From the Stembark *Chisocheton pentandrus*. Molbank.

[115] Doe M., Hirai Y., Kinoshita T., Shibata K., Haraguchi H., Morimoto Y. (2004). Structure, Synthesis, and Biological Activity of 14-Methoxy-1,2-Dehydrocacalol Methyl Ether, a New Modified Furanoeremophilane Type Sesquiterpene from *Trichilia cuneata*. Chem. Lett..

[116] Doe M., Shibue T., Haraguchi H., Morimoto Y. (2005). Structures, Biological Activities, and Total Syntheses of 13-Hydroxy- and 13-Acetoxy-14-Nordehydrocacalohastine, Novel Modified Furanoeremophilane-Type Sesquiterpenes from *Trichilia cuneata*. Org. Lett..

[117] Hapuarachchi S.D., Suresh T.S., Hadunnetthi S., PriyanthieSandun Kumari Senarath W.T., Ranasinghe C. (2022). Anti-Inflammatory Potential of Aqueous Extract and Ethyl Acetate Fractions of *Munronia pinnata* (Wall) Theob. and the Isolated Compound, Senecrassidiol. Trends Phytochem. Res..

[118] Ngo N.T.N., Lai N.T.D.D.T., Le H.C., Nguyen L.T.T., Trinh B.T.D., Nguyen H.D., Pham P.D., Dang S.V., Nguyen L.H.D. (2022). Chemical Constituents of *Aglaia elaeagnoidea* and *Aglaia odorata* and Their Cytotoxicity. Nat. Prod. Res..

[119] Nishizawa M., Yamada H., Sastrapradja S., Hayashi Y. (1985). Structure and Synthesis of Bicalamenene. Tetrahedron Lett..

[120] Naini A.A., Mayanti T., Maharani R., Fajriah S., Kabayama K., Shimoyama A., Manabe Y., Fukase K., Jungsuttiwong S., Supratman U. (2023). Dysoticans F-H: Three Unprecedented Dimeric Cadinanes from *Dysoxylum parasiticum* (Osbeck) Kosterm. Stem Bark. RSC Adv..

[121] Xavier S.M., Nair G., Sabulal B., Mathew J. (2011). Chemical Constituent and Antibacterial Activity of the Root Oil of *Naregamia alata*. J. Trop. Med. Plants.

[122] Guo Y.Y., Wang J., Zhang X.B., Shang X., Gong S., Zhang K. (2016). Chemical Composition of the Essential Oils of *Swietenia macrophylla* from China. Chem. Nat. Compd..

[123] Dougnon G., Ito M. (2022). Essential Oils from *Melia azedarach* L. (Meliaceae) Leaves: Chemical Variability upon Environmental Factors. J. Nat. Med..

[124] Geetha B.S., Garden T.B. (2008). Essential Oil Composition of *Naregamia alata*. J. Med. Aromat. Plant Sci..

[125] Wang C., Shi J.-X., Wu Y. (2014). Chemical and Antimicrobial Analyses of Essential Oil of *Toona sinensis* from China. Asian J. Chem..

[126] Kharkwal G.C., Pande C., Tewari G., Panwar A., Pande V. (2015). Volatile Terpenoid Composition and Antimicrobial Activity of Flowers of *Melia azedarach* Linn, from North West Himalayas, India. J. Indian Chem. Soc..

[127] Wu J.G., Peng W., Yi J., Wu Y.B., Chen T.Q., Wong K.H., Wu J.Z. (2014). Chemical Composition, Antimicrobial Activity against *Staphylococcus aureus* and a pro-Apoptotic Effect in SGC-7901 of the Essential Oil from *Toona sinensis* (A. Juss.) Roem. Leaves. J. Ethnopharmacol..

[128] Hardt I.H., Rieck A., Fricke C., Konigt W.A. (1995). Enantiomeric Composition of Sesquiterpene Hydrocarbons of the Essential Oil of *Cedrela odorata* L.. Flavour Fragr. J..

[129] Hsieh T.J., Tsai Y.H., Liao M.C., Du Y.C., Lien P.J., Sun C.C., Chang F.R., Wu Y.C. (2012). Anti-Diabetic Properties of Non-Polar *Toona sinensis* Roem Extract Prepared by Supercritical-CO_2_ Fluid. Food Chem. Toxicol..

[130] Kumar R., Verma G., Prakash O., Pant A.K. (2011). Head Space GC/MS Analysis of Volatile Constituents of *Trichilea connaroides* Wight and Arn. Extracts and Their in Vitro Anti-Plasmodium Activity against *Plasmodium falciparum* Isolates. Res. J. Phytochem..

[131] Kavitha K., Bopaiah A., Kolar A.B. (2016). Chemical Composition Of The Essential Oil From The Leaves Of *Cipadessa baccifera* (ROTH.) MIQ. Int. J. Pharm. Sci. Res..

[132] Agarwal G., Pant A.K. (2010). Volatile Constituents of *Trichilia connaroides* (Wight and Arn.) Roots. Asian J. Tradit. Med..

[133] El Souda S.S., Aboutabl E.A., Maamoun A.A., Hashem F.A. (2016). Volatile Constituents and Cytotoxic Activity of *Khaya grandifoliola* and *Khaya senegalensis* Flower Extracts. J. Herbs Spices Med. Plants.

[134] Thangadurai D., Nagalakshmi M.A.H., Pulliah T., Ratnam B.V.V. (2003). Essential Oils of the Leaves of Chukrasia Tabularis Collected from the Eastern Ghats of Peninsular India. J. Essent. Oil Res..

[135] Asekun O.T., Ekundayo O. (1999). Constituents of the Leaf Essential Oil of *Cedrela odorata* L. from Nigeria. Flavour Fragr. J..

[136] Maia B.H.L.N.S., De Paula J.R., Sant’Ana J., Da Silva M.F.D.G.F., Fernandes J.B., Vieira P.C., Costa M.D.S.S., Ohashi O.S., Silva J.N.M. (2000). Essential Oils of Toona and Cedrela Species (Meliaceae): Taxonomic and Ecological Implications. J. Braz. Chem. Soc..

[137] Lago J.H.G., de Avila P., de Aquino E.M., Moreno P.R.H., Ohara T. M.T., Limberger R.P., Apel M.A., Henriques A.T. (2004). Volatile Oils from Leaves and Stem Barks of *Cedrela fissilis* (Meliaceae): Chemical Composition and Antibacterial Activities. Flavour Fragr. J..

[138] Chen Y.C., Hsieh C.L., Huang B.M., Chen Y.C. (2021). Induction of Mitochondrial-Dependent Apoptosis by Essential Oil of *Toona sinensis* Root through Akt, Mtor and Nf-Kb Signalling Pathways in Human Renal Cell Carcinoma Cells. J. Food Drug Anal..

[139] Shilaluke K.C., Moteetee A.N. (2022). Insecticidal Activities and GC-MS Analysis of the Selected Family Members of Meliaceae Used Traditionally as Insecticides. Plants.

[140] Soares M.G., Batista-Pereira L.G., Fernandes J.B., Corrêa A.G., Da Silva M.F.G.F., Vieira P.C., Rodrigues Filho E., Ohashi O.S. (2003). Electrophysiological Responses of Female and Male *Hypsipyla grandella* (Zeller) to Swietenia Macrophylla Essential Oils. J. Chem. Ecol..

[141] Ogunwande I.A., Ekundayo O., Olawore N.O., Adeleke K.A. (2005). Constituents of the Essential Oils of the Leaves and Stem Bark of *Cedrela mexicana* L. Grown in Nigeria. J. Essent. Oil Res..

[142] Chen M., Wang C., Li L., Wang Y., Jiang S. (2010). Retention of Volatile Constituents in Dried *Toona sinensis* by GC-MS Analysis. Int. J. Food Eng..

[143] Duan D., Chen L., Yang X., Tu Y., Jiao S. (2015). Antidepressant-like Effect of Essential Oil Isolated from *Toona ciliata* Roem. Var. Yunnanensis. J. Nat. Med..

[144] Joycharat N., Thammavong S., Voravuthikunchai S.P., Plodpai P., Mitsuwan W., Limsuwan S., Subhadhirasakul S. (2014). Chemical Constituents and Antimicrobial Properties of the Essential Oil and Ethanol Extract from the Stem of *Aglaia odorata* Lour. Nat. Prod. Res..

[145] Aromdee C., Anorach R., Sriubolmas N. (2005). Essential Oil of the Flower of *Azadirachta indica* (Meliaceae). Acta Hortic..

[146] Siddiqui B.S., Ali S.T., Rajput M.T., Gulzar T., Rasheed M., Mehmood R. (2009). GC-Based Analysis of Insecticidal Constituents of the Flowers of *Azadirachta indica* A. Juss. Nat. Prod. Res..

[147] Soares M.G., Fernandes J.B., Lago J.H.G. (2010). Interspecific Variation In The Composition Of Volatile Oils From The Leaves Of *Swietenia macrophylla* King (Meliaceae). Quim. Nova.

[148] Magalhães L.A.M.I., Da Paz Lima M., Marques M.O.M., Facanali R., Da Silva Pinto A.C., Tadei W.P. (2010). Chemical Composition and Larvicidal Activity against Aedes Aegypti Larvae of Essential Oils from Four Guarea Species. Molecules.

[149] Pandini J.A., Pinto F.G.S., Scur M.C., Santana C.B., Costa W.F., Temponi L.G. (2018). Composição Química, Potencial Antimicrobiano e Antioxidante Do Óleo Essencial de *Guarea kunthiana* A. Juss. Brazil. J. Biol..

[150] Oliveira E.A., Martins E.G.A., Soares M.G., Chagas-Paula D.A., Passero L.F.D., Sartorelli P., Baldim J.L., Lago J.H.G. (2019). A Comparative Study on Chemical Composition, Antileishmanial and Cytotoxic Activities of the Essential Oils from Leaves of *Guarea macrophylla* (Meliaceae) from Two Different Regions of São Paulo State, Brazil, Using Multivariate Statistical Analysis. J. Braz. Chem. Soc..

[151] Lago J.H.G., Roque N.F. (2015). Analysis of the Essential Oil from Leaves of Three Different Specimens of *Guarea guidonia* (L.) Sleumer (Meliaceae). J. Essent. Oil Res..

[152] Menut C., Lamaty G., Bessière J.M., Seuleiman A.M., Fendero P., Maidou E., Dénamganai J. (1995). Aromatic Plants of Tropical Central Africa. XXI. Chemical Composition of Bark Essential Oil of *Guarea cedrata* (A. Chev.) Pellegr. from Central African Republic. J. Essent. Oil Res..

[153] Ribeiro W.H.F., Arriaga Â.M.C., Andrade-Neto M., Vasconcelos J.N., Santiago G.M.P., Nascimento R.F. (2006). Composition of the Essential Oil of *Guarea macrophylla* Vahl. Ssp. Tuberculata (Meliaceae) from Northeast of Brazil. J. Essent. Oil Res..

[154] Nogueira T.S.R., de Passos M.S., Nascimento L.P.S., de Arantes M.B.S., Monteiro N.O., da Boeno S.I.S., de Carvalho Junior A., de Azevedo O.A., da Terra W.S., Vieira M.G.C. (2020). Chemical Compounds and Biologic Activities: A Review of Cedrela Genus. Molecules.

[155] Nuñez C.V., Lago J.H.G., Roque N.F. (2005). Variation on the Chemical Composition of the Oil from Damaged Branches of *Guarea guidonia* (L.) Sleumer (Meliaceae). J. Essent. Oil Res..

[156] Wang G.W., Jin H.Z., Zhang W.D. (2013). Constituents from Aphanamixis Species and Their Biological Activities. Phytochem. Rev..

[157] Bandi A.K.R., Dong-Ung L. (2012). Secondary Metabolites of Plants from the Genus Cipadessa: Chemistry and Biological Activity. Chem. Biodivers..

[158] Chen J.L., Kernan M.R., Jolad S.D., Stoddart C.A., Bogan M., Cooper R. (2007). Dysoxylins A-D, Tetranortriterpenoids with Potent Anti-RSV Activity from *Dysoxylum gaudichaudianum*. J. Nat. Prod..

[159] Safriansyah W., Sinaga S.E., Supratman U., Harneti D. (2022). Phytochemistry and Biological Activities of Guarea Genus (Meliaceae). Molecules.

[160] Negi J.S., Bisht V.K., Bhandari A.K., Bharti M.K., Sundriyal R.C. (2011). Chemical and Pharmacological Aspects of Toona (Meliaceae). Res. J. Phytochem..

[161] Curcino Vieira I.J., da Silva Terra W., dos Santos Gonçalves M., Braz-Filho R. (2014). Secondary Metabolites of the Genus Trichilia: Contribution to the Chemistry of Meliaceae Family. Am. J. Anal. Chem..

[162] Oyedeji-Amusa M.O., Sadgrove N.J., Van Wyk B.E. (2021). The Ethnobotany and Chemistry of South African Meliaceae: A Review. Plants.

[163] Voravuthikunchai S.P., Kanchanapoom T., Sawangjaroen N., Hutadilok-Towatana N. (2010). Antioxidant, Antibacterial and Antigiardial Activities of *Walsura robusta* Roxb. Nat. Prod. Res..

[164] Kristianslund R., Aursnes M., Tungen J.E., Görbitz C.H., Hansen T.V. (2018). Synthesis, Biological Investigation, and Structural Revision of Sielboldianin A. J. Nat. Prod..

[165] Ellithey M.S., Lall N., Hussein A.A., Meyer D. (2013). Cytotoxic, Cytostatic and HIV-1 PR Inhibitory Activities of the Soft Coral *Litophyton arboreum*. Mar. Drugs.

[166] Shi D., Song X., Guo Y., Xu J., Liu Y., Zhang J., Cui C.A., Jin D.Q. (2017). Alismol, a Sesquiterpenoid Isolated from Vladimiria Souliei, Suppresses Proinflammatory Mediators in Lipopolysaccharide-Stimulated Microglia. J. Mol. Neurosci..

[167] Zhang H.J., Tan G.T., Santarsiero B.D., Mesecar A.D., Van Hung N., Cuong N.M., Soejarto D.D., Pezzuto J.M., Fong H.H.S. (2003). New Sesquiterpenes from *Litsea verticillata*. J. Nat. Prod..

[168] Ding L., Pfoh R., Rühl S., Qin S., Laatsch H. (2009). T-Muurolol Sesquiterpenes from the Marine *Streptomyces* Sp. M491 and Revision of the Configuration of Previously Reported Amorphanes. J. Nat. Prod..

[169] Akiel M.A., Alshehri O.Y., Aljihani S.A., Almuaysib A., Bader A., Al-Asmari A.I., Alamri H.S., Alrfaei B.M., Halwani M.A. (2022). Viridiflorol Induces Anti-Neoplastic Effects on Breast, Lung, and Brain Cancer Cells through Apoptosis. Saudi J. Biol. Sci..

[170] Tan M., Zhou L., Huang Y., Wang Y., Hao X., Wang J. (2008). Antimicrobial Activity of Globulol Isolated from the Fruits of *Eucalyptus globulus* Labill. Nat. Prod. Res..

[171] Ayyad S.E.N., Alarif W.M., Al-Footy K.O., Selim E.A., Ghandourah M.A., Aly M.M., Alorfi H.S. (2017). Isolation, Antimicrobial and Antitumor Activities of a New Polyhydroxysteroid and a New Diterpenoid from the Soft Coral Xenia Umbellata. Z. Für Naturforschung C..

[172] Tseng Y.J., Shen K.P., Lin H.L., Huang C.Y., Dai C.F., Sheu J.H. (2012). Lochmolins A-G, New Sesquiterpenoids from the Soft Coral Sinularia Lochmodes. Mar. Drugs.

[173] Kurniasih N., Supriadin A., Fajar M., Abdulah R., Harneti D., Supratman U., Taib M.N.A.B.M. (2019). Cytotoxic Sesquterpenoid Compound from the Stembark of *Aglaia simplicifolia* (Meliaceae). J. Phys. Conf. Ser..

[174] Fernandes E.S., Passos G.F., Medeiros R., da Cunha F.M., Ferreira J., Campos M.M., Pianowski L.F., Calixto J.B. (2007). Anti-Inflammatory Effects of Compounds Alpha-Humulene and (−)-Trans-Caryophyllene Isolated from the Essential Oil of *Cordia verbenacea*. Eur. J. Pharmacol..

[175] Zhang X., Li G., Deng Q., Xu Z., Cen J., Xu J. (2021). Vomifoliol Isolated from Mangrove Plant Ceriops Tagal Inhibits the NFAT Signaling Pathway with CN as the Target Enzyme in Vitro. Bioorganic Med. Chem. Lett..

[176] Sadiqi S., Hamza M., Ali F., Alam S., Shakeela Q., Ahmed S., Ayaz A., Ali S., Saqib S., Ullah F. (2022). Molecular Characterization of Bacterial Isolates from Soil Samples and Evaluation of Their Antibacterial Potential against MDRS. Molecules.

[177] Petrera E. (2015). Antiviral and Immunomodulatory Properties of Meliaceae Family. J. Biol. Act. Prod. Nat..

[178] Tajuddeen N., Van Heerden F.R. (2019). Antiplasmodial Natural Products: An Update. Malar. J..

